# Efficacy of selected dietary supplements and pharmacological agents on metabolic and oxidative stress outcomes in metabolic dysfunction–associated fatty liver disease (MAFLD): a Bayesian network meta-analysis

**DOI:** 10.3389/fphar.2025.1682688

**Published:** 2026-01-29

**Authors:** Yunyi Yang, Xiaoli He, Jiayuan Cai, Xiaoxiao Qu, Shufa Tan, Jiawen You, Yanming He, Zheng Yao, Hongjie Yang

**Affiliations:** 1 Yueyang Hospital of Integrated Traditional Chinese and Western Medicine, Shanghai University of Traditional Chinese Medicine, Shanghai, China; 2 Shuguang Hospital Affiliated to Shanghai University of Traditional Chinese Medicine, Shanghai, China; 3 College of Integrated Traditional Chinese and Western Medicine, Tianjin University of Traditional Chinese Medicine, Tianjin, China

**Keywords:** Bayesian network meta-analysis, dietary supplements, metabolic dysfunction-associated fatty liver disease (MAFLD), pharmacological agents, randomized controlled trials (RCTs)

## Abstract

**Objective:**

This study aimed to compare the relative efficacy of dietary supplements and pharmacological agents in improving metabolic and oxidative stress outcomes in patients with metabolic dysfunction-associated fatty liver disease (MAFLD) through a systematic review and Bayesian network meta-analysis.

**Methods:**

PubMed, Embase, the Cochrane Library, and Web of Science databases were systematically searched to identify randomized controlled trials (RCTs) published between 1 January 2014, and 3 March 2024, that evaluated the effects of dietary supplements or pharmacological agents on MAFLD.

**Results:**

A total of 106 RCTs involving 7,273 participants were included, involving a variety of nutritional and pharmacological interventions. Pharmacological intervention plus bioactive regulator was the most effective in reducing triglycerides (TG) and low-density lipoprotein cholesterol (LDL-C). Nutrient plus pharmacological intervention was the most efficacious in improving glycated hemoglobin (HbA1c) and insulin levels. Pharmacological intervention plus another pharmacological intervention was most effective in reducing alanine aminotransferase (ALT) and aspartate aminotransferase (AST). Phytochemicals significantly reduced interleukin-6 (IL-6), while bioactive metabolic regulators markedly decreased tumor necrosis factor-α (TNF-α). Herbal extracts demonstrated superior effects in enhancing total antioxidant capacity (TAC) and superoxide dismutase (SOD), while oleoylethanolamide (OEA) improved antioxidant enzyme activity and reduced malondialdehyde (MDA) levels. Further analysis revealed that garlic exerted the strongest effect in improving overall antioxidant capacity, whereas L-carnitine showed the most significant efficacy in suppressing lipid peroxidation.

**Conclusion:**

Multicomponent or mechanistically complementary interventions exhibited more stable potential benefits in improving glucose and lipid metabolism. Bioactive metabolic regulators (L-carnitine), endogenous lipid regulators (OEA), and herbal extracts (garlic) all demonstrated strong efficacy in enhancing antioxidant defense and alleviating oxidative damage. Further high-quality RCTs are needed to confirm these findings.

**Systematic Review Registration:**

https://www.crd.york.ac.uk/PROSPERO/, identifier CRD42024555065.

## 1 Introduction

Metabolic dysfunction-associated fatty liver disease (MAFLD) is a liver disorder closely linked to systemic metabolic dysregulation. In 2020, the term MAFLD was introduced to replace non-alcoholic fatty liver disease (NAFLD) (Gofton et al., 2023). Unlike NAFLD, which is primarily diagnosed by excluding alcohol consumption, the diagnosis of MAFLD requires the presence of metabolic abnormalities, such as insulin resistance, obesity, and hyperglycemia, in addition to hepatic steatosis (Pipitone et al., 2023; Zeybel et al., 2022). The disease spectrum encompasses simple steatosis, nonalcoholic steatohepatitis (NASH), hepatic fibrosis, cirrhosis, and ultimately hepatocellular carcinoma (HCC) (Friedman et al., 2018). MAFLD affects approximately one-quarter of the adult population and has emerged as a major public health concern worldwide (Abeysekera et al., 2020). Therefore, exploring effective interventions to delay its disease progression and reduce the risk of metabolic and cardiovascular complications is imperative.

The pathogenesis of MAFLD is multifactorial, involving insulin resistance, lipid metabolism disorders, oxidative stress, inflammatory responses, and gut microbiota dysbiosis (Kuchay et al., 2020). Among these, oxidative stress is considered a key contributor to the onset and progression of MAFLD (Chen et al., 2020). In genetically predisposed individuals, chronic excess energy intake and sedentary lifestyles promote the accumulation of hepatic lipids, accompanied by mitochondrial and endoplasmic reticulum dysfunction, leading to excessive production of reactive oxygen species (ROS). This, in turn, triggers lipid peroxidation and the accumulation of toxic metabolites such as malondialdehyde (MDA), exacerbating hepatocellular injury and chronic inflammation (Seidita et al., 2024). Furthermore, lifestyle modification, particularly weight control, healthy diet, and increased physical activity, remains the cornerstone of the prevention and management of MAFLD (Spagnuolo et al., 2019; European Association for the Study of the Liver, 2016). However, in clinical practice, some patients respond inadequately to lifestyle interventions alone or fail to sustain long-term adherence. As a result, pharmacological and nutritional adjunct therapies have become increasingly important in the management of MAFLD.

Currently, pharmacological interventions for MAFLD include insulin sensitizers, lipid-lowering agents, antioxidants, hepatoprotective drugs, and anti-inflammatory compounds (Stefan et al., 2019). Although some of these agents have demonstrated beneficial effects in clinical trials, their long-term efficacy, safety, and tolerability remain concerning (Li et al., 2022). Meanwhile, dietary supplements derived from natural products or functional nutrients, which can regulate multiple targets, show potential benefits in relieving the accumulation of hepatic lipids, oxidative stress, and inflammation (Dongiovanni et al., 2016). Recent studies have examined the therapeutic effects of various dietary supplements and pharmacological agents in MAFLD patients. Dietary supplements may exert anti-inflammatory and antioxidant effects through mechanisms such as modulating the gut–liver axis, influencing energy absorption, improving bile acid metabolism, restoring intestinal barrier function, and reducing the production of endogenous ethanol (Yao et al., 2022; Ferro D. et al., 2020). Although natural compounds such as vitamin E, vitamin D, silymarin, and curcumin have been demonstrated to improve lipid profiles, insulin sensitivity, liver function, and oxidative stress markers in randomized controlled trials (RCTs), their clinical evidence remains inconsistent (Rizzo et al., 2023), with limited sample sizes. Furthermore, systematic comparisons among intervention types are lacking.

Network meta-analysis (NMA) can integrate direct and indirect evidence to simultaneously compare multiple therapeutic interventions (Liu et al., 2019). Therefore, we conducted a systematic review and Bayesian NMA to compare the relative efficacy of different dietary supplements and pharmacological agents in improving metabolic, hepatic, inflammatory, and oxidative stress outcomes in patients with MAFLD, aiming to provide theoretical evidence for individualized clinical intervention strategies.

## 2 Methods

### 2.1 Search process and strategy

This study was conducted in accordance with the *Preferred Reporting Items for Systematic Reviews and Meta-Analyses (PRISMA)* guidelines (Page et al., 2021) and was registered with the International Prospective Register of Systematic Reviews (PROSPERO; Registration No. CRD42024555065).

PubMed, Embase, Cochrane Library, and Web of Science databases were systematically searched to collect RCTs published between 1 January 2014 and 3 March 2024. Studies evaluating the effects of dietary supplements or pharmacological agents on metabolic and oxidative stress outcomes in patients with MAFLD were eligible.

Given that dietary supplements and pharmacological agents often exhibit overlapping antioxidant, anti-inflammatory, or metabolic regulatory effects, the search strategy encompassed representative drugs and naturally derived bioactive compounds with these mechanisms to ensure comprehensive coverage of relevant clinical interventions.

Both medical subject headings (MeSH) and free-text terms were used in combination to design the search strategy. The detailed search strategy was as follows: [(“Metabolic dysfunction-associated fatty liver disease”) OR (“MAFLD”) OR (“Nonalcoholic fatty liver disease”) OR (“NAFLD”) OR (“Non-alcoholic steatohepatitis”) OR (“NASH”)] AND [(“Dietary supplements”) OR (“Nutritional supplements”) OR (“Bioactive compounds”) OR (“Phytochemicals”) OR (“Herbal extracts”) OR (“Vitamins”) OR (“Minerals”) OR (“Vitamin E”) OR (“Vitamin D″) OR (“Coenzyme Q10”) OR (“L-carnitine”) OR (“Curcumin”) OR (“Resveratrol”) OR (“Oleoylethanolamide”) OR (“Silymarin”) OR (“Essentiale forte”) OR (“Antioxidants”) OR (“Pharmacological treatment”) OR (“Pharmacological agents”) OR (“Drugs”) OR (“Insulin sensitizers”) OR (“Lipid-lowering agents”) OR (“Pioglitazone”) OR (“Pentoxifylline”) OR (“Ursodeoxycholic acid”) OR (“Fenofibrate”) OR (“Metformin”))] AND [(“Randomized controlled trial”) OR (“RCT”) OR (“Clinical trial”)]. The search strategy was appropriately adapted for each database according to its specific structure and requirements. To ensure the comprehensiveness and accuracy of the included studies, we also manually screened the reference lists of eligible articles and relevant systematic reviews to identify any additional studies that might have been missed during the electronic search.

### 2.2 Inclusion and exclusion criteria

The eligibility criteria were designed based on the PICOS framework (participants, interventions, comparators, outcomes, and study design).

#### 2.2.1 Study design

Peer-reviewed RCTs were included, regardless of whether they were single-center or multicenter studies.

#### 2.2.2 Participants

Adult patients (≥18 years) diagnosed with MAFLD or NAFLD by imaging, laboratory examination, or histopathology were included, without restrictions on sex or ethnicity.

#### 2.2.3 Interventions

The intervention group received dietary supplements or pharmacological agents with antioxidant or metabolic regulatory properties. Dietary supplements comprised vitamins, plant extracts, coenzymes, and metabolic regulators. Pharmacological agents included lipid-lowering drugs, insulin sensitizers, hepatoprotective agents, and other drugs related to metabolic or antioxidant regulation. Some pharmacological agents, such as pioglitazone, ursodeoxycholic acid, and pentoxifylline, are not classical antioxidants but exert metabolic and oxidative stress regulatory effects by activating PPAR pathways, enhancing fatty acid β-oxidation, improving insulin sensitivity, and reducing inflammation. Therefore, pharmacological agents with such mechanisms were analyzed together with dietary supplements to comprehensively evaluate the comparative efficacy of different interventions on metabolic, inflammatory, and oxidative stress outcomes. Trials on both monotherapy and combination therapy were included.

#### 2.2.4 Comparators

The control group received placebo, standard treatment, or other active interventions. For multi-arm studies, all eligible comparisons were incorporated into the NMA.

#### 2.2.5 Outcomes

The primary outcomes included parameters related to metabolic and hepatic function:

- Lipid metabolism-related indicators encompassed triglycerides (TG), total cholesterol (TC), high-density lipoprotein cholesterol (HDL-C), and low-density lipoprotein cholesterol (LDL-C).
- Glycometabolism-related indicators included fasting blood glucose (FBG), glycated hemoglobin (HbA1c), insulin, and insulin resistance (HOMA-IR).
- Liver function-related markers covered alanine aminotransferase (ALT), aspartate aminotransferase (AST), alkaline phosphatase (ALP), and gamma-glutamyl transferase (GGT).
- Inflammatory cytokines comprised interleukin-6 (IL-6) and tumor necrosis factor-alpha (TNF-α).
- Oxidative stress-related indicators included total antioxidant capacity (TAC), superoxide dismutase (SOD), and MDA.

These indicators were selected based on their pathophysiological and clinical relevance in MAFLD. TG, TC, HDL-C, and LDL-C reflect the synthesis of hepatic lipids, oxidation, and lipoprotein transport. FBG, HbA1c, insulin and HOMA-IR reflect glucose metabolism and insulin sensitivity. ALT, AST, ALP, and GGT are common biochemical markers of hepatocellular injury and liver dysfunction. IL-6 and TNF-α, as key proinflammatory cytokines, reflect systemic inflammatory responses. TAC, SOD, and MDA represent oxidative stress and antioxidant balance, serving as important indicators of oxidative damage. These parameters have been widely used in clinical and translational studies on MAFLD and can comprehensively capture pathological changes in metabolism, inflammation, and oxidative stress. To ensure the comparability and transitivity required for NMA, all included RCTs enrolled adult MAFLD or NAFLD patients with similar baseline characteristics, used placebo or standard treatment as controls, and assessed consistent outcomes (metabolism-, hepatic function-, and oxidative stress-related parameters).

#### 2.2.6 Exclusion criteria

The following studies were excluded:

1. Patients had other liver diseases (such as viral hepatitis, autoimmune hepatitis, alcoholic liver disease, or drug-induced liver injury) or severe systemic diseases (such as malignancy, cardiac or renal failure);
2. Non-randomized or uncontrolled study design;
3. Studies involving children or animal models;
4. Studies with no essential data (e.g., means and standard deviations) or available data on outcome indicators;
5. Retrospective studies, observational studies, case series, conference abstracts, animal experiments, or reviews.

### 2.3 Data extraction and quality assessment

Two researchers (Y.Y.Y. and H.X.L.) independently conducted literature screening and data extraction. Any discrepancies were resolved through discussion with a third researcher (H.Y.M.). Duplicate records were removed using EndNote 20. Studies were preliminarily screened based on titles and abstracts, and then the full texts were reviewed to determine eligible studies.

For each included study, the following key information was extracted:

1. Study characteristics, including the first author, year of publication, country or region, sample size, study design, and intervention duration;
2. Intervention details, including the name of the dietary supplement or pharmacological agent, dosage, route of administration, duration of treatment, and whether combination therapy was used;
3. Interventions used in the control group, such as placebo, standard therapy, or other active interventions.

Additionally, data were extracted for all predefined primary outcomes, including indicators related to metabolism, hepatic function, inflammation, and oxidative stress, as described above.

When data were presented only in a graphical form, numerical values were extracted using GetData Graph Digitizer 2.26 and standardized according to the conversion formulas recommended in the *Cochrane Handbook* (version 6.3). If only medians and interquartile ranges were reported, means and standard deviations were estimated using established conversion equations to ensure the comparability and consistency of data.

The methodological quality of the included studies was independently evaluated by two researchers using the Cochrane Risk of Bias 2.0 (RoB 2.0) tool. This tool encompasses five domains, including random sequence generation, allocation concealment, blinding of participants and personnel, outcome assessment, and completeness and reporting of outcome data. Each study was rated as having a low, unclear, or high risk of bias. Disagreements, if any, were resolved by a third reviewer. The assessment results were visualized using a risk of bias plot and a summary plot.

### 2.4 Statistical analysis

All statistical analyses were conducted using R software (version 4.4.1) within a Bayesian framework. Model fitting and inference were performed with the gemtc and rjags packages, while netmeta was used solely to visualize network structure (Supplementary Material S1). Effect estimates were expressed as mean differences (MDs) with 95% credible intervals (CrIs). Nnetworks of intervention were constructed using the mtc.network () function, and network diagrams were generated using the plot (network) function, where node size was proportional to sample size, and edge width reflected the number of studies providing direct comparisons. A Bayesian random-effects model was then established under the assumption of consistency, with four Markov chains (n.chain = 4), a burn-in period (n.adapt) of 5,000 iterations, 20,000 sampling iterations (n.iter), and a thinning interval (thin) of 1. Parameter estimation was conducted using the Markov Chain Monte Carlo (MCMC) method. If low heterogeneity was found (I^2^ < 50%), a fixed-effects model was used. Model convergence was evaluated using the Gelman–Rubin diagnostic and visualized through trace and density plots generated by Gelman plot. A potential scale reduction factor (PSRF) close to 1.00 indicated satisfactory convergence. Forest plots were drawn. To ensure the comparability and interpretive consistency of effect measures, all continuous outcomes were standardized to uniform units (for example, mmol/L, U/L, μmol/L). To comprehensively evaluate the relative efficacy of interventions, the surface under the cumulative ranking curve (SUCRA) values were calculated. The SUCRA value ranged from 1% to 100%. A higher SUCRA value closer to 100% indicated a greater likelihood of the intervention ranking highest or among the highest. Additionally, a league table was generated to systematically compare the relative effects among different treatment options. Consistency was evaluated using the node-splitting method, and a P-value greater than 0.05 indicated no significant inconsistency between direct and indirect comparisons. When substantial heterogeneity was observed (I^2^ > 50% or a marked increase in τ^2^), a random-effects model was used. Sensitivity analysis was performed to explore the source of heterogeneity.

## 3 Results

### 3.1 Basic characteristics of the included studies

**FIGURE 1:**
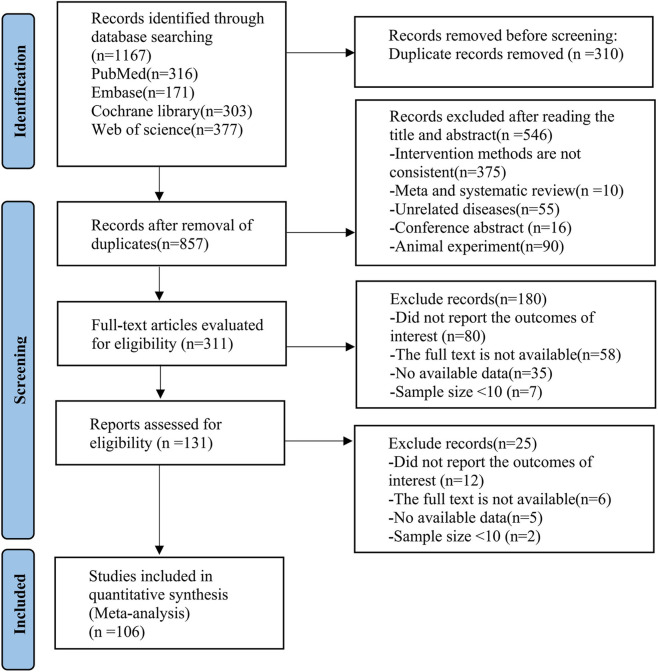
Literature screening process.

A total of 1,167 relevant publications were identified from PubMed, Embase, Web of Science, and Cochrane Library databases. After removing 310 duplicate records, the titles and abstracts of the remaining studies were screened. Two investigators independently excluded 546 studies that did not meet the eligibility criteria. Then, the full texts of 311 studies were reviewed. Subsequently, 180 ineligible studies were excluded. An additional 25 studies were excluded due to insufficient or inaccessible data on outcomes. Ultimately, 106 RCTs were included in the NMA (Celinski et al., 2014; Chachay et al., 2014; Choi et al., 2014; Faghihzadeh et al., 2014; Farhangi et al., 2014; Han et al., 2014; Mohammadshahi et al., 2014; Sharifi et al., 2014; Solhi et al., 2014; Aller et al., 2015; Amiri-Moghadam et al., 2015; Chen et al., 2015; El-Haggar and Mostafa, 2015; Faghihzadeh et al., 2015; Guy et al., 2015; Sorrentino et al., 2015; Ebrahimi-Mameghani et al., 2016; Ekhlasi et al., 2016; Federico et al., 2017; Heebøll et al., 2016; Hong et al., 2016; Panahi et al., 2016; Rahimlou et al., 2016; Rahmani et al., 2016; Rangboo et al., 2016; Ebrahimi-Mameghani et al., 2017; Ghaffari et al., 2017; Navekar et al., 2017; Pakravan et al., 2017; Pakzad et al., 2017; Panahi et al., 2017; Polyzos et al., 2017; Zöhrer et al., 2017; Amanat et al., 2018; Amirkhizi et al., 2018; Asghari et al., 2018; Ghaffari et al., 2018; Kantartzis et al., 2018; Hosseinpour-Arjmand et al., 2019; Pervez et al., 2018; Saadati et al., 2018; Anushiravani et al., 2019; Bril et al., 2019; Chashmniam et al., 2019; Cheraghpour et al., 2019; Jazayeri-Tehrani et al., 2019; Mirhafez et al., 2019; Nobili et al., 2019; Oliveira et al., 2019; Panahi et al., 2019; Rahmanabadi et al., 2019; Rashidmayvan et al., 2019; Saadati et al., 2019a; Saadati et al., 2019b; Torquato et al., 2019; Bahrami et al., 2020; Cerletti et al., 2020; Farzin et al., 2020; Fathi et al., 2020a; Fathi et al., 2020b; Ferro Y. et al., 2020; Hariri et al., 2020; Moradi Kelardeh et al., 2020; Pervez et al., 2020; Pour et al., 2020; Rafie et al., 2020; Saberi-Karimian et al., 2020; Sangouni et al., 2020; Amerikanou et al., 2021; Darvish et al., 2021; Domech et al., 2021; Fouda et al., 2021; Harrison et al., 2021; He et al., 2021; Jarhahzadeh et al., 2021; Kedarisetty et al., 2021; Kooshki et al., 2021; Majnooni et al., 2021; Mirhafez et al., 2021; Namkhah et al., 2021; Yari et al., 2021; Akbari et al., 2022; Arefhosseini et al., 2022; Atarodi et al., 2022; Ferro et al., 2022; Hosseinian et al., 2022; Kalhori et al., 2022; Mohammadi et al., 2022; Naeini et al., 2022; Pervez et al., 2022; Rashidmayvan et al., 2022; Rostamizadeh et al., 2022; Wang et al., 2022; Xie et al., 2022; Zojaji et al., 2022; Beheshti Namdar et al., 2023; Damavandi et al., 2023; Ghoreishi et al., 2024; Khoshbaten et al., 2023; Majeed et al., 2023; Mojtahedi et al., 2023; Rezaei et al., 2023; Safari et al., 2023; Sharifi et al., 2023; Tutunchi et al., 2023a; Tutunchi et al., 2023b). The detailed study selection process is illustrated in Figure 1.

The included studies were published between 2014 and 3 March 2024. Among these studies, 84 were conducted in Asia, 14 in Europe, 3 in the Americas (including 3 in the United States, 1 in North America, and 1 in South America), 2 in Africa, and 1 in Australia. In total, 7,273 participants were included, of whom 2.87% had NASH and 97.13% had MAFLD. The intervention duration across studies ranged from 6 weeks to 14 months. The main study characteristics and baseline information of participants are summarized in Supplementary Table S1.

All 106 included studies were RCTs evaluating the efficacy of dietary supplements or pharmacological agents in patients with MAFLD. Based on the nature and mechanisms of the interventions, treatments were categorized into the following groups: vitamins and minerals (including vitamin E, vitamin D, vitamin C, and zinc supplementation); bioactive metabolic regulators (such as α-lipoic acid, melatonin, and myo-inositol); phytochemicals (including genistein, resveratrol, and curcumin); herbal extracts (such as garlic, ginger, and purslane extract); polyherbal formulas (traditional preparations containing multiple plant-derived components); pharmacological agents (including metformin, thiazolidinediones [TZDs], lipid-lowering agents, hepatoprotective agents, vasodilators, and endogenous lipid regulators such as oleoylethanolamide [OEA]); and low-calorie diets, as part of lifestyle modification interventions. To explore synergistic effects of multi-mechanistic strategies, several studies adopted combination interventions, mainly including nutrient + pharmacological intervention (e.g., pioglitazone + vitamin E, metformin + vitamin E, pentoxifylline + vitamin E), phytochemical + pharmacological intervention (e.g., metformin + artichoke leaf extract), nutrient + phytochemical (e.g., vitamin E + artichoke leaf extract), pharmacological intervention + bioactive regulator (e.g., metformin + N-acetylcysteine), pharmacological intervention + pharmacological intervention (e.g., metformin + ursodeoxycholic acid [UDCA]), and phytochemical + herbal extracts (e.g., curcumin + licorice root extract).

In this study, the network of interventions was structured with placebo as the reference node. Each node represented a specific intervention. The size of nodes was proportional to the sample size. The size of lines indicated the number and strength of direct comparisons. All treatment strategies were encoded and categorized according to their mechanisms of action as follows: A, vitamins and minerals; B, bioactive metabolic regulators; C, low-calorie diet; D, phytochemicals; E, herbal extracts; F, polyherbal formulas; G, metformin; H, TZDs; I, lipid-lowering agents; J, hepatoprotective agents; K, vasodilators; L, endogenous lipid regulator (OEA); M, nutrient + pharmacological intervention; N, phytochemical + pharmacological intervention; O, nutrient + phytochemical; P, pharmacological intervention + bioactive regulator; Q, pharmacological intervention + pharmacological; R, phytochemical + herbal extract. Detailed classifications are presented in Supplementary Table S2.

### 3.2 Quality assessment of included studies

**FIGURE 2:**
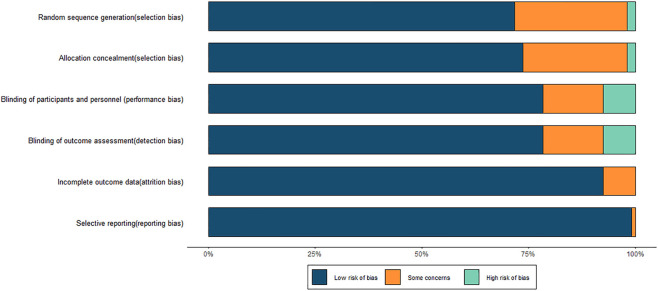
Risk of bias graph.

The methodological quality of the 106 RCTs was assessed. Among them, 76 studies adequately reported the generation of a random sequence and were rated as having a low risk of bias. Two studies were assessed as having a high risk of bias due to incomplete descriptions or insufficient allocation concealment. A total of 83 studies reported the use of double-blind or single-blind designs, indicating a low risk of bias in the blinding of participants and investigators. Regarding outcome reporting, 99 studies reported both the prespecified primary and secondary outcomes, without evident data omissions or selective reporting, and were thus classified as having a low risk of bias. A few remaining studies were rated as having some concerns due to incomplete information on outcomes. Furthermore, 105 studies presented their results in accordance with their prespecified protocols, suggesting a generally reliable reporting quality. However, several studies did not provide sufficient details on randomization and allocation concealment, and whether blinding was implemented was unclear in a few studies. These factors indicated that some degree of methodological bias cannot be entirely ruled out. Overall, the quality of the included studies was acceptable. The results of the risk of bias assessment are shown in Figure 2, and detailed evaluations are provided in Supplementary Figure S1; Supplementary Table S3.

### 3.3 NMA

#### 3.3.1 Lipid metabolism indicators

**FIGURE 3:**
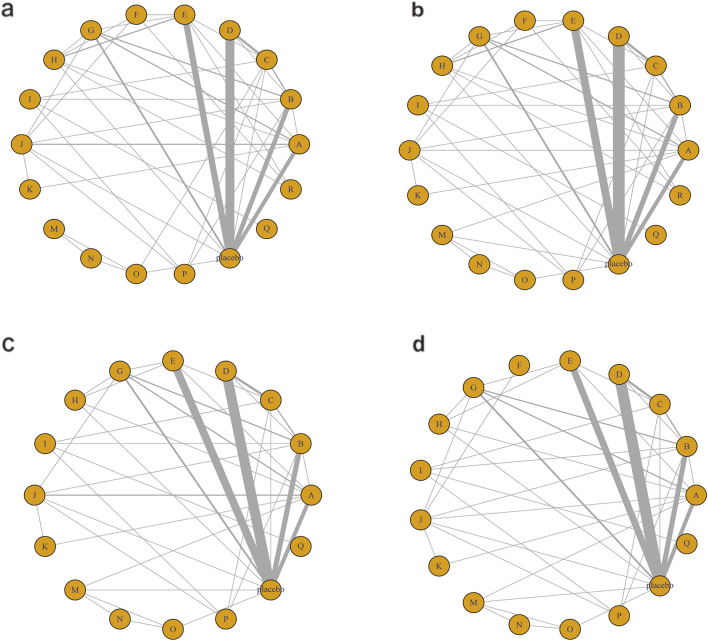
Network mapping diagram of lipid metabolism. **(a)** TG, **(b)** TC, **(c)** HDL-C, **(d)** LDL-C.

**FIGURE 4:**
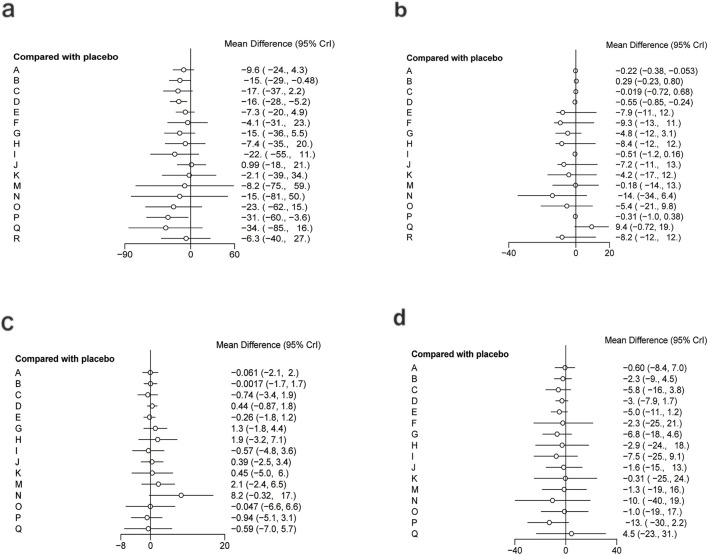
Forest plot of lipid metabolism. **(a)** TG, **(b)** TC, **(c)** HDL-C, **(d)** LDL-C.

The network diagram for the relationships among different interventions for lipid metabolism-related indicators (such as TG, TC, HDL-C, and LDL-C) is illustrated in Figures 3a–d. A line indicates direct comparison of interventions, and the width of each solid line represents the number of studies. Figure 4 presents the direct comparison results between each intervention and placebo.

**FIGURE 5:**
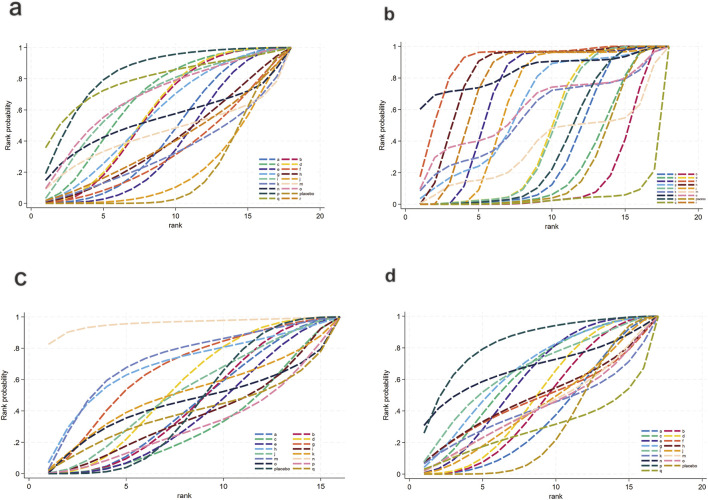
SUCRA for lipid metabolism. **(a)** TG, **(b)** TC, **(c)** HDL-C, **(d)** LDL-C. Different colored curves represent different interventions, and the area under each curve corresponds to its cumulative probability value (SUCRA). SUCRA values range from 1% to 100%. The closer the SUCRA value is to 100%, the more likely the intervention is to rank highest or among the top.

For TG (Figure 3a), a total of 4,145 participants and 18 interventions were included. The results showed that pharmacological intervention + bioactive regulator (MD: −31.0; 95% CrI: −60.0 to −3.6) and pharmacological intervention + another pharmacological intervention (MD: −34.0; 95% CrI: −85.0 to 16.0) were the most effective in reducing TG, followed by nutrient + phytochemical (MD: −23.0; 95% CrI: −62.0 to 15.0) (Figure 4a). According to SUCRA rankings (Figure 5a), pharmacological intervention + bioactive regulator ranked first (SUCRA = 0.83) in reducing TG. Further analysis revealed that within this combination category, Essentiale Forte + tryptophan (MD: −70.0; 95% CrI: −120.0 to −24.0) had the most significant effect on lowering TG, followed by Abexol + atorvastatin (MD: −16.0; 95% CrI: −48.0 to −15.0). Overall, pharmacological intervention + another pharmacological intervention (SUCRA = 0.78) and nutrient + phytochemical (SUCRA = 0.68) ranked second and third, respectively, while placebo ranked the lowest (SUCRA = 0.20). The node-splitting analysis diagram and heterogeneity forest plot for TG are shown in Supplementary Figures S2, S3, and the league table is presented in Supplementary Table S4.

For TC (Figure 3b), 4,469 participants and 18 interventions were included. Compared with placebo, phytochemicals (MD: −0.55; 95% CrI: −0.85 to −0.24) significantly reduced TC levels, followed by vitamins and minerals (MD: −0.22; 95% CrI: −0.38 to −0.053) (Figure 4b). However, SUCRA rankings (Figure 5b) indicated that polyherbal formulas (SUCRA = 0.88), phytochemical + pharmacological intervention (SUCRA = 0.84), and TZDs (SUCRA = 0.80) had the highest probabilities of being the most effective interventions. This discrepancy may be due to the integration of both direct and indirect comparisons in SUCRA ranking, providing more stable overall estimates. The node-splitting analysis diagram and heterogeneity forest plot for TC are shown in Supplementary Figures S4, S5, and the league table is presented in Supplementary Table S5.

For HDL-C (Figure 3c), 4,170 participants and 16 interventions were analyzed. Compared with placebo, phytochemical + pharmacological intervention (metformin + artichoke leaf extract) was the most efficacious in increasing HDL-C (MD: 8.2; 95% CrI: −0.32–17.0), followed by nutrient + pharmacological intervention (MD: 2.1; 95% CrI: −2.4–6.5) and TZDs (MD: 1.9; 95% CrI: −3.2–7.1) (Figure 4c). SUCRA rankings (Figure 5c) indicated that phytochemical + pharmacological intervention (SUCRA = 0.96) was most effective in elevating HDL-C levels, followed by nutrient + pharmacological intervention (SUCRA = 0.72) and TZDs (SUCRA = 0.68). The node-splitting analysis diagram and heterogeneity forest plot for HDL-C are shown in Supplementary Figures S6, S7, and the league table is presented in Supplementary Table S6.

For LDL-C (Figure 3d), 4,008 participants and 17 interventions were included. Compared with placebo, pharmacological intervention + bioactive regulator (MD: −13.0; 95% CrI: −30.0 to 2.2) significantly reduced LDL-C, followed by phytochemical + pharmacological intervention (MD: −10.0; 95% CrI: −40.0 to 19.0) and lipid-lowering agents (MD: −7.5; 95% CrI: −25.0 to 9.1) (Figure 4d). According to SUCRA rankings (Figure 5d), pharmacological intervention + bioactive regulator ranked first (SUCRA = 0.83). Further analysis revealed that within this category, Essentiale Forte + tryptophan was the most effective in reducing LDL-C (MD: −62.0; 95% CrI: −99.0 to −25.0), followed by Abexol + atorvastatin (MD: −37.0; 95% CrI: −99.0 to −25.0). Phytochemical + pharmacological intervention (SUCRA = 0.67) and lipid-lowering agents (SUCRA = 0.65) ranked higher. The node-splitting analysis diagram and heterogeneity forest plot for LDL-C are shown in Supplementary Figures S8, S9, and the league table is presented in Supplementary Table S7.

#### 3.3.2 Glycometabolism-related indicators

**FIGURE 6:**
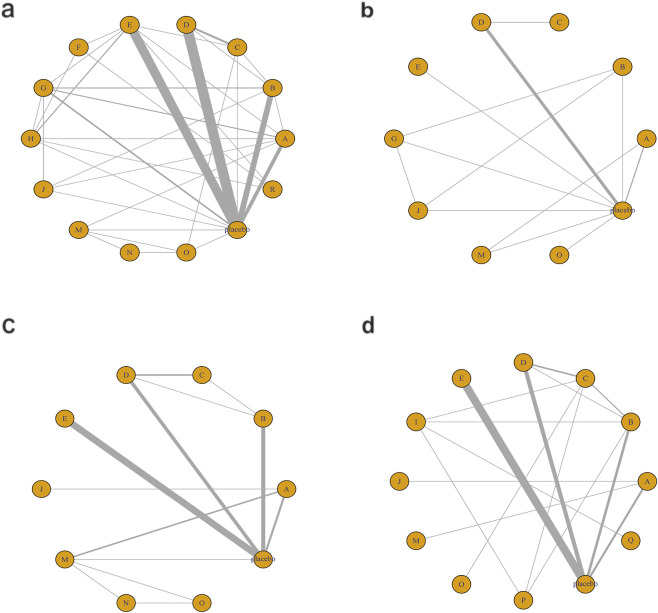
Network mapping diagram of glycometabolism. **(a)** FBG, **(b)** HbA1c, **(c)** Insulin, **(d)** HOMA-IR.

Figure 6 illustrates the network relationships among different interventions and placebo in improving glycometabolism-related indicators, including FBG, HbA1c, insulin, and HOMA-IR.

**FIGURE 7:**
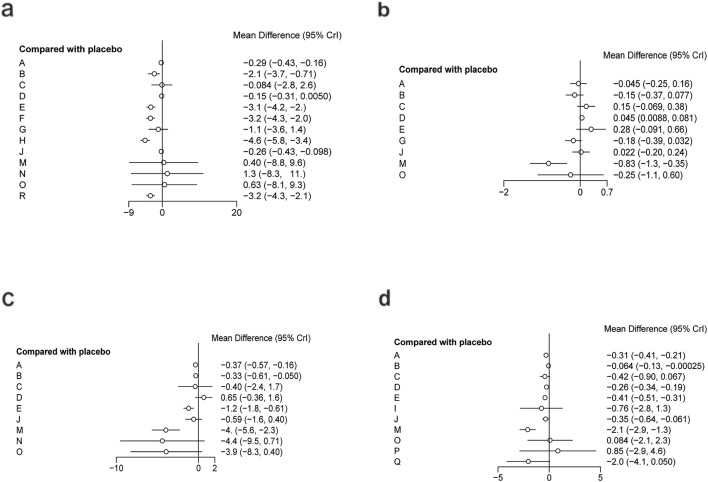
Forest plot of glycometabolism. **(a)** FBG, **(b)** HbA1c, **(c)** Insulin, **(d)** HOMA-IR.

**FIGURE 8:**
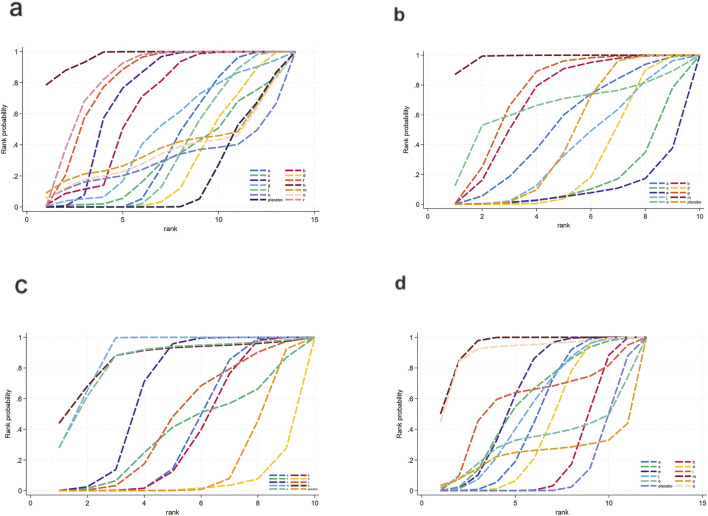
SUCRA for glycometabolism. **(a)** FBG, **(b)** HbA1c, **(c)** Insulin, **(d)** HOMA-IR. Different colored curves represent different interventions, and the area under each curve corresponds to its cumulative probability value (SUCRA). SUCRA values range from 1% to 100%. The closer the SUCRA value is to 100%, the more likely the intervention is to rank highest or among the top.

For FBG (Figure 6a), a total of 4,421 participants and 14 treatment regimens were included. Compared with placebo, TZDs (MD: −4.6; 95% CrI: −5.8 to −3.4), phytochemical + herbal extract (MD: −3.2; 95% CrI: −4.3 to −2.1), polyherbal formulas (MD: −3.2; 95% CrI: −4.3 to −2.0), and herbal extracts (MD: −3.1; 95% CrI: −4.2 to −2.0) all significantly reduced FBG (Figure 7a). According to SUCRA rankings (Figure 8a), TZDs (SUCRA = 0.97), phytochemical + herbal extract (SUCRA = 0.83), polyherbal formulas (SUCRA = 0.80), and herbal extracts (SUCRA = 0.71) ranked higher in lowering FBG, while placebo ranked the lowest (SUCRA = 0.19). The node-splitting analysis diagram and heterogeneity forest plot for FBG are shown in Supplementary Figures S10, S11, and the league table is presented in Supplementary Table S8.

For HbA1c (Figure 6b), a total of 954 participants and 10 treatment regimens were included. Compared with placebo, nutrient + pharmacological intervention (pioglitazone + vitamin E) (MD: −0.83; 95% CrI: −1.30 to −0.35) significantly reduced HbA1c (Figure 7b). SUCRA rankings (Figure 8b) indicated that nutrient + pharmacological intervention (SUCRA = 0.98) had the best overall efficacy. Although the results of metformin (MD: −0.18; 95% CrI: −0.39 to 0.032) and bioactive metabolic regulators (MD: −0.15; 95% CrI: −0.37 to 0.077) did not reach statistical significance, both showed a decreasing trend in HbA1c, ranking second (SUCRA = 0.75) and third (SUCRA = 0.70), respectively. Despite a limited number of comparative studies, no significant inconsistency was observed. The heterogeneity forest plot for HbA1c is shown in Supplementary Figure S12, and the league table is presented in Supplementary Table S9.

For insulin (Figure 6c), 2,546 participants and 10 treatment regimens were included. Compared with placebo, nutrient + pharmacological intervention (MD: −4.0; 95% CrI: −5.6 to −2.3) was the most effective in reducing insulin, followed by phytochemical + pharmacological intervention (MD: −4.4; 95% CrI: −9.5 to 0.71) and nutrient + phytochemical (−3.9; 95% CrI: −8.3 to 0.40). In addition, herbal extracts (MD: −1.2; 95% CrI: −1.8 to −0.61) also moderately reduced insulin (Figure 7c). SUCRA results (Figure 8c) indicated that nutrient + pharmacological intervention (SUCRA = 0.88) ranked first. Further analysis revealed that pentoxifylline + vitamin E was the most effective in reducing insulin (MD: −4.1; 95% CrI: −7. to 0.57), followed by pioglitazone + vitamin E (MD: −1.4; 95% CrI: −4.0 to 1.3). Overall, phytochemical + pharmacological intervention (SUCRA = 0.85) and nutrient + phytochemical (SUCRA = 0.83) ranked second and third in lowering fasting insulin levels. The node-splitting analysis diagram and heterogeneity forest plot for Insulin are shown in Supplementary Figures S13, S14, and the league table is presented in Supplementary Table S10.

For HOMA-IR (Figure 6d), 2,592 participants and 12 treatment regimens were included. Compared with placebo, nutrient + pharmacological intervention (spironolactone + vitamin E) (MD: −2.1; 95% CrI: −2.9 to −1.3) and pharmacological intervention + pharmacological intervention (fenofibrate + pentoxifylline) (MD: −2.0; 95% CrI: −4.1 to 0.050) was the most efficacious in reducing HOMA-IR, followed by herbal extracts (MD: −0.41; 95% CrI: −0.51 to −0.31). Furthermore, vitamins and minerals (MD: −0.31; 95% CrI: −0.41 to −0.21) and phytochemicals (MD: −0.26; 95% CrI: −0.34 to −0.19) mildly but consistently reduced HOMA-IR (Figure 7d). SUCRA rankings (Figure 8d) demonstrated that nutrient + pharmacological intervention (SUCRA = 0.94) and pharmacological intervention + pharmacological intervention (SUCRA = 0.91) had the best overall efficacy, followed by herbal extracts (SUCRA = 0.63). The node-splitting analysis diagram and heterogeneity forest plot for HOMA-IR are shown in Supplementary Figures S15, S16, and the league table is presented in Supplementary Table S11.

#### 3.3.3 Liver injury-related biomarkers

**FIGURE 9:**
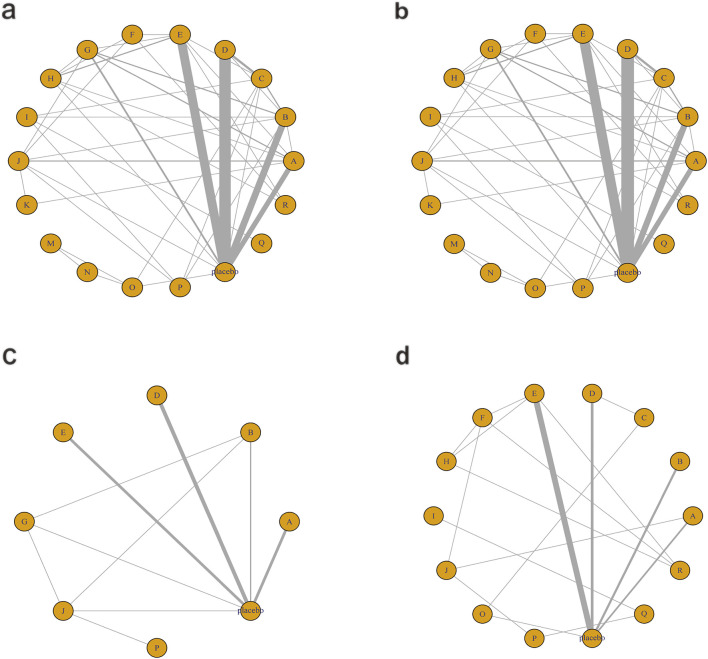
Network mapping diagram of liver injury markers. **(a)** ALT, **(b)** AST, **(c)** ALP, **(d)** GGT.

Figure 9 illustrates the network relationships among different interventions and placebo in improving liver injury-related biomarkers such as ALT, AST, ALP, and GGT.

**FIGURE 10:**
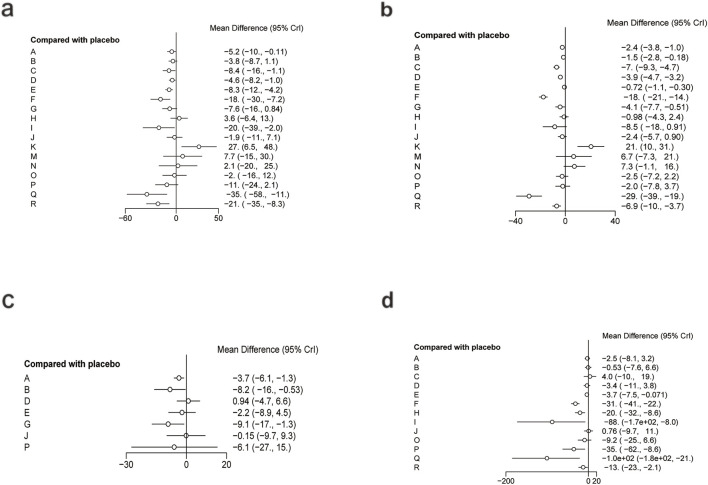
Forest plot of liver injury markers. **(a)** ALT, **(b)** AST, **(c)** ALP, **(d)** GGT.

**FIGURE 11:**
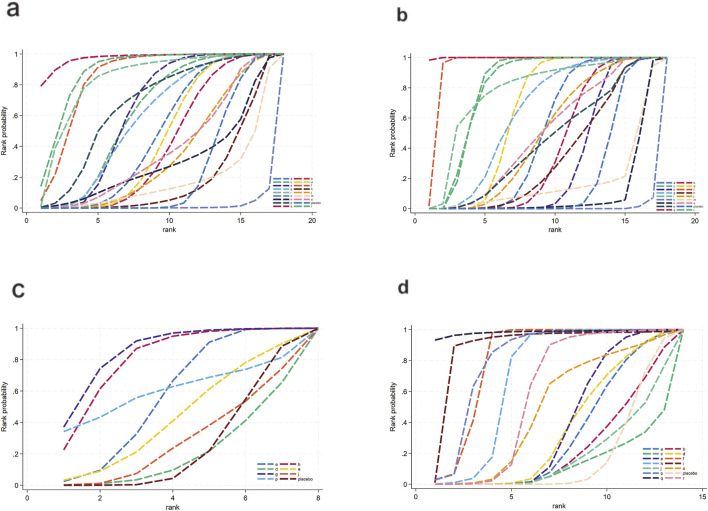
SUCRA for liver injury markers. **(a)** ALT, **(b)** AST, **(c)** ALP, **(d)** GGT. Different colored curves represent different interventions, and the area under each curve corresponds to its cumulative probability value (SUCRA). SUCRA values range from 1% to 100%. The closer the SUCRA value is to 100%, the more likely the intervention is to rank highest or among the top.

For ALT, a total of 5,006 participants and 18 treatment regimens were included (Figure 9a). Compared with placebo, pharmacological intervention + pharmacological intervention (fenofibrate + pentoxifylline) (MD: −35.0; 95% CrI: −58.0 to −11.0) was the most efficacious in reducing ALT, followed by phytochemical + herbal extract (MD: −21.0; 95% CrI: −35.0 to −8.3), polyherbal formulas (MD: −18.0; 95% CrI: −30.0 to −7.2), and lipid-lowering agents (MD: −20.0; 95% CrI: −39.0 to −2.0) (Figure 10a). In addition, herbal extracts (MD: −8.3; 95% CrI: −12.0 to −4.2), low-calorie diet (MD: −8.4; 95% CrI: −16.0 to −1.1), and phytochemicals (MD: −4.6; 95% CrI: −8.2 to −1.0) also moderately reduced ALT. According to SUCRA rankings (Figure 11a), pharmacological intervention + pharmacological intervention (SUCRA = 0.97), phytochemical + herbal extract (SUCRA = 0.88), and polyherbal formulas (SUCRA = 0.85) ranked high. The node-splitting analysis diagram and heterogeneity forest plot for ALT are shown in Supplementary Figures S17, S18, and the league table is presented in Supplementary Table S12.

For AST, a total of 5,177 participants and 18 treatment regimens were included (Figure 9b). Compared with placebo, pharmacological intervention + pharmacological intervention (fenofibrate + pentoxifylline) (MD: −29.0; 95% CrI: −39.0 to −19.0) was the most effective in reducing AST, followed by polyherbal formulas (MD: −18.0; 95% CrI: −21.0 to −14.0), lipid-lowering agents (MD: −8.5; 95% CrI: −18.0 to 0.91), and low-calorie diet (MD: −7.0; 95% CrI: −9.3 to −4.7). Moreover, phytochemical + herbal extract (MD: −6.9; 95% CrI: −10.0 to −3.7) and metformin (MD: −4.1; 95% CrI: −7.7 to −0.51) also moderately reduced AST (Figure 10b). According to SUCRA rankings (Figure 11b), nutrient + phytochemical (SUCRA = 0.99), polyherbal formulas (SUCRA = 0.94), and low-calorie diet (SUCRA = 0.80) ranked high. The node-splitting analysis diagram and heterogeneity forest plot for ALT are shown in Supplementary Figures S19, S20, and the league table is presented in Supplementary Table S13.

For ALP, 1,267 participants and 8 treatment regimens were included (Figure 9c). Compared with placebo, metformin (MD: −9.1; 95% CrI: −17.0 to −1.3) and bioactive metabolic regulators (MD: −8.2; 95% CrI: −16.0 to −0.53) significantly reduced ALP (Figure 10c). Additionally, vitamins and minerals (MD: −3.7; 95% CrI: −6.1 to −1.3) exhibited a mild-to-moderate effect. Based on SUCRA rankings (Figure 11c), metformin (SUCRA = 0.85), bioactive metabolic regulators (SUCRA = 0.80), and vitamins and minerals (SUCRA = 0.57) ranked high in improving ALP. No significant inconsistency was observed among studies. The heterogeneity forest plot for ALP is shown in Supplementary Figure S21, and the league table is presented in Supplementary Table S14.

For GGT (Figure 9d), 2,485 participants and 14 treatment regimens were included. Compared with placebo, pharmacological intervention + pharmacological intervention (metformin + UDCA) (MD: −100.0; 95% CrI: −180.0 to −21.0) significantly reduced GGT, followed by lipid-lowering agents (MD: −88.0; 95% CrI: −170.0 to −8.0) and pharmacological intervention + bioactive regulator (MD: −35.0; 95% CrI: −62.0 to −8.6) (Figure 10d). Moreover, polyherbal formulas (MD: −31.0; 95% CrI: −41.0 to −22.0) and TZDs (MD: −20.0; 95% CrI: −32.0 to −8.6) also showed significant effects (Figure 10d). According to SUCRA rankings (Figure 11d), pharmacological intervention + pharmacological intervention (SUCRA = 0.98), lipid-lowering agents (SUCRA = 0.89), and pharmacological intervention + bioactive regulator (SUCRA = 0.80) ranked high in improving GGT. No significant inconsistency or high heterogeneity was detected among studies. The node-splitting analysis diagram and heterogeneity forest plot for GGT are shown in Supplementary Figures S22, S23, and the league table is presented in Supplementary Table S15.

#### 3.3.4 Inflammation and oxidative stress-related indicators

**FIGURE 12:**
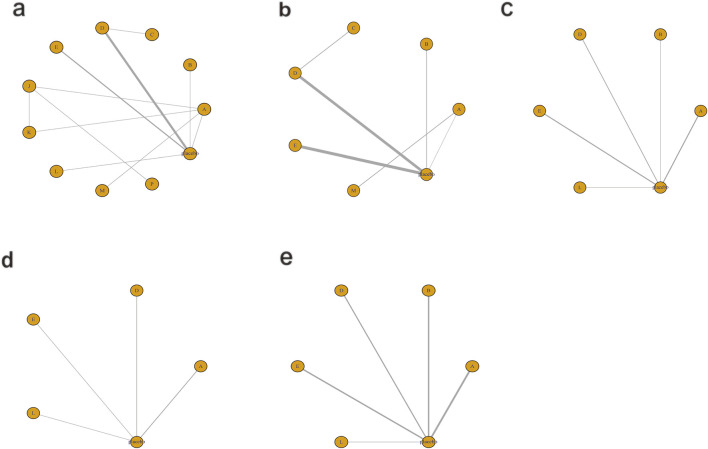
Network mapping diagram of inflammatory and oxidative stress indicators. **(a)** IL-6, **(b)** TNF-α, **(c)** TAC, **(d)** SOD, **(e)** MDA.

Figure 12 illustrates the network relationships among different interventions and placebo in improving inflammation- and oxidative stress-related indicators.

##### 3.3.4.1 Inflammatory markers

**FIGURE 13:**
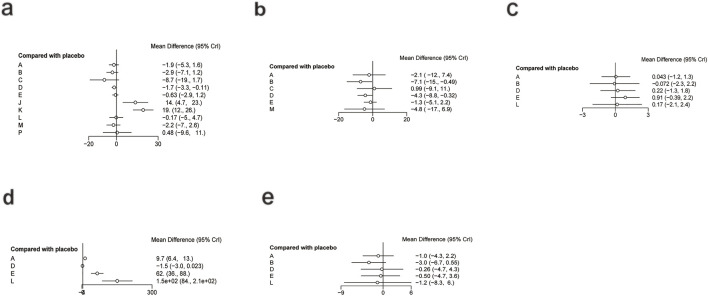
Forest plot of inflammatory and oxidative stress indicators. **(a)** IL-6, **(b)** TNF-α, **(c)** TAC, **(d)** SOD, **(e)** MDA.

**FIGURE 14:**
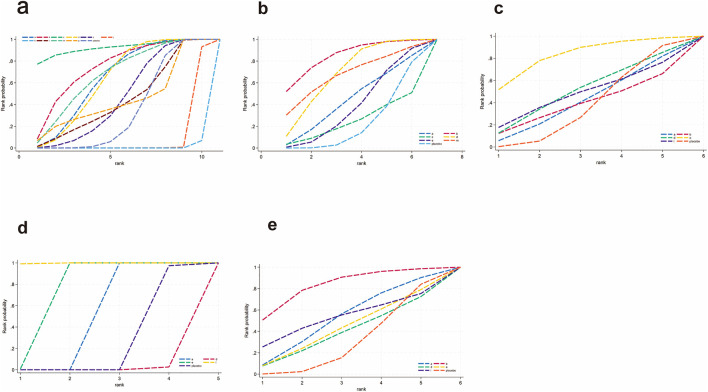
SUCRA for inflammatory and oxidative stress indicators. **(a)** IL-6, **(b)** TNF-α, **(c)** TAC, **(d)** SOD, **(e)** MDA. Different colored curves represent different interventions, and the area under each curve corresponds to its cumulative probability value (SUCRA). SUCRA values range from 1% to 100%. The closer the SUCRA value is to 100%, the more likely the intervention is to rank highest or among the top.

For IL-6 (Figure 12a), a total of 1,043 participants and 11 intervention types were included. Compared with placebo, phytochemicals (MD: −1.7; 95% CrI: −3.3 to −0.11) significantly reduced IL-6, indicating a notable anti-inflammatory effect (Figure 13a). However, SUCRA rankings (Figure 14a) revealed that low-calorie diet (SUCRA = 0.92), bioactive metabolic regulators (SUCRA = 0.75), and nutrient + pharmacological intervention (SUCRA = 0.67) exhibited more stable overall efficacy across multiple comparisons. This discrepancy may arise from differences in the weight of direct versus network-based evidence. Phytochemicals showed significant effects in direct comparisons with placebo. In contrast, low-calorie diet, bioactive metabolic regulators, and nutrient + pharmacological intervention showed more consistent results across indirect comparisons, leading to higher SUCRA rankings. No significant inconsistency was detected among studies. The heterogeneity forest plot for IL-6 is shown in Supplementary Figure S24, and the league table is presented in Supplementary Table S16.

For TNF-α (Figure 12b), a total of 1,359 participants and 7 intervention types were included. Compared with placebo, bioactive metabolic regulators (MD: −7.1; 95% CrI: −15.0 to −0.49) significantly reduced TNF-α, followed by phytochemicals (MD: −4.3; 95% CrI: −8.8 to −0.32) (Figure 13b). SUCRA rankings (Figure 14b) indicated that bioactive metabolic regulators (SUCRA = 0.84) ranked first. Further analysis revealed that L-carnitine within this category had the most pronounced effect in reducing TNF-α (MD: −19; 95% CrI: −26 to −12). Phytochemicals (SUCRA = 0.69) and nutrient + pharmacological intervention (SUCRA = 0.67) ranked second and third, respectively. No significant inconsistency was observed among studies. The heterogeneity forest plot for TNF-α is shown in Supplementary Figure S25, and the league table is presented in Supplementary Table S17.

##### 3.3.4.2 Oxidative stress-related markers

For TAC (Figure 12c), 516 participants and 6 types of interventions were included. Compared with placebo, herbal extracts (MD: 0.91; 95% CrI: −0.39–2.2) significantly increased TAC, suggesting a potential effect in enhancing systemic antioxidant capacity (Figure 13c). Phytochemicals (MD: 0.22; 95% CrI: −1.3–1.8) and OEA (MD: 0.17; 95% CrI: −2.1–2.4) also mildly increased TAC. According to SUCRA rankings (Figure 14c), herbal extracts (SUCRA = 0.83) demonstrated the best efficacy in improving TAC, followed by phytochemicals (SUCRA = 0.51) and OEA (SUCRA = 0.48). No significant inconsistency was identified among studies. The heterogeneity forest plot for TAC is shown in Supplementary Figure S26, and the league table is presented in Supplementary Table S18.

For SOD (Figure 12d), 313 participants and 5 intervention types were included. Compared with placebo, OEA (MD: 1.5 × 10^2^; 95% CrI: 84.0 to 2.1 × 10^2^) significantly increased SOD, followed by herbal extracts (MD: 62.0; 95% CrI: 36.0–88.0), suggesting a significant effect on improving antioxidant enzyme activity. Vitamins and minerals (MD: 9.7; 95% CrI: 6.4–13.0) showed a mild-to-moderate effect (Figure 13d). SUCRA rankings (Figure 14d) indicated that OEA (SUCRA = 0.97) had the best efficacy in elevating SOD levels, followed by herbal extracts (SUCRA = 0.81) and vitamins and minerals (SUCRA = 0.62). No significant inconsistency was observed across studies. The heterogeneity forest plot for SOD is shown in Supplementary Figure S27, and the league table is presented in Supplementary Table S19.

Finally, for MDA (Figure 12e), 1,007 participants and 6 intervention types were included. Compared with placebo, bioactive metabolic regulators (MD: −3.0; 95% CrI: −6.7 to 0.55) reduced MDA (Figure 13e). According to SUCRA rankings (Figure 14e), bioactive metabolic regulators (SUCRA = 0.83), OEA (SUCRA = 0.53), and vitamins and minerals (SUCRA = 0.52) ranked high in reducing MDA levels and mitigating oxidative damage. No significant inconsistency was detected among studies. The heterogeneity forest plot for MDA is shown in Supplementary Figure S28, and the league table is presented in Supplementary Table S20.

In summary, different interventions demonstrated distinct mechanisms in modulating oxidative stress. Herbal extracts exhibited strong effects in enhancing both TAC and SOD. Phytochemicals showed a mild effect on increasing TAC, while OEA demonstrated significant effects in increasing SOD levels and reducing lipid peroxidation. OEA, an endogenous lipid signaling molecule, can improve hepatic oxidative status by regulating fatty acid metabolism and redox balance (Sihag and Jones, 2018). Meanwhile, bioactive metabolic regulators showed significant efficacy in reducing MDA, suggesting that metabolic regulation and antioxidant mechanisms may act synergistically.

To further explore the relative differences in overall antioxidant effect, subgroup analysis was conducted by specific components from herbal extracts, phytochemicals, OEA, and bioactive metabolic regulators. The included interventions were as follows: (1) garlic; (2) ginger; (3) turmeric; (4) licorice root; (5) curcumin; (6) resveratrol; (7) genistein; (8) saffron; (9) oleoylethanolamide; (10) coenzyme Q10; (11) L-carnitine; and (12) α-lipoic acid.

Supplementary Figure S29 presents the effects of various interventions on TAC. The results indicated that garlic significantly increased TAC (MD: 2.3; 95% CrI: 2.2–2.4), suggesting a significant and consistent effect on improving total antioxidant capacity (Supplementary Figure S29a, b). In addition, saffron (MD: 0.35; 95% CrI: 0.049–0.65), OEA (MD: 0.16; 95% CrI: 0.080–0.24), and resveratrol (MD: 0.10; 95% CrI: 0.013–0.19) exhibited moderate effects. The SUCRA ranking (Supplementary Figure S29c) was as follows: garlic (SUCRA = 0.99) > saffron (SUCRA = 0.79) > OEA (SUCRA = 0.64) > resveratrol (SUCRA = 0.48) > ginger (SUCRA = 0.36) > placebo (SUCRA = 0.17) > coenzyme Q10 (SUCRA = 0.06). No significant inconsistency or high heterogeneity was detected among interventions. These results suggested that garlic may play a key role in enhancing systemic antioxidant status by promoting free radical scavenging and activating endogenous antioxidant systems.

Supplementary Figure S30 shows the effects of different interventions on MDA. L-carnitine significantly reduced MDA (MD: −12.0; 95% CrI: −13.0 to −10.0) (Supplementary Figure S30a, b), followed by OEA (MD: −1.2; 95% CrI: −1.6 to −0.77) and licorice root (MD: −0.98; 95% CrI: −1.4 to −0.58), with statistically significant differences. Additionally, garlic (MD: −0.30; 95% CrI: −0.43 to −0.17) and genistein (MD: −0.30; 95% CrI: −0.45 to −0.15) also exhibited mild-to-moderate effects. The SUCRA ranking (Supplementary Figure S30c) for the overall efficacy of interventions in reducing MDA levels was as follows: L-carnitine (SUCRA = 0.99) > OEA (SUCRA = 0.82) > licorice root (SUCRA = 0.75) > saffron (SUCRA = 0.61) > garlic (SUCRA = 0.46) > genistein (SUCRA = 0.45) > coenzyme Q10 (SUCRA = 0.43) > α-lipoic acid (SUCRA = 0.37) > curcumin (SUCRA = 0.31) > placebo (SUCRA = 0.17) > turmeric (SUCRA = 0.12). No significant inconsistency or high heterogeneity was observed among interventions. Overall, garlic and L-carnitine were the most effective in enhancing total antioxidant capacity and reducing lipid peroxidation, respectively, while OEA demonstrated consistent effects across both oxidative stress-related indicators. These results suggested its advantage in enhancing overall antioxidant effects and suppressing lipid peroxidation.

#### 3.3.5 Sensitivity analysis

To verify the robustness of the NMA results, a sensitivity analysis was conducted by excluding studies on combination intervention and retaining only studies on monotherapy. The results showed that the direction of effect estimates remained consistent with the overall analysis, and the SUCRA rankings were stable, indicating the robustness of the study conclusions (Supplementary Figures S31–S33).

## 4 Discussion

This study employed a Bayesian NMA to systematically compare the effects of various dietary supplements and pharmacological agents on metabolic regulation and oxidative stress in patients with MAFLD. The findings revealed heterogeneous patterns of action and potential synergistic mechanisms among different interventions across multiple outcomes. Traditional pairwise meta-analyses are generally limited to direct comparisons between two interventions, whereas studies on MAFLD treatment involve a wide variety of intervention types and complex mechanisms. Bayesian NMA can integrate both direct and indirect evidence to comprehensively evaluate the relative efficacy of multiple interventions, providing robust effect estimates (Cote et al., 2021; Watt et al., 2019). Therefore, this study utilized a Bayesian NMA approach to systematically compare diverse interventions across multidimensional outcomes, offering evidence-based guidance for the development of clinical treatment strategies.

Pathophysiologically, MAFLD begins with the accumulation of hepatic lipids (Ke et al., 2019). This accumulation is followed by excessive oxidative stress, along with the release of pro-inflammatory cytokines and adipokines, which collectively trigger hepatic inflammation, aggravate liver injury, and promote disease progression to more advanced stages (Day and James, 1998). Mitochondrial dysfunction plays a central role in this process by exacerbating oxidative stress and promoting lipid peroxidation (Mendoza et al., 2024). Together, mitochondrial impairment, lipid accumulation, and oxidative stress form a self-perpetuating vicious cycle that accelerates hepatic damage (Zheng et al., 2023). At the molecular level, alterations in gene expression related to lipid metabolism, oxidative enzymes, and mitochondrial biosynthesis further contribute to the progression of MAFLD. For instance, dysregulation of genes involved in fatty acid β-oxidation (e.g., PPAR-α and CPT1) and mitochondrial antioxidant defense systems (e.g., SOD and Nrf2) has been observed in MAFLD, impairing lipid metabolism and increasing oxidative stress (Yang et al., 2025; Ni et al., 2025; Xiao et al., 2024). These changes in gene expression exacerbate metabolic dysfunction and inflammatory responses, thereby promoting disease progression.

The present study found that pharmacological intervention + bioactive regulator (e.g., Essentiale Forte + tryptophan) exerted the most significant effects in reducing serum TG and LDL-C. Essentiale can improve lipid metabolic homeostasis by promoting phospholipid metabolism, repairing the integrity of hepatocellular membrane, and enhancing fatty acid β-oxidation (Chwiecko et al., 1993). Tryptophan, an essential amino acid, produces metabolites that modulate insulin signaling pathways and suppress inflammatory responses, thereby further supporting lipid metabolism (Deng et al., 2024). Moreover, nutrient + phytochemical and phytochemicals alone demonstrated favorable effects in elevating HDL-C and reducing TC. Vitamin E acts as an antioxidant by inhibiting lipid peroxidation and stabilizing lipoprotein structures (Mohd Zaffarin et al., 2020). Clinical studies have shown that higher vitamin E intake is positively correlated with plasma HDL-C levels (Wang et al., 2024). In addition, phytochemicals can activate multiple metabolic signaling pathways such as AMPK, thereby promoting fatty acid oxidation and remodeling cholesterol metabolism (Zhang et al., 2023). Rich in polyphenols and other antioxidant compounds with significant bioactivity, these plant-derived substances have shown significant efficacy in improving hepatic lipid metabolism and function (Luo et al., 2023).

Glucose metabolism disorder represents a central mechanism in the onset and progression of MAFLD. Its pathogenesis primarily involves impairment of the insulin signaling pathway, specifically alterations in insulin receptor (InsR), insulin receptor substrate 1 (IRS1), phosphatidylinositol 3-kinase (PI3K), and glucose transporter type 4 (GLUT4) (Emamgholipour et al., 2020), as well as mitochondrial dysfunction and sustained activation of oxidative stress. These changes collectively restrict the uptake and utilization of hepatic glucose, while promoting lipid accumulation and inflammatory responses, thereby perpetuating a vicious cycle of metabolic and inflammatory injury (Zhao et al., 2024). This study demonstrated that nutrient + pharmacological interventions (such as vitamin E combined with pioglitazone or pentoxifylline) achieved the most pronounced effects in HbA1c, insulin, and HOMA-IR. Pioglitazone, a peroxisome proliferator-activated receptor gamma (PPARγ) agonist, enhances insulin sensitivity and facilitates the redistribution of fatty acids, significantly improving hepatic histological features and insulin sensitivity (Lian and Fu, 2021). Vitamin E, by scavenging ROS and improving mitochondrial oxidative function, further promotes glucose oxidation (Pazdro and Burgess, 2010). Their synergistic effects establish a dual regulatory mechanism of metabolic restoration and antioxidant defense. These findings suggest that multi-component combination therapies, by synergistically modulating multiple targets, may provide more substantial and durable benefits for regulating glucose and lipid metabolism than monotherapy. Additionally, fenofibrate + pentoxifylline demonstrated the greatest efficacy in reducing ALT and AST, indicating synergistic hepatoprotective effects. Fenofibrate alleviates hepatic lipotoxicity by promoting fatty acid oxidation (Mahmoudi et al., 2022), whereas pentoxifylline mitigates inflammatory injury by inhibiting the release of TNF-α and improving microcirculation (Samlaska and Winfield, 1994). Furthermore, herbal extracts (e.g., silymarin) and hepatoprotective agents (e.g., UDCA) also exhibited beneficial effects in improving liver enzyme levels. With antioxidant and membrane-stabilizing effects, silymarin helps reduce hepatocellular injury induced by free radicals (Gillessen and Schmidt, 2020), while UDCA enhances bile excretion by modulating bile acid metabolism and apoptosis signaling pathways (Lazaridis et al., 2001).

Inflammatory responses play a pivotal role in the onset and progression of MAFLD. The underlying pathological mechanisms involve abnormal activation of immune cells, excessive release of pro-inflammatory cytokines, and the mutual amplification between oxidative stress and lipotoxicity (Chan et al., 2023). Numerous studies have highlighted the potential of natural antioxidants, particularly those derived from foods and plants, in the prevention and treatment of MAFLD. Many plant-based antioxidants, such as naringin, hesperidin, curcumin, and resveratrol, have been extensively investigated in both experimental models and clinical studies on MAFLD. These compounds have shown beneficial effects in reducing oxidative stress and inflammatory markers by modulating signaling pathways such as ERK, NF-κB, AMPKα, and PPARγ (Ezhilarasan and Langeswaran, 2024; Ezhilarasan and Lakshmi, 2022). The findings of this analysis demonstrated that phytochemicals (e.g., curcumin, resveratrol, and genistein) significantly reduced levels of IL-6 and TNF-α. Their mechanisms of action primarily involve the inhibition of the NF-κB a plant-nd MAPK signaling pathways, upregulation of Nrf2-mediated antioxidant genes, and suppression of pro-inflammatory cytokine transcription (Ajit et al., 2016; Shamsnia et al., 2023). Additionally, plant-based bioactive compounds, such as naringenin, hesperidin, and other bioactive compounds of propolis and saffron, have potential applications in overcoming oxidative stress and inflammation in MAFLD (Hua et al., 2021; Li et al., 2024; Kitamura, 2019; Xu et al., 2022). Rich in polyphenols and antioxidants, these compounds can synergize with pharmacological agents to enhance metabolic regulation and liver protection. Moreover, bioactive metabolic regulators, particularly L-carnitine, exert anti-inflammatory effects by enhancing fatty acid β-oxidation, reducing the accumulation of lipid and ROS, and thereby suppressing hepatic inflammation mediated by TNF-α (Alhasaniah, 2023). These results suggest that plant-based bioactive compounds and metabolic regulators can both relieve inflammation and regulate metabolism, underscoring their substantial therapeutic potential in the comprehensive management of MAFLD.

In terms of oxidative stress, herbal extracts were significantly effective in enhancing TAC. Meanwhile, L-carnitine demonstrated the strongest efficacy in decreasing MDA among all interventions. Previous meta-analyses have shown that L-carnitine supplementation lowers IL-6, TNF-α, and MDA levels while increasing SOD activity (Rastgoo et al., 2023; Fathizadeh et al., 2020), which aligns with the findings of this study. Notably, OEA, as an endogenous lipid regulator, exhibited consistent effects in both enhancing SOD activity and reducing MDA levels. This dual mechanism of action involves activating antioxidant defense systems and inhibiting lipid peroxidation. OEA is a potent agonist of peroxisome proliferator-activated receptor alpha (PPAR-α). It is capable of modulating the expression of fatty acid translocase CD36, thereby influencing feeding behavior (Sihag and Jones, 2018). By activating the PPAR-α pathway, OEA promotes fatty acid β-oxidation and improves the homeostasis of hepatic lipids (Chen et al., 2023). Animal studies have further shown that OEA exerts antioxidant effects by upregulating the expression of Nrf2 and HO-1, significantly decreasing hepatic MDA levels and increasing SOD activity (Hu et al., 2020). In addition, OEA participates in the regulation of fat utilization, energy balance, and intestinal motility. It serves as a key signaling molecule for maintaining energy homeostasis and feeding behavior (De Filippo et al., 2023). Given its dual mechanism of endogenous metabolic regulation and antioxidant defense, OEA may serve as a novel therapeutic option for the treatment of MAFLD.

Overall, the superior effects of combination interventions primarily stem from their mechanistic complementarity. Pharmacological agents mainly target insulin signaling, lipid metabolism, and inflammation, whereas dietary supplements and phytochemicals focus on redox balance, mitochondrial protection, and modulation of the gut microbiota. When these interventions are combined, they can regulate the whole chain from metabolism to the terminal phase of oxidative stress. Combination strategies such as nutrient + pharmacological intervention and pharmacological intervention + bioactive regulator exhibited consistent effects across multiple outcome indicators. These findings further demonstrated the potential therapeutic effect of multi-target, multi-mechanism, and synergistic interventions in the treatment of MAFLD. The SUCRA ranking results indicated that natural or endogenous regulators such as L-carnitine, garlic, and OEA demonstrated strong potential in enhancing antioxidant defense and mitigating oxidative damage. However, these findings are primarily based on indirect comparisons from RCTs and should therefore be interpreted cautiously, given the certainty of evidence and clinical applicability. Further high-quality studies are warranted to validate these findings.

### 4.1 Strengths, limitations, and future perspectives

This study has several notable strengths. First, to our knowledge, this is the first Bayesian NMA to systematically compare several dietary supplements, phytochemicals, bioactive metabolic regulators, and pharmacological agents for the management of MAFLD across multiple metabolism and oxidative stress-related outcomes. By integrating both direct and indirect evidence, the Bayesian NMA framework can help comprehensively compare and rank diverse interventions that are rarely evaluated head-to-head in randomized controlled trials. In addition, we evaluated multidimensional outcomes, including lipid profiles, glucose metabolism, inflammatory markers, liver enzymes, and oxidative stress indices, providing an integrated view of therapeutic effects, reflecting the complex and multifactorial pathology of MAFLD.

Several limitations of this study should be acknowledged. First, the number of studies available for certain outcome indicators was relatively small, which limits the credibility of indirect comparisons. Second, substantial differences were observed in intervention dosage and treatment duration among the included studies (ranging from 4 weeks to 18 months), potentially introducing heterogeneity. Nevertheless, most studies demonstrated consistent therapeutic effects within their treatment periods, and the distribution of treatment duration appeared to be random. Therefore, subgroup analyses were not further conducted. In addition, even for the same intervention (e.g., curcumin), the dosage varied markedly across studies (from 50 mg/day to 3 g/day), which may have influenced the accuracy of effect estimation and increased heterogeneity across studies. Since most dosages were determined based on prior literature or clinically tolerable ranges, and no systematic bias was observed, subgroup analysis by dose was not performed in this study. Future research should explore dose-response relationships under more standardized or quantifiable dosages to clarify the impact of different dosages on treatment efficacy. Furthermore, the included studies were mainly conducted in regions such as Iran, which might have affected the representativeness and generalizability of the results to some extent. However, studies from these regions were generally comparable in design quality, participant characteristics, and intervention types to those conducted elsewhere. Moreover, the Bayesian network model employed in this study integrates both direct and indirect evidence from multiple sources, which helps to mitigate potential bias caused by uneven geographic distributions.

In the future, multicenter, large-sample, and long-term follow-up RCTs are necessary to further validate these findings. Moreover, integrating multi-omics approaches, including lipidomics, metabolomics, and gut microbiome analyses, will provide deep insights into the comprehensive metabolic alterations in MAFLD. Lipidomics, in particular, can help identify specific lipid types associated with disease progression and treatment response, while metabolomics may uncover key metabolic pathways modulated by different interventions. These techniques, combined with transcriptomic analysis, could reveal the molecular mechanisms and biomarkers of therapeutic efficacy, offering valuable references for precision and personalized treatment of MAFLD.

## 5 Conclusion

Overall, this study provides systematic evidence-based support for comprehensive intervention strategies in MAFLD. A multi-target combination strategy that combines nutritional supplementation and pharmacological therapy may offer a novel direction for the prevention and treatment of MAFLD. Conducting multicenter, large-sample, long-term RCTs and metabolomic and mechanistic investigations is imperative to further validate the efficacy and safety of various interventions in regulating metabolism and antioxidant defense.

## Data Availability

The datasets presented in this study can be found in online repositories. The names of the repository/repositories and accession number(s) can be found in the article/Supplementary Material.
