# Supplementary material for: Efficacy of selected dietary supplements and pharmacological agents on metabolic and oxidative stress outcomes in metabolic dysfunction–associated fatty liver disease (MAFLD): a Bayesian network meta-analysis
Source: Front Pharmacol. 2026 Jan 29;16:1682688. doi: 10.3389/fphar.2025.1682688 (PMC12894030; doi:10.3389/fphar.2025.1682688)
Supplement: Supplementary file 1 [file Supplementaryfile1.docx]

**Text S1.** Supplementary Statistical Methods

############################################################

# Bayesian Network Meta-analysis Script (gemtc + rjags)

# R version: 4.4.1

# Packages: gemtc, rjags, coda, openxlsx, netmeta

# Description:

# Performs Bayesian random-effects Network Meta-Analysis

# following the protocol and methods described in the manuscript.

############################################################

## 0) Environment setup -----------------------------------------------------

set.seed(20250101)

# Install required packages (if not yet installed)

pkgs <- c("gemtc", "rjags", "coda", "openxlsx", "netmeta")

to_install <- pkgs[!pkgs %in% installed.packages()[, "Package"]]

if (length(to_install)) install.packages(to_install, dependencies = TRUE)

# Load libraries

library(gemtc)

library(rjags)

library(coda)

library(openxlsx)

library(netmeta)

# Output directory

out_dir <- "nma_outputs"

if (!dir.exists(out_dir)) dir.create(out_dir)

## 1) User-defined parameters -----------------------------------------------

# Input file and worksheet (example: TNF-α outcome)

data_file <- "Key_intervention_subgroup_analysis.xlsx"

sheet_name <- "TNF-α"

# Outcome name (used for naming results)

outcome_name <- "TNF-a"

# Direction of beneficial effect:

# TRUE = lower values are better (e.g., TG, ALT, MDA)

# FALSE = higher values are better (e.g., HDL-C, SOD, TAC)

lower_is_better <- TRUE

# Reference treatment for relative effects

ref_treatment <- "placebo"

# MCMC settings (consistent with manuscript)

n_chains <- 4

n_adapt <- 5000

n_iter <- 20000

thin <- 1

## 2) Data import and validation --------------------------------------------

dat <- read.xlsx(data_file, sheet = sheet_name)

required_cols <- c("study", "treatment", "mean", "sd", "sampleSize")

missing_cols <- setdiff(required_cols, names(dat))

if (length(missing_cols)) {

stop(paste("Missing required columns:", paste(missing_cols, collapse = ", ")))

}

cat("\nData preview:\n")

print(utils::head(dat))

cat("\nNumber of studies:", length(unique(dat$study)), "\n")

cat("Number of treatments:", length(unique(dat$treatment)), "\n")

## 3) Network construction and visualization --------------------------------

network <- mtc.network(data.ab = dat)

# Basic network plot (gemtc)

png(file.path(out_dir, paste0("network_", outcome_name, "_gemtc.png")),

width = 1200, height = 900, res = 150)

plot(network)

dev.off()

## 4) Bayesian random-effects model fitting ---------------------------------

model_random <- mtc.model(

network,

type = "consistency",

likelihood = "normal",

link = "identity",

linearModel = "random",

n.chain = n_chains

)

results_random <- mtc.run(

model_random,

n.adapt = n_adapt,

n.iter = n_iter,

thin = thin

)

# Convergence diagnostics (Gelman–Rubin)

png(file.path(out_dir, paste0("gelman_", outcome_name, ".png")),

width = 1200, height = 900, res = 150)

gelman.plot(results_random)

dev.off()

psrf <- gelman.diag(results_random, autoburnin = FALSE)

cat("\nPotential Scale Reduction Factors (PSRF):\n")

print(psrf)

# Save summary

sum_res <- summary(results_random)

capture.output(sum_res, file = file.path(out_dir, paste0("summary_", outcome_name, ".txt")))

## 5) Consistency assessment (Node-splitting method) ------------------------

ns <- mtc.nodesplit(

network,

likelihood = "normal",

link = "identity",

linearModel = "random",

n.chain = n_chains,

n.adapt = n_adapt,

n.iter = n_iter,

thin = thin

)

sum_ns <- summary(ns)

capture.output(sum_ns, file = file.path(out_dir, paste0("nodesplit_summary_", outcome_name, ".txt")))

png(file.path(out_dir, paste0("nodesplit_", outcome_name, ".png")),

width = 1200, height = 900, res = 150)

plot(sum_ns)

dev.off()

## 6) Heterogeneity evaluation (aNOHE / τ²) ---------------------------------

anohe_fit <- mtc.anohe(

network,

n.adapt = n_adapt,

n.iter = n_iter,

thin = thin,

n.chain = n_chains,

likelihood = "normal",

link = "identity",

linearModel = "fixed"

)

sum_anohe <- summary(anohe_fit)

capture.output(sum_anohe, file = file.path(out_dir, paste0("anohe_summary_", outcome_name, ".txt")))

png(file.path(out_dir, paste0("anohe_", outcome_name, ".png")),

width = 1200, height = 900, res = 150)

plot(sum_anohe)

dev.off()

## 7) Relative effects and forest plot --------------------------------------

if (!(ref_treatment %in% network$treatments$treatment)) {

stop(paste0("Reference treatment '", ref_treatment,

"' not found in the dataset. Please adjust the name."))

}

rel_eff <- relative.effect(results_random, ref_treatment)

png(file.path(out_dir, paste0("forest_vs_", ref_treatment, "_", outcome_name, ".png")),

width = 1200, height = 1800, res = 150)

gemtc::forest(rel_eff,

main = paste0("Relative effects vs ", ref_treatment,

" (", outcome_name, ")"))

dev.off()

## 8) Ranking probabilities and SUCRA --------------------------------------

rank_prob <- rank.probability(results_random, lower.better = lower_is_better)

sucra_out <- sucra(rank_prob)

# Export rankings

write.csv(rank_prob,

file.path(out_dir, paste0("rank_prob_", outcome_name, ".csv")),

row.names = TRUE)

write.csv(sucra_out,

file.path(out_dir, paste0("sucra_", outcome_name, ".csv")),

row.names = TRUE)

# Plot ranking probabilities

png(file.path(out_dir, paste0("rank_plot_", outcome_name, ".png")),

width = 1200, height = 900, res = 150)

plot(rank_prob, main = paste0("Rank probabilities (", outcome_name, ")"))

dev.off()

## 9) League table (pairwise relative effects) ------------------------------

ltab <- round(relative.effect.table(results_random), 3)

write.csv(ltab, file.path(out_dir, paste0("league_table_", outcome_name, ".csv")))

## 10) Fixed-effect sensitivity analysis (optional) -------------------------

model_fixed <- mtc.model(

network,

type = "consistency",

likelihood = "normal",

link = "identity",

linearModel = "fixed",

n.chain = n_chains

)

results_fixed <- mtc.run(

model_fixed,

n.adapt = n_adapt,

n.iter = n_iter,

thin = thin

)

sum_fixed <- summary(results_fixed)

capture.output(sum_fixed,

file = file.path(out_dir, paste0("summary_fixed_", outcome_name, ".txt")))

cat("\nAnalysis completed successfully. Results saved in:\n",

normalizePath(out_dir), "\n")
