# Supplementary material for: Efficacy of selected dietary supplements and pharmacological agents on metabolic and oxidative stress outcomes in metabolic dysfunction–associated fatty liver disease (MAFLD): a Bayesian network meta-analysis
Source: Front Pharmacol. 2026 Jan 29;16:1682688. doi: 10.3389/fphar.2025.1682688 (PMC12894030; doi:10.3389/fphar.2025.1682688)
Supplement: Supplementary file 2 [file Supplementaryfile3.docx]

**Table S1**. Baseline characteristics of the included RCTs

|  |  |  |  |  |  | Intervention mode | | | | | Number of cases | | | | |
| --- | --- | --- | --- | --- | --- | --- | --- | --- | --- | --- | --- | --- | --- | --- | --- |
| First author | Year | Study type | Country | Treatment time | Type  of  Group | Group1 | Group2 | Group3 | Group4 | Group5 | Group1 | Group2 | Group3 | Group4 | Group5 |
| Celinski et al[18] | 2014 | RCT | Poland | 14 months | NAFLD | Essentiale(3 tablet/day)and Tryptophan (1000mg/day) | Essentiale(3 tablet/day) and melatonin(10 mg/day) | Essentiale(3 tablet/day) |  |  | 28 | 23 | 23 |  |  |
| Chachay et al[19] | 2014 | RCT | Australia | 8 weeks | NAFLD | Resveratrol(3000 mg/day) | Placebo |  |  |  | 10 | 10 |  |  |  |
| Choi et al[20] | 2014 | RCT | Korea | 6 weeks | NAFLD | Human placental extract | A commercially available mixture of liver extract and flavin adenine dinucleotide |  |  |  | 70 | 84 |  |  |  |
| Faghihzadeh et al[21] | 2014 | RCT | Iran | 12 weeks | NAFLD | Resveratrol(500 mg/day) | Placebo |  |  |  | 25 | 25 |  |  |  |
| Farhangi et al[22] | 2014 | RCT | Iran | 4 weeks | NAFLD | Coenzyme Q10(100 mg/day) | Placebo |  |  |  | 20 | 21 |  |  |  |
| Han et al[23] | 2014 | RCT | China | 24-week | NAFLD | Bicyclol(25 mg three times daily) | Vtamin E(100 mg three times daily) |  |  |  | 112 | 111 |  |  |  |
| Mohammadshahi et al[24] | 2014 | RCT | Iran | 12 weeks | NAFLD | Coenzyme Q10(100 mg/day) | Placebo |  |  |  | 20 | 21 |  |  |  |
| Sharifi et al[25] | 2014 | RCT | Iran | 4 months | NAFLD | Vitamin D3(50,000 IU) | Placebo |  |  |  | 27 | 26 |  |  |  |
| Solhi et al[26] | 2014 | RCT | Iran | 8 weeks | NAFLD | Silymarin(70 mg tablet of silymarin 3 times a day) | Placebo |  |  |  | 33 | 31 |  |  |  |
| Aller et al[27] | 2015 | RCT | Spain | 3 months | NAFLD | Silymarin(2 tablets per day) plus  vitamin E (36 mg) | Lifestyle modification program |  |  |  | 18 | 18 |  |  |  |
| Amiri-Moghadam et al[28] | 2015 | RCT | Iran | 12 weeks | NASH | L-carnitine(2000 mg/d) | Placebo |  |  |  | 36 | 32 |  |  |  |
| Chen et al[29] | 2015 | RCT | China | 3 months | NAFLD | Resveratrol(600 mg/day) | Placebo |  |  |  | 30 | 30 |  |  |  |
| El-Haggar et al[30] | 2015 | RCT | Egypt | 24 weeks | NAFLD | Fenofibrate(300 mg daily) | Fenofibrate(300 mg daily) plus pentoxifylline(1200 mg/day) |  |  |  | 45 | 45 |  |  |  |
| Faghihzadeh et al[31] | 2015 | RCT | Iran | 12 weeks | NAFLD | Resveratrol(500 mg/d) | Placebo |  |  |  | 24 | 24 |  |  |  |
| Guy et al[32] | 2015 | RCT | United States of America | 96 weeks | NASH | Vitamin E(800  IU daily) | Placebo |  |  |  | 30 | 29 |  |  |  |
| Sorrentino et al[33] | 2015 | RCT | Italy | 90 days | NAFLD | Silibinin (125 mg/d) of and vitamin E (30 IU/d) | Standard regimen of diet and exercise |  |  |  | 43 | 35 |  |  |  |
| Ebrahimi-Mameghani et al[34] | 2016 | RCT | Iran | 8 weeks | NAFLD | Chlorella vulgaris(300 mg/d) | Placebo |  |  |  | 29 | 26 |  |  |  |
| Ekhlasi et al[35] | 2016 | RCT | Iran | 8 weeks | NAFLD | Vitamin E(400 IU/d) | Placebo |  |  |  | 15 | 15 |  |  |  |
| Federico et al[36] | 2016 | RCT | Italy | 12 months | NAFLD | Silybin | Placebo |  |  |  | 10 | 17 |  |  |  |
| Heebøll et al[37] | 2016 | RCT | Denmark | 6 months | NAFLD | Resveratrol(1.5 g daily) | Placebo |  |  |  | 13 | 13 |  |  |  |
| Hong et al[38] | 2016 | RCT | South Korea | 3 weeks | NAFLD | Red Ginseng(3000  mg/d) | Placebo |  |  |  | 35 | 31 |  |  |  |
| Panahi et al[39] | 2016 | RCT | Iran | 8 weeks | NAFLD | Curcumin(1000 mg/day) | Lifestyle modification program |  |  |  | 44 | 43 |  |  |  |
| Rahimlou et al[40] | 2016 | RCT | Iran | 12 weeks | NAFLD | Ginger(2g/d) | Placebo |  |  |  | 22 | 21 |  |  |  |
| Rahmani et al[41] | 2016 | RCT | Iran | 8 weeks | NAFLD | Curcumin(500 mg/day) | Placebo |  |  |  | 37 | 40 |  |  |  |
| Rangboo et al[42] | 2016 | RCT | Iran | two months | NAFLD | Cynara scolymus(6 tablets per day consisting  of 2700mg extract of the herb) | Placebo |  |  |  | 30 | 30 |  |  |  |
| Ebrahimi-Mameghani et al[43] | 2017 | RCT | Iran | 8 weeks | NAFLD | Chlorella vulgaris(300md/d) | Placebo |  |  |  | 29 | 26 |  |  |  |
| Ghaffari et al[44] | 2017 | RCT | Iran | 12 weeks | NAFLD | Curcumin(3g/d) | Placebo |  |  |  | 21 | 21 |  |  |  |
| Navekar et al[45] | 2017 | RCT | Iran | 12 weeks | NAFLD | Turmeric capsules(3000mg/d) | Placebo |  |  |  | 21 | 21 |  |  |  |
| Pakravan et al[46] | 2017 | RCT | Iran | 3 months | NAFLD | Melatonin(orally twice daily) | Placebo |  |  |  | 49 | 48 |  |  |  |
| Pakzad et al[47] | 2017 | RCT | Iran | 8 weeks | NAFLD | Curcumin(50mg/d) | Placebo |  |  |  | 50 | 50 |  |  |  |
| Panahi et al[48] | 2017 | RCT | Iran | 8 weeks | NAFLD | Curcumin(1000 mg/day) | Placebo |  |  |  | 44 | 43 |  |  |  |
| Polyzos et al[49] | 2017 | RCT | Greece | 52-week | NAFLD | Spironolactone(25 mg once daily)  and vitamin E(400 IU/day in two  equal doses) | Vitamin E(400 IU/day in two  equal doses) |  |  |  | 17 | 14 |  |  |  |
| Zöhrer et al[50] | 2017 | RCT | Italy | 6 months | NASH | A mix containing docosahexaenoic acid(250mg/d), choline(201mg/d) and vitamin E(30UI/D) | Placebo |  |  |  | 20 | 20 |  |  |  |
| Amanat et al[51] | 2018 | RCT | Iran | 8 weeks | NAFLD | Genistein(250mg/d) | Placebo |  |  |  | 41 | 37 |  |  |  |
| Amirkhizi et al[52] | 2018 | RCT | Iran | 12 weeks | NAFLD | Alpha-lipoic acid(1200 mg/d) | Placebo |  |  |  | 25 | 25 |  |  |  |
| Asghari et al[53] | 2018 | RCT | Iran | 12 weeks | NAFLD | Resveratrol(600 mg/d) | Calorie-restricted diet | Placebo |  |  | 30 | 30 | 30 |  |  |
| Ghaffari et al[54] | 2018 | RCT | Iran | 12 weeks | NAFLD | Turmeric(3 g/d)and chicory(9 g/d) | Placebo |  |  |  | 21 | 21 |  |  |  |
| Kantartzis et al[55] | 2018 | RCT | Germany | 12 weeks | NAFLD | Resveratrol(150mg/d) | Placebo |  |  |  | 54 | 54 |  |  |  |
| Hosseinpour-Arjmand et al[56] | 2018 | RCT | Iran | 12 weeks | NAFLD | Alpha-lipoic acid(1200 mg/d) | Placebo |  |  |  | 23 | 22 |  |  |  |
| Pervez et al[57] | 2018 | RCT | Pakistan | 12 weeks | NAFLD | δ-tocotrienol(300 mg twice daily) | Placebo |  |  |  | 31 | 33 |  |  |  |
| Saadati et al[58] | 2018 | RCT | Iran | 3 months | NAFLD | Curcumin(1.5g/d) | Placebo |  |  |  | 25 | 21 |  |  |  |
| Anushiravani et al[59] | 2019 | RCT | Iran | 3 months | NAFLD | Metformin (500 mg/day) | Silymarin(140 mg/day) | Pioglithasone(15 mg/day) | Vitamin E(400 IU/day) | Placebo | 30 | 30 | 30 | 30 | 30 |
| Bril et al[60] | 2019 | RCT | Florida | 18 months | NAFLD+T2DM | Vitamin E(400 IU b.i.d) | Vitamin E(400 IU b.i.d) +pioglitazone(45 mg/day) | Placebo |  |  | 36 | 37 | 32 |  |  |
| Chashmniam et al[61] | 2019 | RCT | Iran | 8 weeks | NAFLD | Phospholipid  curcumin(250 mg/d) | Placebo |  |  |  | 25 | 20 |  |  |  |
| Cheraghpour et al[62] | 2019 | RCT | Iran | 12 weeks | NAFLD | Hesperidin(1g/d) | Placebo |  |  |  | 25 | 24 |  |  |  |
| Jazayeri-Tehrani et al[63] | 2019 | RCT | Iran | 3 months | NAFLD+ overweight and obese patients | Curcumin(40mg/day) | Placebo |  |  |  | 42 | 42 |  |  |  |
| Mirhafez et al[64] | 2019 | RCT | Iran | 8 weeks | NAFLD | Curcumin(250 mg/day) | Placebo |  |  |  | 32 | 29 |  |  |  |
| Nobili et al[65] | 2019 | RCT | Italy | 4 months | NAFLD | VitaminE(10mg/d) and Hydroxytyrosol(7.5mg/d) | Placebo |  |  |  | 35 | 35 |  |  |  |
| Oliveira et al[66] | 2019 | RCT | Brasil | 48 weeks | NAFLD | Nacetylcysteine(1.2g)+ursodeoxycholic acid(15mg/kg)+metformin(850-1500mg/day) | Ursodeoxycholic acid (20 mg/kg) + metformin (850-1500 mg/day) | Nacetylcysteine (1.2g) + metformin (850-1500 mg/day) |  |  | 20 | 6 | 12 |  |  |
| Panahi et al[67] | 2019 | RCT | Iran | 12 weeks | NAFLD | Curcuminoids coadministere(500 mg/d) with piperine (5 mg/d) | Placebo |  |  |  | 35 | 35 |  |  |  |
| Rahmanabadi et al[68] | 2019 | RCT | Iran | 12 weeks | NAFLD | α-lipoic acid(1200 mg/day) | Placebo |  |  |  | 25 | 25 |  |  |  |
| Rashidmayvan et al[69] | 2019 | RCT | Iran | 8 weeks | NAFLD | Nigella sativa(1g/d) | Placebo |  |  |  | 22 | 22 |  |  |  |
| Saadati et al[70] | 2019 | RCT | Iran | 12 weeks | NAFLD | Curcumin(1500 mg) | Placebo |  |  |  | 27 | 23 |  |  |  |
| Saadati et al[71] | 2019 | RCT | Iran | 12 weeks | NAFLD | Curcumin(1500 mg) | Placebo |  |  |  | 25 | 23 |  |  |  |
| Torquato et al[72] | 2019 | RCT | Italy | 12 months | NASH | Docosahexaenoic acid-based multivitamin | Placebo |  |  |  | 27 | 15 |  |  |  |
| Bahrami et al[73] | 2020 | RCT | Iran | 12 weeks | NAFLD | Melatonin(6 mg/d) | Placebo |  |  |  | 24 | 21 |  |  |  |
| Cerletti et al[74] | 2020 | RCT | Italy | 3 months | NAFLD | A mixture of active ingredients(DHA 250 mg+ phosphatidylcholine 150 mg+ silymarin 75 mg+ choline bitartrate  35 mg+ , curcumin 35 mg+ d D-α-tocopherol 10 mg) | Lifestyle modification program |  |  |  | 55 | 58 |  |  |  |
| Farzin et al[75] | 2020 | RCT | Iran | 12 weeks | NAFLD | Resveratrol(600 mg/d) | Placebo |  |  |  | 25 | 25 |  |  |  |
| Fathi et al[76] | 2020 | RCT | Iran | 12 weeks | NAFLD+ Overweight/Obese Patients | Zinc(30 mg/d) | Placebo |  |  |  | 25 | 25 |  |  |  |
| Fathi et al[77] | 2020 | RCT | Iran | 12 weeks | NAFLD+ Overweight/Obese Patients | Zinc(30 mg/d) | Placebo |  |  |  | 25 | 25 |  |  |  |
| Ferro et al[78] | 2020 | RCT | Italy | 12 weeks | NAFLD | Bergamot  Citrus and Wild Cardoon(300 mg/d) | Placebo |  |  |  | 45 | 41 |  |  |  |
| Hariri et al[79] | 2020 | RCT | Iran | 8 weeks | NAFLD | Curcumin (250 mg/day) | Placebo |  |  |  | 23 | 22 |  |  |  |
| Moradi Kelardeh et al[80] | 2020 | RCT | Iran | 12 weeks | NAFLD | Resistance training (RT) | Curcumin | RT with curcumin | Placebo |  | 12 | 11 | 11 | 11 |  |
| Pervez et al[81] | 2020 | RCT | Pakistan | 24 weeks | NAFLD | δ-tocotrienol(300 mg twice daily) | Placebo |  |  |  | 35 | 36 |  |  |  |
| Pour et al[82] | 2020 | RCT | Iran | 12 weeks | NAFLD | Saffron(100 mg/d) | Placebo |  |  |  | 36 | 36 |  |  |  |
| Rafie et al[83] | 2020 | RCT | Iran | 12 weeks | NAFLD | Ginger(500 mg/d) | Placebo |  |  |  | 23 | 23 |  |  |  |
| Saberi-Karimian et al[84] | 2020 | RCT | Iran | 8 weeks | NAFLD | Curcuminoids(500 mg) | Placebo |  |  |  | 23 | 26 |  |  |  |
| Sangouni et al[85] | 2020 | RCT | Iran | 12 weeks | NAFLD | Garlic(400 mg/d) | Placebo |  |  |  | 45 | 43 |  |  |  |
| Amerikanou et al[86] | 2021 | RCT | Greece, Italy, Serbia | 6 months | NAFLD | Mastiha(2.1 g/d) | Placebo |  |  |  | 41 | 57 |  |  |  |
| Darvish Damavandi et al[87] | 2021 | RCT | Iran | 12 weeks | NAFLD | Purslane extract(300 mg/d) | Placebo |  |  |  | 37 | 34 |  |  |  |
| Domech et al[88] | 2021 | RCT | Cuba | 24 weeks | NAFLD | Abexol(50 mg/d) | Atorvastatin(10 mg) |  | Placebo |  | 22 | 23 | 24 | 23 |  |
| Fouda et al[89] | 2021 | RCT | Egypt | 3 months | NAFLD | Vitamin E(400  mg twice a day) | Ursodeoxycholic Acid(250 mg  twice a day) | Pentoxifylline(400 mg  twice daily) |  |  | 30 | 31 | 33 |  |  |
| Harrison et al[90] | 2021 | RCT | USA | 16 weeks | NAFLD | AXA1125(3g bid) | Placebo |  |  |  | 32 | 15 |  |  |  |
| He et al[91] | 2021 | RCT | China | 12 weeks | NAFLD | Vitamin C(250 mg/day) | Vitamin C(1000 mg/day) | Vitamin C(2000 mg/day) |  |  | 26 | 30 | 28 |  |  |
| Jarhahzadeh et al[92] | 2021 | RCT | Iran | 8 weeks | NAFLD | Turmeric(2 g/day) | Placebo |  |  |  | 32 | 32 |  |  |  |
| Kedarisetty et al[93] | 2021 | RCT | India | 12 months | NAFLD | Pentoxiphylline (400 mg thrice daily) and vitamin E(400 IU twice daily) | Vitamin E(400 IU twice daily) |  |  |  | 36 | 33 |  |  |  |
| Kooshki et al[94] | 2021 | RCT | Iran | 12 weeks | NAFLD | Chromium picolinate(400µg /d) | Placebo |  |  |  | 22 | 21 |  |  |  |
| Majnooni et al[95] | 2021 | RCT | Iran | 12 weeks | NAFLD | Metformin(1000 mg/day)+ vitamin E (400 UI/day) | Metformin(1000 mg/day)+ artichoke leaf extract(800 mg/day) | artichoke leaf extract(800 mg/day)+ vitamin E (400 UI/day) |  |  | 30 | 30 | 30 |  |  |
| Mirhafez et al[96] | 2021 | RCT | Iran | 8 weeks | NAFLD | Curcumin(250 mg/day) | Placebo |  |  |  | 35 | 37 |  |  |  |
| Namkhah et al[97] | 2021 | RCT | Iran | 4 weeks | NAFLD | Naringenin(100 mg/d) | Placebo |  |  |  | 22 | 22 |  |  |  |
| Yari et al[98] | 2021 | RCT | Iran | 12 weeks | NAFLD | Hesperidin | Lifestyle modification program |  |  |  | 22 | 21 |  |  |  |
| Akbari et al[99] | 2022 | RCT | Iran | 8 weeks | NAFLD | Rosemary leaf(4 g/day) | Placebo |  |  |  | 57 | 53 |  |  |  |
| Arefhosseini et al[100] | 2022 | RCT | Iran | 8 weeks | NAFLD | Hydroxy citric acid(3000mg/day) | Calorie-restricted diet |  |  |  | 21 | 19 |  |  |  |
| Atarodi et al[101] | 2022 | RCT | Iran | 4 weeks | NAFLD | Silymarin(140 mg every 8 h) | Placebo |  |  |  | 27 | 29 |  |  |  |
| Ferro et al[102] | 2022 | RCT | Italy | 6 weeks | NAFLD | Citrus Bergamia and Cynara Cardunculus(300 mg/day) | Placebo |  |  |  | 47 | 47 |  |  |  |
| Hosseinian et al[103] | 2022 | RCT | Iran | 2 months | NAFLD | Curcumin(250 mg/day) | Placebo |  |  |  | 35 | 37 |  |  |  |
| Kalhori et al[104] | 2022 | RCT | Iran | 12 weeks | NAFLD | Turmeric(3000mg/d) | Placebo |  |  |  | 21 | 21 |  |  |  |
| Mohammadi et al[105] | 2022 | RCT | Iran | 6 months | NAFLD | Metformin (500mg daily) | Melatonin (10mg daily) | Vitamin E(800 IU daily) | Placebo |  | 37 | 35 | 35 | 35 |  |
| Naeini et al[106] | 2022 | RCT | Tehran | 4 weeks | NAFLD | Naringenin(100mg/d) | Placebo |  |  |  | 22 | 22 |  |  |  |
| Pervez et al[107] | 2022 | RCT | Pakistan | 48 weeks | NAFLD | δ-tocotrienol(300 mg, twice daily) | r α-tocopherol(268 mg, twice daily) |  |  |  | 50 | 50 |  |  |  |
| Rashidmayvan et al[108] | 2022 | RCT | Iran | 8 weeks | NAFLD | Nigella sativa(1000 mg/day) | Placebo |  |  |  | 22 | 22 |  |  |  |
| Rostamizadeh et al[109] | 2022 | RCT | Iran | 12 weeks | NAFLD | Licorice root extract(1000 mg/day) | Placebo |  |  |  | 28 | 24 |  |  |  |
| Wang et al[110] | 2022 | RCT | China | 16 weeks | NAFLD | Rosiglitazone(4 mg/time, once a day) | SiliBinin(3 × 35 mg) | Danshao Shugan Granule(2 packages, 3 times a day) |  |  | 30 | 50 | 130 | 50 |  |
| Xie et al[111] | 2022 | RCT | China | 8 weeks | NAFLD | Jianpi Xiaozhi Recipe(150 ml/dose, divided into 2 doses)+ Polyene phosphatidylcholine gum  Capsule (456 mg, 3 times/d)+Bicyclic alcohol (25 mg, 3 times) | Polyene phosphatidylcholine gum  Capsule (456 mg, 3 times/d)+Bicyclic alcohol (25 mg, 3 times) |  |  |  | 63 | 63 |  |  |  |
| Zojaji et al[112] | 2022 | RCT | Iran | 2 months | NAFLD | Apium graveolens, Citrus aurantium, Cynara scolymus, Portulaca oleracea, Silybum marianum compound (2 capsules, thrice daily) | Healthy diet |  |  |  | 30 | 16 |  |  |  |
| Beheshti Namdar et al[113] | 2023 | RCT | Iran | 2 months | NAFLD | Curcumin(160 mg/d) | Placebo |  |  |  | 29 | 27 |  |  |  |
| Damavandi et al[114] | 2023 | RCT | Iran | 12 weeks | NAFLD | Purslane extract(300 mg/day) | Placebo |  |  |  | 37 | 37 |  |  |  |
| Ghoreishi et al[115] | 2023 | RCT | Iran | 3 months | NAFLD+T2DM | Ginger powder(1000 mg, twice daily) | Placebo |  |  |  | 36 | 36 |  |  |  |
| Khoshbaten et al[116] | 2023 | RCT | Iran | 6 months | NAFLD | Pioglitazone(15 mg/d)+ Vit E(400IU/d) | Metformin(1000mg)+ Vit E(400IU/d) |  |  |  | 34 | 34 |  |  |  |
| Majeed et al[117] | 2023 | RCT | India | 90 days | NAFLD | Garcinol(50 mg/d)+ curcuminoids(250 mg/d)+ piperine(5 mg/d) | Placebo |  |  |  | 32 | 31 |  |  |  |
| Mojtahedi et al[118] | 2023 | RCT | Iran | 3 months | NAFLD | Melatonin(10 mg once a day) | Metformin(500 mg, twice a day) | Ursodeoxycholic acid(300 mg, twice a  day) | Placebo |  | 30 | 30 | 30 | 30 |  |
| Rezaei et al[119] | 2023 | RCT | Iran | 8 weeks | NAFLD | Zinc(30 mg/day) | Placebo |  |  |  | 25 | 25 |  |  |  |
| Safari et al[120] | 2023 | RCT | Iran | 12 weeks | NAFLD | Curcumin(250 mg/d) | Placebo |  |  |  | 28 | 28 |  |  |  |
| Sharifi et al[121] | 2023 | RCT | Iran | 12 weeks | NAFLD | Curcumin(500 mg/day)+piperine(5mg/day) | Placebo |  |  |  | 30 | 30 |  |  |  |
| Tutunchi et al[122] | 2023 | RCT | Iran | 12 weeks | NAFLD | Oleoylethanolamide(250 mg/day) | Placebo |  |  |  | 30 | 30 |  |  |  |
| Tutunchi et al[123] | 2023 | RCT | Iran | 8 weeks | NAFLD | A-lipoic acid(1200 mg/d) | Myo-inositol(4 g/d) | Propolis(500 mg/d) | Dietary  recommendation |  | 21 | 23 | 24 | 24 |  |

**Table S2.** Classification of Interventions Included in the Network Meta-analysis

| Main Category | Code | Subcategory / Intervention | Description / Mechanistic Notes |
| --- | --- | --- | --- |
| Ⅰ. Vitamins and Minerals (A) | A | Vitamin E (α-tocopherol, δ-tocotrienol) | Lipid-soluble antioxidant vitamins of the vitamin E family. |
|  | A | Vitamin C | Water-soluble antioxidant vitamin. |
|  | A | Vitamin D | Fat-soluble vitamin regulating calcium homeostasis and insulin sensitivity. |
|  | A | Zinc supplementation | Trace mineral, cofactor of antioxidant enzymes (e.g., SOD). |
|  | A | Chromium picolinate | Trace mineral with insulin-sensitizing effects. |
| Ⅱ. Bioactive Metabolic Regulators (B) | B | α-Lipoic acid | Endogenous antioxidant and metabolic cofactor. |
|  | B | Melatonin | Circadian and oxidative stress regulator. |
|  | B | Myo-inositol | Endogenous insulin-sensitizing molecule. |
|  | B | L-Carnitine | Facilitates mitochondrial β-oxidation of fatty acids. |
|  | B | Abexol | Beeswax alcohols with antioxidant and anti-inflammatory properties. |
|  | B | Coenzyme Q10 | Mitochondrial electron transporter and antioxidant. |
|  | B | L-Tryptophan | Amino acid precursor of serotonin, modulating oxidative metabolism. |
|  | B | Human placental extract | Peptide-rich biological extract with hepatoprotective and antioxidant effects. |
|  | B | Liver extract + FAD | Source of metabolic cofactors supporting oxidative enzymes. |
|  | B | AXA1125 / AXA1957 | Engineered amino acid formulations for mitochondrial dysfunction. |
|  | B | Vitamin E + Hydroxytyrosol (HXT) | Combination of lipophilic and phenolic antioxidants. |
|  | B | DHA-based multivitamin / mixture (DHA, phosphatidylcholine, silymarin, choline, curcumin, D-α-tocopherol) | Multi-component bioactive formulation for metabolic regulation. |
| Ⅲ. Low-Calorie Diet (C) | C | Dietary recommendation only | Nutritional intervention focusing on calorie restriction or healthy eating. |
| Ⅳ. Phytochemicals  (D) | D | Curcumin | It is a single purified active compound extracted from turmeric, representing a typical phytochemical (polyphenol). |
|  | D | Resveratrol | Stilbene polyphenol regulating oxidative stress and lipid metabolism. |
|  | D | Genistein | Isoflavone from soy with estrogenic and antioxidant activities. |
|  | D | Naringenin | Citrus flavonoid improving lipid metabolism and oxidative balance. |
|  | D | Hesperidin | Citrus flavonoid with vascular and antioxidant properties. |
|  | D | Garcinol + Curcuminoids + Piperine | Polyphenolic complex with synergistic antioxidant effects. |
|  | D | Propolis | Bee product rich in flavonoids and phenolic acids. |
|  | D | Hydroxycitric acid (Garcinia cambogia extract) | Organic acid derivative modulating lipogenesis. |
|  | D | Saffron (Crocus sativus L.) | Carotenoid-derived plant bioactive containing crocin and safranal. |
| Ⅴ. Herbal Extracts  (E) | E | Garlic (Allium sativum) | Herbal extract with lipid-lowering and antioxidant effects. |
|  | E | Ginger (Zingiber officinale) | Herbal extract with anti-inflammatory and digestive benefits. |
|  | E | Turmeric (Curcuma longa) | Herbal extract with antioxidant, anti-inflammatory, and hepatoprotective properties. |
|  | E | Purslane (Portulaca oleracea) | Plant extract with omega-3 fatty acids and antioxidants. |
|  | E | Artichoke (Cynara scolymus) | Herbal extract with hepatoprotective and lipid-lowering properties. |
|  | E | Licorice (Glycyrrhiza glabra) | Herbal extract with anti-inflammatory and hepatoprotective actions. |
|  | E | Nigella sativa (Black cumin) | Herbal extract with antioxidant and metabolic benefits. |
|  | E | Korean Red Ginseng (Panax ginseng) | Herbal extract improving glucose and lipid metabolism. |
|  | E | Rosemary (Rosmarinus officinalis) | Herbal extract with phenolic antioxidants (rosmarinic acid). |
|  | E | Apium graveolens | Celery seed extract with hepatoprotective and lipid-regulating activity. |
|  | E | Citrus aurantium | Bitter orange extract with metabolic effects. |
|  | E | Cynara cardunculus | Cardoon extract, related to artichoke, with hepatoprotective effects. |
|  | E | Citrus bergamia, Cynara cardunculus | Combined plant extract with synergistic metabolic actions. |
|  | E | Chlorella vulgaris | Herbal (microalgal) extract with antioxidant, hepatoprotective, and metabolic regulatory effects. |
|  | E | Mastiha (Pistacia lentiscus resin) | Resinous extract with antioxidant and anti-inflammatory activity. |
|  | E | Cichorium intybus (Chicory seed) | Herbal extract improving lipid and glucose metabolism. |
|  | E | Silymarin / Silibinin (Silybum marianum) | Standardized milk thistle extract rich in flavonolignans. |
|  | E | Mixed herbal capsule (A. graveolens, C. aurantium, C. scolymus, P. oleracea, S. marianum) | Multi-plant standardized herbal mixture. |
| Ⅵ. Polyherbal Formulas  (F) | F | Jianpi Xiaozhi Recipe | Traditional Chinese multi-herbal formula improving digestion and lipid metabolism. |
|  | F | Danshao Shugan Granule (DSSG) | Multi-herbal TCM formula with hepatoprotective and anti-inflammatory actions. |
| Ⅶ. Pharmacological Agents  (G-L) | G | Metformin | Insulin sensitizer improving hepatic and peripheral insulin sensitivity. |
|  | H | Thiazolidinediones (TZDs): Pioglitazone, Rosiglitazone | PPAR-γ agonists enhancing insulin sensitivity and reducing hepatic steatosis. |
|  | I | Lipid-lowering agents: Fenofibrate, Atorvastatin | Modulate lipid metabolism via PPAR-α and HMG-CoA reductase inhibition. |
|  | J | Hepatoprotective agents: Ursocholic acid （UDCA）, Polyene phosphatidylcholine + Bicyclol, Bicyclol, Essentiale forte | Improve bile flow, membrane stability, and reduce hepatocellular injury. |
|  | K | Vasodilator: Pentoxifylline | Reduces TNF-α and improves hepatic microcirculation. |
|  | L | Endogenous lipid regulator: Oleoylethanolamide (OEA) | Endogenous PPAR-α agonist regulating lipid and energy metabolism. |
| Ⅷ. Combined Interventions  (M-R) | M | Nutrient–Pharmacological: Pioglitazone + Vitamin E; Metformin + Vitamin E; Pentoxifylline + Vitamin E; Spironolactone + Vitamin E | Combination of pharmacological therapy with antioxidant vitamins. |
|  | N | Phytochemical–Pharmacological: Metformin + Artichoke Leaf Extract | Drug combined with plant-derived extract to enhance metabolic and hepatoprotective effects. |
|  | O | Nutrient–Phytochemical: Vitamin E + Artichoke Leaf Extract; Vitamin E + Silymarin + Carnitine; Silibinin + Vitamin E | Vitamin and plant bioactive combinations providing synergistic antioxidative benefits. |
|  | P | Pharmacological–Bioactive Regulator: Metformin + N-acetylcysteine; Abexol + Atorvastatin; Essentiale forte+tryptophan | Integration of drug therapy with metabolic bioactive modulators. |
|  | Q | Pharmacological–Pharmacological: Metformin + UDCA; Fenofibrate + Pentoxifylline | Dual-drug interventions targeting complementary metabolic and inflammatory pathways. |
|  | R | Phytochemical–Herbal: Danshao Shugan Granule + Silibinin | Combination of a multi-herbal TCM formula and a standardized plant extract. |

Notes: Interventions were categorized according to their principal biochemical characteristics, mechanism of action, and clinical usage. Vitamins and Minerals include essential micronutrients; Bioactive Metabolic Regulators are endogenous or nutrient-derived modulators of oxidative stress; Phytochemicals are standardized plant-derived actives; Herbal Extracts refer to complex mixtures; Polyherbal Formulas denote traditional multi-herbal combinations; Pharmacological Agents were grouped by mechanism; Combined Interventions were defined by mechanistic complementarity.

**Table S3.** Risk assessment form

| Study | Entry | Risk levek |
| --- | --- | --- |
| Celinski et al,2014 | Random sequence generation(selection bias) | Unclear |
|  | Allocation concealment(selection bias) | Unclear |
|  | Blinding of participants and personnel (performance bias) | Unclear |
|  | Blinding of outcome assessment(detection bias) | Unclear |
|  | Incomplete outcome data(attrition bias) | Low |
|  | Selective reporting(reporting bias) | Low |
|  | Other bias | Low |
| Chachay et al,2014 | Random sequence generation(selection bias) | Low |
|  | Allocation concealment(selection bias) | Low |
|  | Blinding of participants and personnel (performance bias) | Low |
|  | Blinding of outcome assessment(detection bias) | Low |
|  | Incomplete outcome data(attrition bias) | Low |
|  | Selective reporting(reporting bias) | Low |
|  | Other bias | Low |
| Choi et al,2014 | Random sequence generation(selection bias) | Unclear |
|  | Allocation concealment(selection bias) | Unclear |
|  | Blinding of participants and personnel (performance bias) | High |
|  | Blinding of outcome assessment(detection bias) | High |
|  | Incomplete outcome data(attrition bias) | Low |
|  | Selective reporting(reporting bias) | Low |
|  | Other bias | High |
| Faghihzadeh et al,2014 | Random sequence generation(selection bias) | Unclear |
|  | Allocation concealment(selection bias) | Low |
|  | Blinding of participants and personnel (performance bias) | Low |
|  | Blinding of outcome assessment(detection bias) | Low |
|  | Incomplete outcome data(attrition bias) | Low |
|  | Selective reporting(reporting bias) | Low |
|  | Other bias | Low |
| Farhangi et al,2014 | Random sequence generation(selection bias) | Unclear |
|  | Allocation concealment(selection bias) | Unclear |
|  | Blinding of participants and personnel (performance bias) | Low |
|  | Blinding of outcome assessment(detection bias) | Low |
|  | Incomplete outcome data(attrition bias) | Unclear |
|  | Selective reporting(reporting bias) | Low |
|  | Other bias | Low |
| Han et al,2014 | Random sequence generation(selection bias) | Unclear |
|  | Allocation concealment(selection bias) | Unclear |
|  | Blinding of participants and personnel (performance bias) | High |
|  | Blinding of outcome assessment(detection bias) | High |
|  | Incomplete outcome data(attrition bias) | Unclear |
|  | Selective reporting(reporting bias) | Low |
|  | Other bias | Low |
| Mohammadshahi et al,2014 | Random sequence generation(selection bias) | Unclear |
|  | Allocation concealment(selection bias) | Unclear |
|  | Blinding of participants and personnel (performance bias) | Low |
|  | Blinding of outcome assessment(detection bias) | Low |
|  | Incomplete outcome data(attrition bias) | Low |
|  | Selective reporting(reporting bias) | Low |
|  | Other bias | Low |
| Sharifi et al,2014 | Random sequence generation(selection bias) | Low |
|  | Allocation concealment(selection bias) | Low |
|  | Blinding of participants and personnel (performance bias) | Low |
|  | Blinding of outcome assessment(detection bias) | Low |
|  | Incomplete outcome data(attrition bias) | Low |
|  | Selective reporting(reporting bias) | Low |
|  | Other bias | Low |
| Solhi et al,2014 | Random sequence generation(selection bias) | Low |
|  | Allocation concealment(selection bias) | Low |
|  | Blinding of participants and personnel (performance bias) | Unclear |
|  | Blinding of outcome assessment(detection bias) | Unclear |
|  | Incomplete outcome data(attrition bias) | Unclear |
|  | Selective reporting(reporting bias) | Low |
|  | Other bias | Low |
| Aller et al,2015 | Random sequence generation(selection bias) | Low |
|  | Allocation concealment(selection bias) | Low |
|  | Blinding of participants and personnel (performance bias) | Unclear |
|  | Blinding of outcome assessment(detection bias) | Unclear |
|  | Incomplete outcome data(attrition bias) | Low |
|  | Selective reporting(reporting bias) | Low |
|  | Other bias | Low |
| Amiri-Moghadam et al,2015 | Random sequence generation(selection bias) | Low |
|  | Allocation concealment(selection bias) | Low |
|  | Blinding of participants and personnel (performance bias) | Low |
|  | Blinding of outcome assessment(detection bias) | Low |
|  | Incomplete outcome data(attrition bias) | Unclear |
|  | Selective reporting(reporting bias) | Low |
|  | Other bias | Low |
| Chen et al,2015 | Random sequence generation(selection bias) | Low |
|  | Allocation concealment(selection bias) | Low |
|  | Blinding of participants and personnel (performance bias) | Low |
|  | Blinding of outcome assessment(detection bias) | Low |
|  | Incomplete outcome data(attrition bias) | Unclear |
|  | Selective reporting(reporting bias) | Low |
|  | Other bias | Low |
| El-Haggar et al,2015 | Random sequence generation(selection bias) | Unclear |
|  | Allocation concealment(selection bias) | Unclear |
|  | Blinding of participants and personnel (performance bias) | Unclear |
|  | Blinding of outcome assessment(detection bias) | Unclear |
|  | Incomplete outcome data(attrition bias) | Low |
|  | Selective reporting(reporting bias) | Low |
|  | Other bias | Low |
| Faghihzadeh et al,2015 | Random sequence generation(selection bias) | Low |
|  | Allocation concealment(selection bias) | Low |
|  | Blinding of participants and personnel (performance bias) | Low |
|  | Blinding of outcome assessment(detection bias) | Low |
|  | Incomplete outcome data(attrition bias) | Low |
|  | Selective reporting(reporting bias) | Low |
|  | Other bias | Low |
| Guy et al,2015 | Random sequence generation(selection bias) | Unclear |
|  | Allocation concealment(selection bias) | Unclear |
|  | Blinding of participants and personnel (performance bias) | Unclear |
|  | Blinding of outcome assessment(detection bias) | Unclear |
|  | Incomplete outcome data(attrition bias) | Low |
|  | Selective reporting(reporting bias) | Low |
|  | Other bias | Low |
| Sorrentino et al,2015 | Random sequence generation(selection bias) | High |
|  | Allocation concealment(selection bias) | High |
|  | Blinding of participants and personnel (performance bias) | High |
|  | Blinding of outcome assessment(detection bias) | High |
|  | Incomplete outcome data(attrition bias) | Low |
|  | Selective reporting(reporting bias) | Low |
|  | Other bias | Low |
| Ebrahimi-Mameghani et al,2016 | Random sequence generation(selection bias) | Low |
|  | Allocation concealment(selection bias) | Low |
|  | Blinding of participants and personnel (performance bias) | Low |
|  | Blinding of outcome assessment(detection bias) | Low |
|  | Incomplete outcome data(attrition bias) | Low |
|  | Selective reporting(reporting bias) | Low |
|  | Other bias | Low |
| Ekhlasi et al,2016 | Random sequence generation(selection bias) | Low |
|  | Allocation concealment(selection bias) | Low |
|  | Blinding of participants and personnel (performance bias) | Low |
|  | Blinding of outcome assessment(detection bias) | Low |
|  | Incomplete outcome data(attrition bias) | Low |
|  | Selective reporting(reporting bias) | Low |
|  | Other bias | Low |
| Federico et al,2016 | Random sequence generation(selection bias) | High |
|  | Allocation concealment(selection bias) | High |
|  | Blinding of participants and personnel (performance bias) | High |
|  | Blinding of outcome assessment(detection bias) | High |
|  | Incomplete outcome data(attrition bias) | Low |
|  | Selective reporting(reporting bias) | Low |
|  | Other bias | Low |
| Heebøll et al,2016 | Random sequence generation(selection bias) | Unclear |
|  | Allocation concealment(selection bias) | Unclear |
|  | Blinding of participants and personnel (performance bias) | Low |
|  | Blinding of outcome assessment(detection bias) | Low |
|  | Incomplete outcome data(attrition bias) | Low |
|  | Selective reporting(reporting bias) | Low |
|  | Other bias | Low |
| Hong et al,2016 | Random sequence generation(selection bias) | Low |
|  | Allocation concealment(selection bias) | Low |
|  | Blinding of participants and personnel (performance bias) | Low |
|  | Blinding of outcome assessment(detection bias) | Low |
|  | Incomplete outcome data(attrition bias) | Unclear |
|  | Selective reporting(reporting bias) | Low |
|  | Other bias | Low |
| Panahi et al,2016 | Random sequence generation(selection bias) | Unclear |
|  | Allocation concealment(selection bias) | Unclear |
|  | Blinding of participants and personnel (performance bias) | Unclear |
|  | Blinding of outcome assessment(detection bias) | Unclear |
|  | Incomplete outcome data(attrition bias) | Low |
|  | Selective reporting(reporting bias) | Low |
|  | Other bias | Low |
| Rahimlou et al,2016 | Random sequence generation(selection bias) | Low |
|  | Allocation concealment(selection bias) | Low |
|  | Blinding of participants and personnel (performance bias) | Low |
|  | Blinding of outcome assessment(detection bias) | Low |
|  | Incomplete outcome data(attrition bias) | Low |
|  | Selective reporting(reporting bias) | Low |
|  | Other bias | Low |
| Rahmani et al,2016 | Random sequence generation(selection bias) | Low |
|  | Allocation concealment(selection bias) | Low |
|  | Blinding of participants and personnel (performance bias) | Low |
|  | Blinding of outcome assessment(detection bias) | Low |
|  | Incomplete outcome data(attrition bias) | Low |
|  | Selective reporting(reporting bias) | Low |
|  | Other bias | Low |
| Rangboo et al,2016 | Random sequence generation(selection bias) | Low |
|  | Allocation concealment(selection bias) | Low |
|  | Blinding of participants and personnel (performance bias) | Low |
|  | Blinding of outcome assessment(detection bias) | Low |
|  | Incomplete outcome data(attrition bias) | Low |
|  | Selective reporting(reporting bias) | Low |
|  | Other bias | Low |
| Ebrahimi-Mameghani et al,2017 | Random sequence generation(selection bias) | Low |
|  | Allocation concealment(selection bias) | Low |
|  | Blinding of participants and personnel (performance bias) | Low |
|  | Blinding of outcome assessment(detection bias) | Low |
|  | Incomplete outcome data(attrition bias) | Low |
|  | Selective reporting(reporting bias) | Low |
|  | Other bias | Low |
| Ghaffari et al,2017 | Random sequence generation(selection bias) | Low |
|  | Allocation concealment(selection bias) | Low |
|  | Blinding of participants and personnel (performance bias) | Low |
|  | Blinding of outcome assessment(detection bias) | Low |
|  | Incomplete outcome data(attrition bias) | Low |
|  | Selective reporting(reporting bias) | Low |
|  | Other bias | Low |
| Navekar et al,2017 | Random sequence generation(selection bias) | Low |
|  | Allocation concealment(selection bias) | Low |
|  | Blinding of participants and personnel (performance bias) | Low |
|  | Blinding of outcome assessment(detection bias) | Low |
|  | Incomplete outcome data(attrition bias) | Low |
|  | Selective reporting(reporting bias) | Low |
|  | Other bias | Low |
| Pakravan et al,2017 | Random sequence generation(selection bias) | Low |
|  | Allocation concealment(selection bias) | Low |
|  | Blinding of participants and personnel (performance bias) | Low |
|  | Blinding of outcome assessment(detection bias) | Low |
|  | Incomplete outcome data(attrition bias) | Low |
|  | Selective reporting(reporting bias) | Low |
|  | Other bias | Low |
| Pakzad et al,2017 | Random sequence generation(selection bias) | Low |
|  | Allocation concealment(selection bias) | Low |
|  | Blinding of participants and personnel (performance bias) | Low |
|  | Blinding of outcome assessment(detection bias) | Low |
|  | Incomplete outcome data(attrition bias) | Low |
|  | Selective reporting(reporting bias) | Low |
|  | Other bias | Low |
| Panahi et al,2017 | Random sequence generation(selection bias) | Low |
|  | Allocation concealment(selection bias) | Low |
|  | Blinding of participants and personnel (performance bias) | Unclear |
|  | Blinding of outcome assessment(detection bias) | Unclear |
|  | Incomplete outcome data(attrition bias) | Low |
|  | Selective reporting(reporting bias) | Low |
|  | Other bias | Low |
| Polyzos et al,2017 | Random sequence generation(selection bias) | Low |
|  | Allocation concealment(selection bias) | Low |
|  | Blinding of participants and personnel (performance bias) | High |
|  | Blinding of outcome assessment(detection bias) | High |
|  | Incomplete outcome data(attrition bias) | Low |
|  | Selective reporting(reporting bias) | Low |
|  | Other bias | Unclear |
| Zöhrer et al,2017 | Random sequence generation(selection bias) | Low |
|  | Allocation concealment(selection bias) | Low |
|  | Blinding of participants and personnel (performance bias) | Low |
|  | Blinding of outcome assessment(detection bias) | Low |
|  | Incomplete outcome data(attrition bias) | Low |
|  | Selective reporting(reporting bias) | Low |
|  | Other bias | Low |
| Amanat et al,2018 | Random sequence generation(selection bias) | Low |
|  | Allocation concealment(selection bias) | Low |
|  | Blinding of participants and personnel (performance bias) | Low |
|  | Blinding of outcome assessment(detection bias) | Low |
|  | Incomplete outcome data(attrition bias) | Low |
|  | Selective reporting(reporting bias) | Low |
|  | Other bias | Low |
| Amirkhizi et al,2018 | Random sequence generation(selection bias) | Low |
|  | Allocation concealment(selection bias) | Low |
|  | Blinding of participants and personnel (performance bias) | Low |
|  | Blinding of outcome assessment(detection bias) | Low |
|  | Incomplete outcome data(attrition bias) | Low |
|  | Selective reporting(reporting bias) | Low |
|  | Other bias | Low |
| Asghari et al,2018 | Random sequence generation(selection bias) | Low |
|  | Allocation concealment(selection bias) | Low |
|  | Blinding of participants and personnel (performance bias) | Low |
|  | Blinding of outcome assessment(detection bias) | Low |
|  | Incomplete outcome data(attrition bias) | Low |
|  | Selective reporting(reporting bias) | Low |
|  | Other bias | Low |
| Ghaffari et al,2018 | Random sequence generation(selection bias) | Low |
|  | Allocation concealment(selection bias) | Low |
|  | Blinding of participants and personnel (performance bias) | Low |
|  | Blinding of outcome assessment(detection bias) | Low |
|  | Incomplete outcome data(attrition bias) | Low |
|  | Selective reporting(reporting bias) | Low |
|  | Other bias | Low |
| Kantartzis et al,2018 | Random sequence generation(selection bias) | Unclear |
|  | Allocation concealment(selection bias) | Unclear |
|  | Blinding of participants and personnel (performance bias) | Low |
|  | Blinding of outcome assessment(detection bias) | Low |
|  | Incomplete outcome data(attrition bias) | Low |
|  | Selective reporting(reporting bias) | Low |
|  | Other bias | Low |
| Hosseinpour-Arjmand et al,2018 | Random sequence generation(selection bias) | Low |
|  | Allocation concealment(selection bias) | Low |
|  | Blinding of participants and personnel (performance bias) | Low |
|  | Blinding of outcome assessment(detection bias) | Low |
|  | Incomplete outcome data(attrition bias) | Low |
|  | Selective reporting(reporting bias) | Low |
|  | Other bias | Low |
| Pervez et al,2018 | Random sequence generation(selection bias) | Unclear |
|  | Allocation concealment(selection bias) | Unclear |
|  | Blinding of participants and personnel (performance bias) | Low |
|  | Blinding of outcome assessment(detection bias) | Low |
|  | Incomplete outcome data(attrition bias) | Low |
|  | Selective reporting(reporting bias) | Low |
|  | Other bias | Low |
| Saadati et al,2018 | Random sequence generation(selection bias) | Low |
|  | Allocation concealment(selection bias) | Low |
|  | Blinding of participants and personnel (performance bias) | Unclear |
|  | Blinding of outcome assessment(detection bias) | Unclear |
|  | Incomplete outcome data(attrition bias) | Low |
|  | Selective reporting(reporting bias) | Low |
|  | Other bias | Low |
| Anushiravani et al,2019 | Random sequence generation(selection bias) | Low |
|  | Allocation concealment(selection bias) | Low |
|  | Blinding of participants and personnel (performance bias) | Low |
|  | Blinding of outcome assessment(detection bias) | Low |
|  | Incomplete outcome data(attrition bias) | Low |
|  | Selective reporting(reporting bias) | Low |
|  | Other bias | Low |
| Bril et al,2019 | Random sequence generation(selection bias) | Low |
|  | Allocation concealment(selection bias) | Low |
|  | Blinding of participants and personnel (performance bias) | Low |
|  | Blinding of outcome assessment(detection bias) | Low |
|  | Incomplete outcome data(attrition bias) | Low |
|  | Selective reporting(reporting bias) | Low |
|  | Other bias | Low |
| Chashmniam et al,2019 | Random sequence generation(selection bias) | Low |
|  | Allocation concealment(selection bias) | Low |
|  | Blinding of participants and personnel (performance bias) | Low |
|  | Blinding of outcome assessment(detection bias) | Low |
|  | Incomplete outcome data(attrition bias) | Low |
|  | Selective reporting(reporting bias) | Low |
|  | Other bias | Low |
| Cheraghpour et al,2019 | Random sequence generation(selection bias) | Low |
|  | Allocation concealment(selection bias) | Low |
|  | Blinding of participants and personnel (performance bias) | Low |
|  | Blinding of outcome assessment(detection bias) | Low |
|  | Incomplete outcome data(attrition bias) | Low |
|  | Selective reporting(reporting bias) | Low |
|  | Other bias | Low |
| Hosseinpour-Arjmand et al,2019 | Random sequence generation(selection bias) | Low |
|  | Allocation concealment(selection bias) | Low |
|  | Blinding of participants and personnel (performance bias) | Low |
|  | Blinding of outcome assessment(detection bias) | Low |
|  | Incomplete outcome data(attrition bias) | Low |
|  | Selective reporting(reporting bias) | Low |
|  | Other bias | Low |
| Jazayeri-Tehrani et al,2019 | Random sequence generation(selection bias) | Low |
|  | Allocation concealment(selection bias) | Low |
|  | Blinding of participants and personnel (performance bias) | Low |
|  | Blinding of outcome assessment(detection bias) | Low |
|  | Incomplete outcome data(attrition bias) | Low |
|  | Selective reporting(reporting bias) | Low |
|  | Other bias | Low |
| Mirhafez et al,2019 | Random sequence generation(selection bias) | Low |
|  | Allocation concealment(selection bias) | Low |
|  | Blinding of participants and personnel (performance bias) | Low |
|  | Blinding of outcome assessment(detection bias) | Low |
|  | Incomplete outcome data(attrition bias) | Low |
|  | Selective reporting(reporting bias) | Low |
|  | Other bias | Low |
| Nobili et al,2019 | Random sequence generation(selection bias) | Unclear |
|  | Allocation concealment(selection bias) | Unclear |
|  | Blinding of participants and personnel (performance bias) | Low |
|  | Blinding of outcome assessment(detection bias) | Low |
|  | Incomplete outcome data(attrition bias) | Low |
|  | Selective reporting(reporting bias) | Low |
|  | Other bias | Low |
| Oliveira et al,2019 | Random sequence generation(selection bias) | Unclear |
|  | Allocation concealment(selection bias) | Unclear |
|  | Blinding of participants and personnel (performance bias) | High |
|  | Blinding of outcome assessment(detection bias) | High |
|  | Incomplete outcome data(attrition bias) | Low |
|  | Selective reporting(reporting bias) | Low |
|  | Other bias | Unclear |
| Panahi et al,2019 | Random sequence generation(selection bias) | Unclear |
|  | Allocation concealment(selection bias) | Unclear |
|  | Blinding of participants and personnel (performance bias) | Unclear |
|  | Blinding of outcome assessment(detection bias) | Unclear |
|  | Incomplete outcome data(attrition bias) | Low |
|  | Selective reporting(reporting bias) | Low |
|  | Other bias | Low |
| Rahmanabadi et al,2019 | Random sequence generation(selection bias) | Low |
|  | Allocation concealment(selection bias) | Low |
|  | Blinding of participants and personnel (performance bias) | Low |
|  | Blinding of outcome assessment(detection bias) | Low |
|  | Incomplete outcome data(attrition bias) | Low |
|  | Selective reporting(reporting bias) | Low |
|  | Other bias | Low |
| Rashidmayvan et al,2019 | Random sequence generation(selection bias) | Unclear |
|  | Allocation concealment(selection bias) | Unclear |
|  | Blinding of participants and personnel (performance bias) | Low |
|  | Blinding of outcome assessment(detection bias) | Low |
|  | Incomplete outcome data(attrition bias) | Low |
|  | Selective reporting(reporting bias) | Low |
|  | Other bias | Low |
| Saadati et al,2019 | Random sequence generation(selection bias) | Low |
|  | Allocation concealment(selection bias) | Low |
|  | Blinding of participants and personnel (performance bias) | Low |
|  | Blinding of outcome assessment(detection bias) | Low |
|  | Incomplete outcome data(attrition bias) | Low |
|  | Selective reporting(reporting bias) | Low |
|  | Other bias | Low |
| Saadati et al,2019 | Random sequence generation(selection bias) | Low |
|  | Allocation concealment(selection bias) | Low |
|  | Blinding of participants and personnel (performance bias) | Low |
|  | Blinding of outcome assessment(detection bias) | Low |
|  | Incomplete outcome data(attrition bias) | Low |
|  | Selective reporting(reporting bias) | Low |
|  | Other bias | Low |
| Torquato et al,2019 | Random sequence generation(selection bias) | Unclear |
|  | Allocation concealment(selection bias) | Unclear |
|  | Blinding of participants and personnel (performance bias) | Unclear |
|  | Blinding of outcome assessment(detection bias) | Unclear |
|  | Incomplete outcome data(attrition bias) | Unclear |
|  | Selective reporting(reporting bias) | Unclear |
|  | Other bias | Unclear |
| Bahrami et al,2020 | Random sequence generation(selection bias) | Low |
|  | Allocation concealment(selection bias) | Low |
|  | Blinding of participants and personnel (performance bias) | Low |
|  | Blinding of outcome assessment(detection bias) | Low |
|  | Incomplete outcome data(attrition bias) | Low |
|  | Selective reporting(reporting bias) | Low |
|  | Other bias | Low |
| Cerletti et al,2020 | Random sequence generation(selection bias) | Unclear |
|  | Allocation concealment(selection bias) | Unclear |
|  | Blinding of participants and personnel (performance bias) | Low |
|  | Blinding of outcome assessment(detection bias) | Low |
|  | Incomplete outcome data(attrition bias) | Low |
|  | Selective reporting(reporting bias) | Low |
|  | Other bias | Low |
| Farzin et al,2020 | Random sequence generation(selection bias) | Low |
|  | Allocation concealment(selection bias) | Low |
|  | Blinding of participants and personnel (performance bias) | Low |
|  | Blinding of outcome assessment(detection bias) | Low |
|  | Incomplete outcome data(attrition bias) | Low |
|  | Selective reporting(reporting bias) | Low |
|  | Other bias | Low |
| Fathi et al,2020 | Random sequence generation(selection bias) | Low |
|  | Allocation concealment(selection bias) | Low |
|  | Blinding of participants and personnel (performance bias) | Low |
|  | Blinding of outcome assessment(detection bias) | Low |
|  | Incomplete outcome data(attrition bias) | Low |
|  | Selective reporting(reporting bias) | Low |
|  | Other bias | Low |
| Fathi et al,2020 | Random sequence generation(selection bias) | Low |
|  | Allocation concealment(selection bias) | Low |
|  | Blinding of participants and personnel (performance bias) | Low |
|  | Blinding of outcome assessment(detection bias) | Low |
|  | Incomplete outcome data(attrition bias) | Low |
|  | Selective reporting(reporting bias) | Low |
|  | Other bias | Low |
| Ferro et al,2020 | Random sequence generation(selection bias) | Low |
|  | Allocation concealment(selection bias) | Low |
|  | Blinding of participants and personnel (performance bias) | Low |
|  | Blinding of outcome assessment(detection bias) | Low |
|  | Incomplete outcome data(attrition bias) | Low |
|  | Selective reporting(reporting bias) | Low |
|  | Other bias | Low |
| Hariri et al,2020 | Random sequence generation(selection bias) | Low |
|  | Allocation concealment(selection bias) | Low |
|  | Blinding of participants and personnel (performance bias) | Low |
|  | Blinding of outcome assessment(detection bias) | Low |
|  | Incomplete outcome data(attrition bias) | Low |
|  | Selective reporting(reporting bias) | Low |
|  | Other bias | Low |
| Moradi Kelardeh et al,2020 | Random sequence generation(selection bias) | Unclear |
|  | Allocation concealment(selection bias) | Unclear |
|  | Blinding of participants and personnel (performance bias) | Low |
|  | Blinding of outcome assessment(detection bias) | Low |
|  | Incomplete outcome data(attrition bias) | Low |
|  | Selective reporting(reporting bias) | Low |
|  | Other bias | Low |
| Pervez et al,2020 | Random sequence generation(selection bias) | Low |
|  | Allocation concealment(selection bias) | Low |
|  | Blinding of participants and personnel (performance bias) | Low |
|  | Blinding of outcome assessment(detection bias) | Low |
|  | Incomplete outcome data(attrition bias) | Low |
|  | Selective reporting(reporting bias) | Low |
|  | Other bias | Low |
| Pour et al,2020 | Random sequence generation(selection bias) | Low |
|  | Allocation concealment(selection bias) | Low |
|  | Blinding of participants and personnel (performance bias) | Low |
|  | Blinding of outcome assessment(detection bias) | Low |
|  | Incomplete outcome data(attrition bias) | Low |
|  | Selective reporting(reporting bias) | Low |
|  | Other bias | Low |
| Rafie et al,2020 | Random sequence generation(selection bias) | Low |
|  | Allocation concealment(selection bias) | Low |
|  | Blinding of participants and personnel (performance bias) | Low |
|  | Blinding of outcome assessment(detection bias) | Low |
|  | Incomplete outcome data(attrition bias) | Low |
|  | Selective reporting(reporting bias) | Low |
|  | Other bias | Low |
| Saberi-Karimian et al,2020 | Random sequence generation(selection bias) | Unclear |
|  | Allocation concealment(selection bias) | Unclear |
|  | Blinding of participants and personnel (performance bias) | Low |
|  | Blinding of outcome assessment(detection bias) | Low |
|  | Incomplete outcome data(attrition bias) | Low |
|  | Selective reporting(reporting bias) | Low |
|  | Other bias | Low |
| Sangouni et al,2020 | Random sequence generation(selection bias) | Low |
|  | Allocation concealment(selection bias) | Low |
|  | Blinding of participants and personnel (performance bias) | Low |
|  | Blinding of outcome assessment(detection bias) | Low |
|  | Incomplete outcome data(attrition bias) | Low |
|  | Selective reporting(reporting bias) | Low |
|  | Other bias | Low |
| Amerikanou et al,2021 | Random sequence generation(selection bias) | Low |
|  | Allocation concealment(selection bias) | Low |
|  | Blinding of participants and personnel (performance bias) | Unclear |
|  | Blinding of outcome assessment(detection bias) | Unclear |
|  | Incomplete outcome data(attrition bias) | Low |
|  | Selective reporting(reporting bias) | Low |
|  | Other bias | Low |
| Darvish Damavandi et al,2021 | Random sequence generation(selection bias) | Low |
|  | Allocation concealment(selection bias) | Low |
|  | Blinding of participants and personnel (performance bias) | Low |
|  | Blinding of outcome assessment(detection bias) | Low |
|  | Incomplete outcome data(attrition bias) | Low |
|  | Selective reporting(reporting bias) | Low |
|  | Other bias | Low |
| Domech et al,2021 | Random sequence generation(selection bias) | Unclear |
|  | Allocation concealment(selection bias) | Unclear |
|  | Blinding of participants and personnel (performance bias) | Low |
|  | Blinding of outcome assessment(detection bias) | Low |
|  | Incomplete outcome data(attrition bias) | Low |
|  | Selective reporting(reporting bias) | Low |
|  | Other bias | Low |
| Fouda et al,2021 | Random sequence generation(selection bias) | Low |
|  | Allocation concealment(selection bias) | Low |
|  | Blinding of participants and personnel (performance bias) | Low |
|  | Blinding of outcome assessment(detection bias) | Low |
|  | Incomplete outcome data(attrition bias) | Low |
|  | Selective reporting(reporting bias) | Low |
|  | Other bias | Low |
| Harrison et al,2021 | Random sequence generation(selection bias) | Unclear |
|  | Allocation concealment(selection bias) | Unclear |
|  | Blinding of participants and personnel (performance bias) | Low |
|  | Blinding of outcome assessment(detection bias) | Low |
|  | Incomplete outcome data(attrition bias) | Low |
|  | Selective reporting(reporting bias) | Low |
|  | Other bias | Low |
| He et al,2021 | Random sequence generation(selection bias) | Low |
|  | Allocation concealment(selection bias) | Low |
|  | Blinding of participants and personnel (performance bias) | Low |
|  | Blinding of outcome assessment(detection bias) | Low |
|  | Incomplete outcome data(attrition bias) | Low |
|  | Selective reporting(reporting bias) | Low |
|  | Other bias | Low |
| Jarhahzadeh et al,2021 | Random sequence generation(selection bias) | Unclear |
|  | Allocation concealment(selection bias) | Unclear |
|  | Blinding of participants and personnel (performance bias) | Low |
|  | Blinding of outcome assessment(detection bias) | Low |
|  | Incomplete outcome data(attrition bias) | Low |
|  | Selective reporting(reporting bias) | Low |
|  | Other bias | Low |
| Kedarisetty et al,2021 | Random sequence generation(selection bias) | Low |
|  | Allocation concealment(selection bias) | Low |
|  | Blinding of participants and personnel (performance bias) | High |
|  | Blinding of outcome assessment(detection bias) | High |
|  | Incomplete outcome data(attrition bias) | Low |
|  | Selective reporting(reporting bias) | Low |
|  | Other bias | Low |
| Kooshki et al,2021 | Random sequence generation(selection bias) | Low |
|  | Allocation concealment(selection bias) | Low |
|  | Blinding of participants and personnel (performance bias) | Low |
|  | Blinding of outcome assessment(detection bias) | Low |
|  | Incomplete outcome data(attrition bias) | Low |
|  | Selective reporting(reporting bias) | Low |
|  | Other bias | Low |
| Majnooni et al,2021 | Random sequence generation(selection bias) | Unclear |
|  | Allocation concealment(selection bias) | Unclear |
|  | Blinding of participants and personnel (performance bias) | Unclear |
|  | Blinding of outcome assessment(detection bias) | Unclear |
|  | Incomplete outcome data(attrition bias) | Low |
|  | Selective reporting(reporting bias) | Low |
|  | Other bias | Low |
| Mirhafez et al,2021 | Random sequence generation(selection bias) | Low |
|  | Allocation concealment(selection bias) | Low |
|  | Blinding of participants and personnel (performance bias) | Low |
|  | Blinding of outcome assessment(detection bias) | Low |
|  | Incomplete outcome data(attrition bias) | Low |
|  | Selective reporting(reporting bias) | Low |
|  | Other bias | Low |
| Namkhah et al,2021 | Random sequence generation(selection bias) | Unclear |
|  | Allocation concealment(selection bias) | Unclear |
|  | Blinding of participants and personnel (performance bias) | Low |
|  | Blinding of outcome assessment(detection bias) | Low |
|  | Incomplete outcome data(attrition bias) | Low |
|  | Selective reporting(reporting bias) | Low |
|  | Other bias | Low |
| Yari et al,2021 | Random sequence generation(selection bias) | Low |
|  | Allocation concealment(selection bias) | Low |
|  | Blinding of participants and personnel (performance bias) | High |
|  | Blinding of outcome assessment(detection bias) | High |
|  | Incomplete outcome data(attrition bias) | Unclear |
|  | Selective reporting(reporting bias) | Low |
|  | Other bias | Unclear |
| Akbari et al,2022 | Random sequence generation(selection bias) | Low |
|  | Allocation concealment(selection bias) | Low |
|  | Blinding of participants and personnel (performance bias) | Low |
|  | Blinding of outcome assessment(detection bias) | Low |
|  | Incomplete outcome data(attrition bias) | Low |
|  | Selective reporting(reporting bias) | Low |
|  | Other bias | Low |
| Arefhosseini et al,2022 | Random sequence generation(selection bias) | Low |
|  | Allocation concealment(selection bias) | Low |
|  | Blinding of participants and personnel (performance bias) | Low |
|  | Blinding of outcome assessment(detection bias) | Low |
|  | Incomplete outcome data(attrition bias) | Low |
|  | Selective reporting(reporting bias) | Low |
|  | Other bias | Low |
| Atarodi et al,2022 | Random sequence generation(selection bias) | Low |
|  | Allocation concealment(selection bias) | Low |
|  | Blinding of participants and personnel (performance bias) | Low |
|  | Blinding of outcome assessment(detection bias) | Low |
|  | Incomplete outcome data(attrition bias) | Low |
|  | Selective reporting(reporting bias) | Low |
|  | Other bias | Low |
| Ferro et al,2022 | Random sequence generation(selection bias) | Unclear |
|  | Allocation concealment(selection bias) | Unclear |
|  | Blinding of participants and personnel (performance bias) | Low |
|  | Blinding of outcome assessment(detection bias) | Low |
|  | Incomplete outcome data(attrition bias) | Low |
|  | Selective reporting(reporting bias) | Low |
|  | Other bias | Low |
| Hosseinian et al,2022 | Random sequence generation(selection bias) | Low |
|  | Allocation concealment(selection bias) | Low |
|  | Blinding of participants and personnel (performance bias) | Low |
|  | Blinding of outcome assessment(detection bias) | Low |
|  | Incomplete outcome data(attrition bias) | Low |
|  | Selective reporting(reporting bias) | Low |
|  | Other bias | Low |
| Kalhori et al,2022 | Random sequence generation(selection bias) | Low |
|  | Allocation concealment(selection bias) |  |
|  | Blinding of participants and personnel (performance bias) | Low |
|  | Blinding of outcome assessment(detection bias) | Low |
|  | Incomplete outcome data(attrition bias) | Low |
|  | Selective reporting(reporting bias) | Low |
|  | Other bias | Low |
| Mohammadi et al,2022 | Random sequence generation(selection bias) | Low |
|  | Allocation concealment(selection bias) | Low |
|  | Blinding of participants and personnel (performance bias) | Low |
|  | Blinding of outcome assessment(detection bias) | Low |
|  | Incomplete outcome data(attrition bias) | Low |
|  | Selective reporting(reporting bias) | Low |
|  | Other bias | Low |
| Naeini et al,2022 | Random sequence generation(selection bias) | Low |
|  | Allocation concealment(selection bias) | Low |
|  | Blinding of participants and personnel (performance bias) | Low |
|  | Blinding of outcome assessment(detection bias) | Low |
|  | Incomplete outcome data(attrition bias) | Low |
|  | Selective reporting(reporting bias) | Low |
|  | Other bias | Low |
| Pervez et al,2022 | Random sequence generation(selection bias) | Low |
|  | Allocation concealment(selection bias) | Low |
|  | Blinding of participants and personnel (performance bias) | Low |
|  | Blinding of outcome assessment(detection bias) | Low |
|  | Incomplete outcome data(attrition bias) | Low |
|  | Selective reporting(reporting bias) | Low |
|  | Other bias | Low |
| Rashidmayvan et al,2022 | Random sequence generation(selection bias) | Low |
|  | Allocation concealment(selection bias) | Low |
|  | Blinding of participants and personnel (performance bias) | Low |
|  | Blinding of outcome assessment(detection bias) | Low |
|  | Incomplete outcome data(attrition bias) | Low |
|  | Selective reporting(reporting bias) | Low |
|  | Other bias | Low |
| Rostamizadeh et al,2022 | Random sequence generation(selection bias) | Low |
|  | Allocation concealment(selection bias) | Low |
|  | Blinding of participants and personnel (performance bias) | Low |
|  | Blinding of outcome assessment(detection bias) | Low |
|  | Incomplete outcome data(attrition bias) | Low |
|  | Selective reporting(reporting bias) | Low |
|  | Other bias | Low |
| Wang et al,2022 | Random sequence generation(selection bias) | Unclear |
|  | Allocation concealment(selection bias) | Unclear |
|  | Blinding of participants and personnel (performance bias) | Unclear |
|  | Blinding of outcome assessment(detection bias) | Unclear |
|  | Incomplete outcome data(attrition bias) | Low |
|  | Selective reporting(reporting bias) | Low |
|  | Other bias | Low |
| Xie et al,2022 | Random sequence generation(selection bias) | Unclear |
|  | Allocation concealment(selection bias) | Unclear |
|  | Blinding of participants and personnel (performance bias) | Unclear |
|  | Blinding of outcome assessment(detection bias) | Unclear |
|  | Incomplete outcome data(attrition bias) | Low |
|  | Selective reporting(reporting bias) | Low |
|  | Other bias | Low |
| Zojaji et al,2022 | Random sequence generation(selection bias) | Unclear |
|  | Allocation concealment(selection bias) | Unclear |
|  | Blinding of participants and personnel (performance bias) | Low |
|  | Blinding of outcome assessment(detection bias) | Low |
|  | Incomplete outcome data(attrition bias) | Low |
|  | Selective reporting(reporting bias) | Low |
|  | Other bias | Low |
| Beheshti Namdar et al,2023 | Random sequence generation(selection bias) | Low |
|  | Allocation concealment(selection bias) | Low |
|  | Blinding of participants and personnel (performance bias) | Low |
|  | Blinding of outcome assessment(detection bias) | Low |
|  | Incomplete outcome data(attrition bias) | Low |
|  | Selective reporting(reporting bias) | Low |
|  | Other bias | Low |
| Damavandi et al,2023 | Random sequence generation(selection bias) | Low |
|  | Allocation concealment(selection bias) | Low |
|  | Blinding of participants and personnel (performance bias) | Low |
|  | Blinding of outcome assessment(detection bias) | Low |
|  | Incomplete outcome data(attrition bias) | Low |
|  | Selective reporting(reporting bias) | Low |
|  | Other bias | Low |
| Ghoreishi et al,2023 | Random sequence generation(selection bias) | Low |
|  | Allocation concealment(selection bias) | Low |
|  | Blinding of participants and personnel (performance bias) | Low |
|  | Blinding of outcome assessment(detection bias) | Low |
|  | Incomplete outcome data(attrition bias) | Low |
|  | Selective reporting(reporting bias) | Low |
|  | Other bias | Low |
| Khoshbaten et al,2023 | Random sequence generation(selection bias) | Unclear |
|  | Allocation concealment(selection bias) | Unclear |
|  | Blinding of participants and personnel (performance bias) | Unclear Unclear |
|  | Blinding of outcome assessment(detection bias) | Unclear |
|  | Incomplete outcome data(attrition bias) | Low |
|  | Selective reporting(reporting bias) | Low |
|  | Other bias | Low |
| Majeed et al,2023 | Random sequence generation(selection bias) | Low |
|  | Allocation concealment(selection bias) | Low |
|  | Blinding of participants and personnel (performance bias) | Low |
|  | Blinding of outcome assessment(detection bias) | Low |
|  | Incomplete outcome data(attrition bias) | Low |
|  | Selective reporting(reporting bias) | Low |
|  | Other bias | Low |
| Mojtahedi et al,2023 | Random sequence generation(selection bias) | Low |
|  | Allocation concealment(selection bias) | Low |
|  | Blinding of participants and personnel (performance bias) | Low |
|  | Blinding of outcome assessment(detection bias) | Low |
|  | Incomplete outcome data(attrition bias) | Low |
|  | Selective reporting(reporting bias) | Low |
|  | Other bias | Low |
| Rezaei et al,2023 | Random sequence generation(selection bias) | Low |
|  | Allocation concealment(selection bias) | Low |
|  | Blinding of participants and personnel (performance bias) | Low |
|  | Blinding of outcome assessment(detection bias) | Low |
|  | Incomplete outcome data(attrition bias) | Low |
|  | Selective reporting(reporting bias) | Low |
|  | Other bias | Low |
| Safari et al,2023 | Random sequence generation(selection bias) | Low |
|  | Allocation concealment(selection bias) | Low |
|  | Blinding of participants and personnel (performance bias) | Low |
|  | Blinding of outcome assessment(detection bias) | Low |
|  | Incomplete outcome data(attrition bias) | Low |
|  | Selective reporting(reporting bias) | Low |
|  | Other bias | Low |
| Sharifi et al,2023 | Random sequence generation(selection bias) | Low |
|  | Allocation concealment(selection bias) | Low |
|  | Blinding of participants and personnel (performance bias) | Low |
|  | Blinding of outcome assessment(detection bias) | Low |
|  | Incomplete outcome data(attrition bias) | Low |
|  | Selective reporting(reporting bias) | Low |
|  | Other bias | Low |
| Tutunchi et al,2023 | Random sequence generation(selection bias) | Low |
|  | Allocation concealment(selection bias) | Low |
|  | Blinding of participants and personnel (performance bias) | Low |
|  | Blinding of outcome assessment(detection bias) | Low |
|  | Incomplete outcome data(attrition bias) | Low |
|  | Selective reporting(reporting bias) | Low |
|  | Other bias | Low |
| Tutunchi et al,2023 | Random sequence generation(selection bias) | Low |
|  | Allocation concealment(selection bias) | Low |
|  | Blinding of participants and personnel (performance bias) | Low |
|  | Blinding of outcome assessment(detection bias) | Low |
|  | Incomplete outcome data(attrition bias) | Low |
|  | Selective reporting(reporting bias) | Low |
|  | Other bias | Low |

**Table S4.** League table for TG

|  | A | B | C | D | E | F | G | H | I | J | K | M | N | O | P | placebo | Q | R |
| --- | --- | --- | --- | --- | --- | --- | --- | --- | --- | --- | --- | --- | --- | --- | --- | --- | --- | --- |
| A | A | -5 (-24, 14) | -8 (-31, 16) | -7 (-25, 11) | 2 (-16, 20) | 5 (-23, 34) | -5 (-28, 17) | 2 (-27, 31) | -12 (-47, 23) | 11 (-9, 30) | 8 (-27, 43) | 2 (-67, 70) | -5 (-73, 62) | -14 (-54, 27) | -22 (-52, 8) | 10 (-4, 24) | -25 (-76, 27) | 3 (-32, 38) |
| B | 5 (-14, 24) | B | -3 (-24, 18) | -1 (-19, 16) | 7 (-11, 26) | 11 (-19, 40) | 0 (-23, 22) | 7 (-23, 37) | -7 (-39, 25) | 16 (-6, 38) | 13 (-26, 51) | 7 (-61, 75) | 0 (-68, 67) | -9 (-49, 32) | -16 (-45, 11) | 15 (0, 29) | -19 (-70, 30) | 9 (-28, 44) |
| C | 8 (-16, 31) | 3 (-18, 24) | C | 1 (-19, 21) | 10 (-13, 33) | 13 (-19, 46) | 2 (-25, 30) | 10 (-23, 43) | -4 (-37, 28) | 18 (-7, 44) | 15 (-25, 56) | 9 (-58, 77) | 2 (-65, 69) | -6 (-45, 34) | -14 (-43, 15) | 17 (-2, 37) | -17 (-67, 33) | 11 (-27, 49) |
| D | 7 (-11, 25) | 1 (-16, 19) | -1 (-21, 19) | D | 9 (-8, 26) | 12 (-17, 42) | 1 (-22, 25) | 9 (-20, 38) | -6 (-39, 28) | 17 (-4, 40) | 14 (-24, 53) | 8 (-59, 76) | 1 (-66, 68) | -7 (-47, 33) | -15 (-45, 14) | 16 (5, 28) | -18 (-68, 33) | 10 (-25, 45) |
| E | -2 (-20, 16) | -7 (-26, 11) | -10 (-33, 13) | -9 (-26, 8) | E | 3 (-24, 30) | -8 (-31, 16) | 0 (-26, 27) | -14 (-49, 20) | 8 (-13, 30) | 5 (-33, 44) | -1 (-69, 67) | -8 (-75, 59) | -16 (-56, 24) | -24 (-55, 6) | 7 (-5, 20) | -27 (-79, 25) | 1 (-32, 34) |
| F | -5 (-34, 23) | -11 (-40, 19) | -13 (-46, 19) | -12 (-42, 17) | -3 (-30, 24) | F | -11 (-43, 21) | -3 (-34, 27) | -18 (-59, 23) | 5 (-21, 32) | 2 (-41, 45) | -4 (-76, 68) | -11 (-82, 60) | -19 (-66, 27) | -27 (-64, 9) | 4 (-23, 31) | -30 (-86, 26) | -2 (-36, 31) |
| G | 5 (-17, 28) | 0 (-22, 23) | -2 (-30, 25) | -1 (-25, 22) | 8 (-16, 31) | 11 (-21, 43) | G | 8 (-25, 39) | -7 (-44, 30) | 16 (-9, 41) | 13 (-27, 53) | 7 (-63, 77) | 0 (-69, 68) | -8 (-51, 35) | -16 (-50, 17) | 15 (-6, 36) | -19 (-73, 34) | 9 (-30, 46) |
| H | -2 (-31, 27) | -7 (-37, 23) | -10 (-43, 23) | -9 (-38, 20) | 0 (-27, 26) | 3 (-27, 34) | -8 (-39, 25) | H | -14 (-56, 28) | 8 (-22, 39) | 5 (-39, 49) | -1 (-72, 71) | -8 (-78, 63) | -16 (-63, 31) | -24 (-62, 14) | 7 (-20, 35) | -27 (-83, 30) | 1 (-32, 35) |
| I | 12 (-23, 47) | 7 (-25, 39) | 4 (-28, 37) | 6 (-28, 39) | 14 (-20, 49) | 18 (-23, 59) | 7 (-30, 44) | 14 (-28, 56) | I | 23 (-13, 59) | 20 (-28, 68) | 14 (-60, 87) | 7 (-66, 79) | -2 (-51, 48) | -9 (-44, 24) | 22 (-11, 55) | -12 (-50, 25) | 15 (-31, 62) |
| J | -11 (-30, 9) | -16 (-38, 6) | -18 (-44, 7) | -17 (-40, 4) | -8 (-30, 13) | -5 (-32, 21) | -16 (-41, 9) | -8 (-39, 22) | -23 (-59, 13) | J | -3 (-39, 32) | -9 (-79, 60) | -16 (-85, 52) | -25 (-67, 18) | -32 (-62, -3) | -1 (-21, 18) | -35 (-88, 17) | -7 (-44, 28) |
| K | -8 (-43, 27) | -13 (-51, 26) | -15 (-56, 25) | -14 (-53, 24) | -5 (-44, 33) | -2 (-45, 41) | -13 (-53, 27) | -5 (-49, 39) | -20 (-68, 28) | 3 (-32, 39) | K | -6 (-82, 70) | -13 (-88, 62) | -21 (-74, 31) | -29 (-74, 14) | 2 (-34, 39) | -32 (-94, 29) | -4 (-52, 44) |
| M | -2 (-70, 67) | -7 (-75, 61) | -9 (-77, 58) | -8 (-76, 59) | 1 (-67, 69) | 4 (-68, 76) | -7 (-77, 63) | 1 (-71, 72) | -14 (-87, 60) | 9 (-60, 79) | 6 (-70, 82) | M | -7 (-68, 53) | -15 (-70, 39) | -23 (-96, 49) | 8 (-59, 75) | -26 (-109, 57) | 2 (-73, 76) |
| N | 5 (-62, 73) | 0 (-67, 68) | -2 (-69, 65) | -1 (-68, 66) | 8 (-59, 75) | 11 (-60, 82) | 0 (-68, 69) | 8 (-63, 78) | -7 (-79, 66) | 16 (-52, 85) | 13 (-62, 88) | 7 (-53, 68) | N | -8 (-62, 45) | -16 (-87, 54) | 15 (-50, 81) | -19 (-101, 63) | 9 (-65, 83) |
| O | 14 (-27, 54) | 9 (-32, 49) | 6 (-34, 45) | 7 (-33, 47) | 16 (-24, 56) | 19 (-27, 66) | 8 (-35, 51) | 16 (-31, 63) | 2 (-48, 51) | 25 (-18, 67) | 21 (-31, 74) | 15 (-39, 70) | 8 (-45, 62) | O | -8 (-55, 38) | 23 (-15, 62) | -11 (-73, 51) | 17 (-34, 68) |
| P | 22 (-8, 52) | 16 (-11, 45) | 14 (-15, 43) | 15 (-14, 45) | 24 (-6, 55) | 27 (-9, 64) | 16 (-17, 50) | 24 (-14, 62) | 9 (-24, 44) | 32 (3, 62) | 29 (-14, 74) | 23 (-49, 96) | 16 (-54, 87) | 8 (-38, 55) | P | 31 (4, 60) | -3 (-53, 48) | 25 (-18, 68) |
| placebo | -10 (-24, 4) | -15 (-29, 0) | -17 (-37, 2) | -16 (-28, -5) | -7 (-20, 5) | -4 (-31, 23) | -15 (-36, 6) | -7 (-35, 20) | -22 (-55, 11) | 1 (-18, 21) | -2 (-39, 34) | -8 (-75, 59) | -15 (-81, 50) | -23 (-62, 15) | -31 (-60, -4) | placebo | -34 (-85, 16) | -6 (-40, 27) |
| Q | 25 (-27, 76) | 19 (-30, 70) | 17 (-33, 67) | 18 (-33, 68) | 27 (-25, 79) | 30 (-26, 86) | 19 (-34, 73) | 27 (-30, 83) | 12 (-25, 50) | 35 (-17, 88) | 32 (-29, 94) | 26 (-57, 109) | 19 (-63, 101) | 11 (-51, 73) | 3 (-48, 53) | 34 (-16, 85) | Q | 28 (-32, 88) |
| R | -3 (-38, 32) | -9 (-44, 28) | -11 (-49, 27) | -10 (-45, 25) | -1 (-34, 32) | 2 (-31, 36) | -9 (-46, 30) | -1 (-35, 32) | -15 (-62, 31) | 7 (-28, 44) | 4 (-44, 52) | -2 (-76, 73) | -9 (-83, 65) | -17 (-68, 34) | -25 (-68, 18) | 6 (-27, 40) | -28 (-88, 32) | R |

**Table S5.** League table for TC

|  | A | B | C | D | E | F | G | H | I | J | K | M | N | O | P | placebo | Q | R |
| --- | --- | --- | --- | --- | --- | --- | --- | --- | --- | --- | --- | --- | --- | --- | --- | --- | --- | --- |
| A | A | 1 (0, 1) | 0 (-1, 1) | 0 (-1, 0) | -8 (-11, 12) | -9 (-13, 11) | -5 (-12, 3) | -8 (-12, 12) | 0 (-1, 0) | -7 (-11, 13) | -4 (-17, 12) | 0 (-14, 14) | -14 (-34, 7) | -5 (-21, 10) | 0 (-1, 1) | 0 (0, 0) | 10 (-1, 20) | -8 (-12, 12) |
| B | -1 (-1, 0) | B | 0 (-1, 0) | -1 (-1, 0) | -8 (-12, 12) | -10 (-13, 10) | -5 (-12, 3) | -9 (-12, 11) | -1 (-1, 0) | -7 (-11, 12) | -4 (-17, 12) | 0 (-14, 13) | -14 (-35, 6) | -6 (-21, 10) | -1 (-1, 0) | 0 (-1, 0) | 9 (-1, 19) | -8 (-12, 11) |
| C | 0 (-1, 1) | 0 (0, 1) | C | -1 (-1, 0) | -8 (-12, 12) | -9 (-13, 11) | -5 (-12, 3) | -8 (-12, 12) | 0 (-1, 0) | -7 (-11, 13) | -4 (-17, 12) | 0 (-14, 13) | -14 (-35, 6) | -5 (-21, 10) | 0 (-1, 0) | 0 (-1, 1) | 9 (-1, 19) | -8 (-12, 12) |
| D | 0 (0, 1) | 1 (0, 1) | 1 (0, 1) | D | -7 (-11, 13) | -9 (-12, 11) | -4 (-11, 4) | -8 (-11, 12) | 0 (-1, 1) | -7 (-10, 13) | -4 (-16, 13) | 0 (-13, 14) | -13 (-34, 7) | -5 (-20, 10) | 0 (-1, 1) | 1 (0, 1) | 10 (0, 20) | -8 (-11, 12) |
| E | 8 (-12, 11) | 8 (-12, 12) | 8 (-12, 12) | 7 (-13, 11) | E | -1 (-2, -1) | 2 (-12, 10) | -1 (-1, 0) | 7 (-13, 11) | 1 (0, 1) | 3 (-10, 15) | 7 (-14, 22) | -7 (-33, 14) | 2 (-21, 18) | 8 (-12, 11) | 8 (-12, 11) | 17 (-4, 28) | 0 (0, 0) |
| F | 9 (-11, 13) | 10 (-10, 13) | 9 (-11, 13) | 9 (-11, 12) | 1 (1, 2) | F | 4 (-11, 11) | 1 (1, 1) | 9 (-11, 12) | 2 (2, 2) | 4 (-9, 17) | 9 (-13, 23) | -5 (-31, 16) | 3 (-19, 19) | 9 (-11, 13) | 9 (-11, 13) | 18 (-2, 29) | 1 (1, 1) |
| G | 5 (-3, 12) | 5 (-3, 12) | 5 (-3, 12) | 4 (-4, 11) | -2 (-10, 12) | -4 (-11, 11) | G | -3 (-11, 12) | 4 (-4, 11) | -2 (-9, 13) | 1 (-13, 16) | 5 (-11, 20) | -9 (-31, 12) | -1 (-18, 16) | 5 (-3, 12) | 5 (-3, 12) | 14 (1, 27) | -3 (-10, 12) |
| H | 8 (-12, 12) | 9 (-11, 12) | 8 (-12, 12) | 8 (-12, 11) | 1 (0, 1) | -1 (-1, -1) | 3 (-12, 11) | H | 8 (-12, 12) | 1 (1, 2) | 3 (-9, 16) | 8 (-14, 22) | -6 (-32, 15) | 2 (-20, 19) | 8 (-12, 12) | 8 (-12, 12) | 17 (-3, 28) | 0 (0, 0) |
| I | 0 (0, 1) | 1 (0, 1) | 0 (0, 1) | 0 (-1, 1) | -7 (-11, 13) | -9 (-12, 11) | -4 (-11, 4) | -8 (-12, 12) | I | -7 (-10, 13) | -4 (-16, 13) | 0 (-13, 14) | -13 (-34, 7) | -5 (-20, 10) | 0 (0, 1) | 1 (0, 1) | 10 (0, 20) | -8 (-11, 12) |
| J | 7 (-13, 11) | 7 (-12, 11) | 7 (-13, 11) | 7 (-13, 10) | -1 (-1, 0) | -2 (-2, -2) | 2 (-13, 9) | -1 (-2, -1) | 7 (-13, 10) | J | 2 (-11, 14) | 6 (-15, 21) | -7 (-33, 14) | 1 (-21, 17) | 7 (-13, 11) | 7 (-13, 11) | 16 (-4, 27) | -1 (-1, -1) |
| K | 4 (-12, 17) | 4 (-12, 17) | 4 (-12, 17) | 4 (-13, 16) | -3 (-15, 10) | -4 (-17, 9) | -1 (-16, 13) | -3 (-16, 9) | 4 (-13, 16) | -2 (-14, 11) | K | 4 (-17, 23) | -10 (-36, 14) | -1 (-23, 18) | 4 (-12, 16) | 4 (-12, 17) | 14 (-5, 30) | -3 (-15, 10) |
| M | 0 (-14, 14) | 0 (-13, 14) | 0 (-13, 14) | 0 (-14, 13) | -7 (-22, 14) | -9 (-23, 13) | -5 (-20, 11) | -8 (-22, 14) | 0 (-14, 13) | -6 (-21, 15) | -4 (-23, 17) | M | -14 (-30, 3) | -5 (-21, 10) | 0 (-14, 13) | 0 (-13, 14) | 10 (-7, 26) | -7 (-22, 14) |
| N | 14 (-7, 34) | 14 (-6, 35) | 14 (-6, 35) | 13 (-7, 34) | 7 (-14, 33) | 5 (-16, 31) | 9 (-12, 31) | 6 (-15, 32) | 13 (-7, 34) | 7 (-14, 33) | 10 (-14, 36) | 14 (-3, 30) | N | 9 (-12, 29) | 14 (-7, 34) | 14 (-6, 34) | 23 (1, 46) | 6 (-15, 32) |
| O | 5 (-10, 21) | 6 (-10, 21) | 5 (-10, 21) | 5 (-10, 20) | -2 (-18, 21) | -3 (-19, 19) | 1 (-16, 18) | -2 (-19, 20) | 5 (-10, 20) | -1 (-17, 21) | 1 (-18, 23) | 5 (-10, 21) | -9 (-29, 12) | O | 5 (-10, 20) | 5 (-10, 21) | 15 (-3, 33) | -2 (-18, 20) |
| P | 0 (-1, 1) | 1 (0, 1) | 0 (0, 1) | 0 (-1, 1) | -8 (-11, 12) | -9 (-13, 11) | -5 (-12, 3) | -8 (-12, 12) | 0 (-1, 0) | -7 (-11, 13) | -4 (-16, 12) | 0 (-13, 14) | -14 (-34, 7) | -5 (-20, 10) | P | 0 (0, 1) | 10 (0, 20) | -8 (-12, 12) |
| placebo | 0 (0, 0) | 0 (0, 1) | 0 (-1, 1) | -1 (-1, 0) | -8 (-11, 12) | -9 (-13, 11) | -5 (-12, 3) | -8 (-12, 12) | -1 (-1, 0) | -7 (-11, 13) | -4 (-17, 12) | 0 (-14, 13) | -14 (-34, 6) | -5 (-21, 10) | 0 (-1, 0) | placebo | 9 (-1, 19) | -8 (-12, 12) |
| Q | -10 (-20, 1) | -9 (-19, 1) | -9 (-19, 1) | -10 (-20, 0) | -17 (-28, 4) | -18 (-29, 2) | -14 (-27, -1) | -17 (-28, 3) | -10 (-20, 0) | -16 (-27, 4) | -14 (-30, 5) | -10 (-26, 7) | -23 (-46, -1) | -15 (-33, 3) | -10 (-20, 0) | -9 (-19, 1) | Q | -17 (-28, 3) |
| R | 8 (-12, 12) | 8 (-11, 12) | 8 (-12, 12) | 8 (-12, 11) | 0 (0, 0) | -1 (-1, -1) | 3 (-12, 10) | 0 (0, 0) | 8 (-12, 11) | 1 (1, 1) | 3 (-10, 15) | 7 (-14, 22) | -6 (-32, 15) | 2 (-20, 18) | 8 (-12, 12) | 8 (-12, 12) | 17 (-3, 28) | R |

**Table S6.** League table for HDL-C

|  | A | B | C | D | E | G | H | I | J | K | M | N | O | P | placebo | Q |
| --- | --- | --- | --- | --- | --- | --- | --- | --- | --- | --- | --- | --- | --- | --- | --- | --- |
| A | A | 0 (-2, 3) | -1 (-4, 3) | 0 (-2, 3) | 0 (-3, 2) | 1 (-2, 5) | 2 (-3, 7) | -1 (-5, 4) | 0 (-2, 3) | 1 (-5, 6) | 2 (-2, 7) | 8 (0, 17) | 0 (-7, 7) | -1 (-5, 4) | 0 (-2, 2) | -1 (-7, 6) |
| B | 0 (-3, 2) | B | -1 (-3, 2) | 0 (-2, 3) | 0 (-3, 2) | 1 (-2, 5) | 2 (-3, 7) | -1 (-5, 4) | 0 (-3, 4) | 0 (-5, 6) | 2 (-3, 7) | 8 (0, 17) | 0 (-7, 7) | -1 (-5, 3) | 0 (-2, 2) | -1 (-7, 6) |
| C | 1 (-3, 4) | 1 (-2, 3) | C | 1 (-1, 4) | 0 (-2, 3) | 2 (-2, 6) | 3 (-3, 9) | 0 (-4, 4) | 1 (-3, 5) | 1 (-5, 7) | 3 (-2, 8) | 9 (0, 18) | 1 (-6, 8) | 0 (-4, 4) | 1 (-2, 3) | 0 (-6, 6) |
| D | 0 (-3, 2) | 0 (-3, 2) | -1 (-4, 1) | D | -1 (-3, 1) | 1 (-3, 4) | 1 (-4, 7) | -1 (-5, 3) | 0 (-3, 3) | 0 (-6, 6) | 2 (-3, 6) | 8 (-1, 17) | 0 (-7, 6) | -1 (-6, 3) | 0 (-2, 1) | -1 (-8, 5) |
| E | 0 (-2, 3) | 0 (-2, 3) | 0 (-3, 2) | 1 (-1, 3) | E | 2 (-2, 5) | 2 (-3, 8) | 0 (-5, 4) | 1 (-3, 4) | 1 (-5, 6) | 2 (-2, 7) | 9 (0, 17) | 0 (-7, 7) | -1 (-5, 4) | 0 (-1, 2) | 0 (-7, 6) |
| G | -1 (-5, 2) | -1 (-5, 2) | -2 (-6, 2) | -1 (-4, 3) | -2 (-5, 2) | G | 1 (-5, 6) | -2 (-7, 3) | -1 (-5, 3) | -1 (-7, 5) | 1 (-5, 6) | 7 (-2, 16) | -1 (-9, 6) | -2 (-7, 3) | -1 (-4, 2) | -2 (-9, 5) |
| H | -2 (-7, 3) | -2 (-7, 3) | -3 (-9, 3) | -1 (-7, 4) | -2 (-8, 3) | -1 (-6, 5) | H | -2 (-9, 4) | -2 (-7, 4) | -1 (-9, 6) | 0 (-7, 7) | 6 (-4, 16) | -2 (-10, 6) | -3 (-10, 4) | -2 (-7, 3) | -3 (-11, 6) |
| I | 1 (-4, 5) | 1 (-4, 5) | 0 (-4, 4) | 1 (-3, 5) | 0 (-4, 5) | 2 (-3, 7) | 2 (-4, 9) | I | 1 (-4, 6) | 1 (-6, 8) | 3 (-3, 9) | 9 (-1, 18) | 1 (-7, 8) | 0 (-5, 4) | 1 (-4, 5) | 0 (-5, 5) |
| J | 0 (-3, 2) | 0 (-4, 3) | -1 (-5, 3) | 0 (-3, 3) | -1 (-4, 3) | 1 (-3, 5) | 2 (-4, 7) | -1 (-6, 4) | J | 0 (-5, 5) | 2 (-3, 7) | 8 (-1, 17) | 0 (-8, 7) | -1 (-6, 3) | 0 (-3, 3) | -1 (-8, 6) |
| K | -1 (-6, 5) | 0 (-6, 5) | -1 (-7, 5) | 0 (-6, 6) | -1 (-6, 5) | 1 (-5, 7) | 1 (-6, 9) | -1 (-8, 6) | 0 (-5, 5) | K | 2 (-5, 8) | 8 (-2, 18) | 0 (-9, 8) | -1 (-8, 5) | 0 (-6, 5) | -1 (-9, 7) |
| M | -2 (-7, 2) | -2 (-7, 3) | -3 (-8, 2) | -2 (-6, 3) | -2 (-7, 2) | -1 (-6, 5) | 0 (-7, 7) | -3 (-9, 3) | -2 (-7, 3) | -2 (-8, 5) | M | 6 (-2, 14) | -2 (-9, 5) | -3 (-9, 3) | -2 (-6, 2) | -3 (-10, 5) |
| N | -8 (-17, 0) | -8 (-17, 0) | -9 (-18, 0) | -8 (-17, 1) | -9 (-17, 0) | -7 (-16, 2) | -6 (-16, 4) | -9 (-18, 1) | -8 (-17, 1) | -8 (-18, 2) | -6 (-14, 2) | N | -8 (-16, -1) | -9 (-19, 0) | -8 (-17, 0) | -9 (-19, 2) |
| O | 0 (-7, 7) | 0 (-7, 7) | -1 (-8, 6) | 0 (-6, 7) | 0 (-7, 7) | 1 (-6, 9) | 2 (-6, 10) | -1 (-8, 7) | 0 (-7, 8) | 0 (-8, 9) | 2 (-5, 9) | 8 (1, 16) | O | -1 (-9, 7) | 0 (-7, 7) | -1 (-10, 9) |
| P | 1 (-4, 5) | 1 (-3, 5) | 0 (-4, 4) | 1 (-3, 6) | 1 (-4, 5) | 2 (-3, 7) | 3 (-4, 10) | 0 (-4, 5) | 1 (-3, 6) | 1 (-5, 8) | 3 (-3, 9) | 9 (0, 19) | 1 (-7, 9) | P | 1 (-3, 5) | 0 (-6, 7) |
| placebo | 0 (-2, 2) | 0 (-2, 2) | -1 (-3, 2) | 0 (-1, 2) | 0 (-2, 1) | 1 (-2, 4) | 2 (-3, 7) | -1 (-5, 4) | 0 (-3, 3) | 0 (-5, 6) | 2 (-2, 6) | 8 (0, 17) | 0 (-7, 7) | -1 (-5, 3) | placebo | -1 (-7, 6) |
| Q | 1 (-6, 7) | 1 (-6, 7) | 0 (-6, 6) | 1 (-5, 8) | 0 (-6, 7) | 2 (-5, 9) | 3 (-6, 11) | 0 (-5, 5) | 1 (-6, 8) | 1 (-7, 9) | 3 (-5, 10) | 9 (-2, 19) | 1 (-9, 10) | 0 (-7, 6) | 1 (-6, 7) | Q |

**Table S7.** League table for LDL-C

|  | A | B | C | D | E | F | G | H | I | J | K | M | N | O | P | placebo | Q |
| --- | --- | --- | --- | --- | --- | --- | --- | --- | --- | --- | --- | --- | --- | --- | --- | --- | --- |
| A | A | -2 (-11, 8) | -5 (-17, 7) | -2 (-11, 7) | -4 (-14, 5) | -2 (-25, 22) | -6 (-19, 6) | -2 (-23, 19) | -7 (-26, 11) | -1 (-15, 14) | 0 (-24, 25) | -1 (-18, 17) | -10 (-40, 20) | 0 (-20, 19) | -12 (-31, 4) | 1 (-7, 8) | 5 (-23, 33) |
| B | 2 (-8, 11) | B | -4 (-14, 7) | -1 (-9, 7) | -3 (-12, 6) | 0 (-23, 24) | -5 (-16, 8) | -1 (-22, 21) | -5 (-22, 11) | 1 (-13, 15) | 2 (-23, 27) | 1 (-17, 19) | -8 (-38, 22) | 1 (-18, 21) | -11 (-27, 4) | 2 (-4, 9) | 7 (-21, 33) |
| C | 5 (-7, 17) | 4 (-7, 14) | C | 3 (-7, 12) | 1 (-10, 12) | 3 (-21, 29) | -1 (-15, 14) | 3 (-20, 26) | -2 (-19, 15) | 4 (-12, 21) | 5 (-20, 32) | 5 (-15, 24) | -5 (-36, 26) | 5 (-16, 26) | -7 (-24, 8) | 6 (-4, 16) | 10 (-17, 37) |
| D | 2 (-7, 11) | 1 (-7, 9) | -3 (-12, 7) | D | -2 (-10, 6) | 1 (-22, 25) | -4 (-16, 9) | 0 (-21, 22) | -4 (-22, 13) | 1 (-13, 17) | 3 (-22, 28) | 2 (-16, 20) | -7 (-37, 22) | 2 (-17, 21) | -10 (-27, 6) | 3 (-2, 8) | 7 (-20, 34) |
| E | 4 (-5, 14) | 3 (-6, 12) | -1 (-12, 10) | 2 (-6, 10) | E | 3 (-21, 27) | -2 (-14, 11) | 2 (-19, 23) | -2 (-21, 15) | 3 (-12, 19) | 5 (-20, 30) | 4 (-15, 22) | -5 (-35, 25) | 4 (-15, 24) | -8 (-26, 8) | 5 (-1, 11) | 10 (-19, 37) |
| F | 2 (-22, 25) | 0 (-24, 23) | -3 (-29, 21) | -1 (-25, 22) | -3 (-27, 21) | F | -4 (-29, 20) | -1 (-31, 30) | -5 (-34, 22) | 1 (-18, 19) | 2 (-28, 31) | 1 (-28, 29) | -8 (-46, 29) | 1 (-28, 31) | -11 (-38, 15) | 2 (-21, 25) | 7 (-29, 41) |
| G | 6 (-6, 19) | 5 (-8, 16) | 1 (-14, 15) | 4 (-9, 16) | 2 (-11, 14) | 4 (-20, 29) | G | 4 (-18, 26) | -1 (-21, 19) | 5 (-11, 21) | 6 (-19, 33) | 6 (-15, 26) | -4 (-35, 28) | 6 (-16, 27) | -6 (-26, 12) | 7 (-5, 18) | 11 (-18, 40) |
| H | 2 (-19, 23) | 1 (-21, 22) | -3 (-26, 20) | 0 (-22, 21) | -2 (-23, 19) | 1 (-30, 31) | -4 (-26, 18) | H | -5 (-32, 22) | 1 (-23, 26) | 2 (-29, 34) | 2 (-25, 28) | -8 (-43, 28) | 2 (-26, 30) | -10 (-37, 15) | 3 (-18, 24) | 7 (-27, 41) |
| I | 7 (-11, 26) | 5 (-11, 22) | 2 (-15, 19) | 4 (-13, 22) | 2 (-15, 21) | 5 (-22, 34) | 1 (-19, 21) | 5 (-22, 32) | I | 6 (-14, 28) | 7 (-21, 37) | 6 (-18, 31) | -3 (-37, 31) | 7 (-18, 32) | -5 (-24, 12) | 7 (-9, 25) | 12 (-9, 33) |
| J | 1 (-14, 15) | -1 (-15, 13) | -4 (-21, 12) | -1 (-17, 13) | -3 (-19, 12) | -1 (-19, 18) | -5 (-21, 11) | -1 (-26, 23) | -6 (-28, 14) | J | 1 (-22, 24) | 0 (-22, 22) | -9 (-42, 23) | 1 (-23, 23) | -11 (-31, 7) | 2 (-13, 15) | 6 (-24, 35) |
| K | 0 (-25, 24) | -2 (-27, 23) | -5 (-32, 20) | -3 (-28, 22) | -5 (-30, 20) | -2 (-31, 28) | -6 (-33, 19) | -2 (-34, 29) | -7 (-37, 21) | -1 (-24, 22) | K | -1 (-31, 28) | -10 (-48, 28) | -1 (-31, 30) | -13 (-42, 15) | 0 (-24, 25) | 5 (-32, 41) |
| M | 1 (-17, 18) | -1 (-19, 17) | -5 (-24, 15) | -2 (-20, 16) | -4 (-22, 15) | -1 (-29, 28) | -6 (-26, 15) | -2 (-28, 25) | -6 (-31, 18) | 0 (-22, 22) | 1 (-28, 31) | M | -9 (-36, 17) | 0 (-18, 19) | -12 (-36, 11) | 1 (-16, 19) | 6 (-27, 37) |
| N | 10 (-20, 40) | 8 (-22, 38) | 5 (-26, 36) | 7 (-22, 37) | 5 (-25, 35) | 8 (-29, 46) | 4 (-28, 35) | 8 (-28, 43) | 3 (-31, 37) | 9 (-23, 42) | 10 (-28, 48) | 9 (-17, 36) | N | 9 (-18, 37) | -3 (-37, 30) | 10 (-19, 40) | 15 (-25, 54) |
| O | 0 (-19, 20) | -1 (-21, 18) | -5 (-26, 16) | -2 (-21, 17) | -4 (-24, 15) | -1 (-31, 28) | -6 (-27, 16) | -2 (-30, 26) | -7 (-32, 18) | -1 (-23, 23) | 1 (-30, 31) | 0 (-19, 18) | -9 (-37, 18) | O | -12 (-37, 11) | 1 (-17, 19) | 5 (-28, 38) |
| P | 12 (-4, 31) | 11 (-4, 27) | 7 (-8, 24) | 10 (-6, 27) | 8 (-8, 26) | 11 (-15, 38) | 6 (-12, 26) | 10 (-15, 37) | 5 (-12, 24) | 11 (-7, 31) | 13 (-15, 42) | 12 (-11, 36) | 3 (-30, 37) | 12 (-11, 37) | P | 13 (-2, 30) | 17 (-10, 46) |
| placebo | -1 (-8, 7) | -2 (-9, 4) | -6 (-16, 4) | -3 (-8, 2) | -5 (-11, 1) | -2 (-25, 21) | -7 (-18, 5) | -3 (-24, 18) | -7 (-25, 9) | -2 (-15, 13) | 0 (-25, 24) | -1 (-19, 16) | -10 (-40, 19) | -1 (-19, 17) | -13 (-30, 2) | placebo | 4 (-23, 31) |
| Q | -5 (-33, 23) | -7 (-33, 21) | -10 (-37, 17) | -7 (-34, 20) | -10 (-37, 19) | -7 (-41, 29) | -11 (-40, 18) | -7 (-41, 27) | -12 (-33, 9) | -6 (-35, 24) | -5 (-41, 32) | -6 (-37, 27) | -15 (-54, 25) | -5 (-38, 28) | -17 (-46, 10) | -4 (-31, 23) | Q |

**Table S8.** League table for FBG

|  | A | B | C | D | E | F | G | H | J | M | N | O | placebo | R |
| --- | --- | --- | --- | --- | --- | --- | --- | --- | --- | --- | --- | --- | --- | --- |
| A | A | -2 (-3, 0) | 0 (-3, 3) | 0 (0, 0) | -3 (-4, -2) | -3 (-4, -2) | -1 (-3, 2) | -4 (-6, -3) | 0 (0, 0) | 1 (-9, 10) | 2 (-8, 11) | 1 (-8, 10) | 0 (0, 0) | -3 (-4, -2) |
| B | 2 (0, 3) | B | 2 (-1, 5) | 2 (1, 4) | -1 (-3, 1) | -1 (-3, 1) | 1 (-2, 4) | -3 (-4, 0) | 2 (0, 3) | 3 (-7, 12) | 4 (-6, 13) | 3 (-6, 12) | 2 (1, 4) | -1 (-3, 1) |
| C | 0 (-3, 3) | -2 (-5, 1) | C | 0 (-3, 3) | -3 (-6, 0) | -3 (-6, 0) | -1 (-5, 3) | -5 (-8, -2) | 0 (-3, 3) | 0 (-9, 10) | 1 (-8, 11) | 1 (-8, 10) | 0 (-3, 3) | -3 (-6, 0) |
| D | 0 (0, 0) | -2 (-4, -1) | 0 (-3, 3) | D | -3 (-4, -2) | -3 (-4, -2) | -1 (-3, 2) | -4 (-6, -3) | 0 (0, 0) | 1 (-9, 10) | 1 (-8, 11) | 1 (-8, 9) | 0 (0, 0) | -3 (-4, -2) |
| E | 3 (2, 4) | 1 (-1, 3) | 3 (0, 6) | 3 (2, 4) | E | 0 (0, 0) | 2 (-1, 5) | -2 (-2, -1) | 3 (2, 4) | 3 (-6, 13) | 4 (-5, 14) | 4 (-5, 12) | 3 (2, 4) | 0 (0, 0) |
| F | 3 (2, 4) | 1 (-1, 3) | 3 (0, 6) | 3 (2, 4) | 0 (0, 0) | F | 2 (-1, 5) | -1 (-2, -1) | 3 (2, 4) | 4 (-6, 13) | 4 (-5, 14) | 4 (-5, 12) | 3 (2, 4) | 0 (0, 0) |
| G | 1 (-2, 3) | -1 (-4, 2) | 1 (-3, 5) | 1 (-2, 3) | -2 (-5, 1) | -2 (-5, 1) | G | -4 (-6, -1) | 1 (-2, 3) | 1 (-8, 11) | 2 (-8, 12) | 2 (-7, 11) | 1 (-1, 4) | -2 (-5, 1) |
| H | 4 (3, 6) | 3 (0, 4) | 5 (2, 8) | 4 (3, 6) | 2 (1, 2) | 1 (1, 2) | 4 (1, 6) | H | 4 (3, 6) | 5 (-4, 14) | 6 (-4, 16) | 5 (-4, 14) | 5 (3, 6) | 1 (1, 2) |
| J | 0 (0, 0) | -2 (-3, 0) | 0 (-3, 3) | 0 (0, 0) | -3 (-4, -2) | -3 (-4, -2) | -1 (-3, 2) | -4 (-6, -3) | J | 1 (-9, 10) | 2 (-8, 11) | 1 (-8, 10) | 0 (0, 0) | -3 (-4, -2) |
| M | -1 (-10, 9) | -3 (-12, 7) | 0 (-10, 9) | -1 (-10, 9) | -3 (-13, 6) | -4 (-13, 6) | -1 (-11, 8) | -5 (-14, 4) | -1 (-10, 9) | M | 1 (-4, 6) | 0 (-5, 5) | 0 (-10, 9) | -4 (-13, 6) |
| N | -2 (-11, 8) | -4 (-13, 6) | -1 (-11, 8) | -1 (-11, 8) | -4 (-14, 5) | -4 (-14, 5) | -2 (-12, 8) | -6 (-16, 4) | -2 (-11, 8) | -1 (-6, 4) | N | -1 (-6, 4) | -1 (-11, 8) | -5 (-14, 5) |
| O | -1 (-10, 8) | -3 (-12, 6) | -1 (-10, 8) | -1 (-9, 8) | -4 (-12, 5) | -4 (-12, 5) | -2 (-11, 7) | -5 (-14, 4) | -1 (-10, 8) | 0 (-5, 5) | 1 (-4, 6) | O | -1 (-9, 8) | -4 (-12, 5) |
| placebo | 0 (0, 0) | -2 (-4, -1) | 0 (-3, 3) | 0 (0, 0) | -3 (-4, -2) | -3 (-4, -2) | -1 (-4, 1) | -5 (-6, -3) | 0 (0, 0) | 0 (-9, 10) | 1 (-8, 11) | 1 (-8, 9) | placebo | -3 (-4, -2) |
| R | 3 (2, 4) | 1 (-1, 3) | 3 (0, 6) | 3 (2, 4) | 0 (0, 0) | 0 (0, 0) | 2 (-1, 5) | -1 (-2, -1) | 3 (2, 4) | 4 (-6, 13) | 5 (-5, 14) | 4 (-5, 12) | 3 (2, 4) | R |

**Table S9.** League table for HbA1c

|  | A | B | C | D | E | G | J | M | O | placebo |
| --- | --- | --- | --- | --- | --- | --- | --- | --- | --- | --- |
| A | A | 0 (0, 0) | 0 (0, 1) | 0 (0, 0) | 0 (0, 1) | 0 (0, 0) | 0 (0, 0) | -1 (-1, 0) | 0 (-1, 1) | 0 (0, 0) |
| B | 0 (0, 0) | B | 0 (0, 1) | 0 (0, 0) | 0 (0, 1) | 0 (0, 0) | 0 (0, 0) | -1 (-1, 0) | 0 (-1, 1) | 0 (0, 0) |
| C | 0 (-1, 0) | 0 (-1, 0) | C | 0 (0, 0) | 0 (0, 1) | 0 (-1, 0) | 0 (0, 0) | -1 (-2, 0) | 0 (-1, 0) | 0 (0, 0) |
| D | 0 (0, 0) | 0 (0, 0) | 0 (0, 0) | D | 0 (0, 1) | 0 (0, 0) | 0 (0, 0) | -1 (-1, 0) | 0 (-1, 1) | 0 (0, 0) |
| E | 0 (-1, 0) | 0 (-1, 0) | 0 (-1, 0) | 0 (-1, 0) | E | 0 (-1, 0) | 0 (-1, 0) | -1 (-2, -1) | -1 (-1, 0) | 0 (-1, 0) |
| G | 0 (0, 0) | 0 (0, 0) | 0 (0, 1) | 0 (0, 0) | 0 (0, 1) | G | 0 (0, 0) | -1 (-1, 0) | 0 (-1, 1) | 0 (0, 0) |
| J | 0 (0, 0) | 0 (0, 0) | 0 (0, 0) | 0 (0, 0) | 0 (0, 1) | 0 (0, 0) | J | -1 (-1, 0) | 0 (-1, 1) | 0 (0, 0) |
| M | 1 (0, 1) | 1 (0, 1) | 1 (0, 2) | 1 (0, 1) | 1 (1, 2) | 1 (0, 1) | 1 (0, 1) | M | 1 (0, 2) | 1 (0, 1) |
| O | 0 (-1, 1) | 0 (-1, 1) | 0 (0, 1) | 0 (-1, 1) | 1 (0, 1) | 0 (-1, 1) | 0 (-1, 1) | -1 (-2, 0) | O | 0 (-1, 1) |
| placebo | 0 (0, 0) | 0 (0, 0) | 0 (0, 0) | 0 (0, 0) | 0 (0, 1) | 0 (0, 0) | 0 (0, 0) | -1 (-1, 0) | 0 (-1, 1) | placebo |

**Table S10.** League table for Insulin

|  | A | B | C | D | E | J | M | N | O | placebo |
| --- | --- | --- | --- | --- | --- | --- | --- | --- | --- | --- |
| A | A | 0 (0, 0) | 0 (-2, 2) | 1 (0, 2) | -1 (-1, 0) | 0 (-1, 1) | -4 (-5, -2) | -4 (-9, 1) | -4 (-8, 1) | 0 (0, 1) |
| B | 0 (0, 0) | B | 0 (-2, 2) | 1 (0, 2) | -1 (-2, 0) | 0 (-1, 1) | -4 (-5, -2) | -4 (-9, 1) | -4 (-8, 1) | 0 (0, 1) |
| C | 0 (-2, 2) | 0 (-2, 2) | C | 1 (-1, 3) | -1 (-3, 1) | 0 (-2, 2) | -4 (-6, -1) | -4 (-9, 2) | -4 (-8, 1) | 0 (-2, 2) |
| D | -1 (-2, 0) | -1 (-2, 0) | -1 (-3, 1) | D | -2 (-3, -1) | -1 (-3, 0) | -5 (-7, -3) | -5 (-10, 0) | -5 (-9, 0) | -1 (-2, 0) |
| E | 1 (0, 1) | 1 (0, 2) | 1 (-1, 3) | 2 (1, 3) | E | 1 (-1, 2) | -3 (-5, -1) | -3 (-8, 2) | -3 (-7, 2) | 1 (1, 2) |
| J | 0 (-1, 1) | 0 (-1, 1) | 0 (-2, 2) | 1 (0, 3) | -1 (-2, 1) | J | -3 (-5, -1) | -4 (-9, 1) | -3 (-8, 1) | 1 (0, 2) |
| M | 4 (2, 5) | 4 (2, 5) | 4 (1, 6) | 5 (3, 7) | 3 (1, 5) | 3 (1, 5) | M | 0 (-5, 4) | 0 (-4, 4) | 4 (2, 6) |
| N | 4 (-1, 9) | 4 (-1, 9) | 4 (-2, 9) | 5 (0, 10) | 3 (-2, 8) | 4 (-1, 9) | 0 (-4, 5) | N | 0 (-4, 5) | 4 (-1, 10) |
| O | 4 (-1, 8) | 4 (-1, 8) | 4 (-1, 8) | 5 (0, 9) | 3 (-2, 7) | 3 (-1, 8) | 0 (-4, 4) | 0 (-5, 4) | O | 4 (0, 8) |
| placebo | 0 (-1, 0) | 0 (-1, 0) | 0 (-2, 2) | 1 (0, 2) | -1 (-2, -1) | -1 (-2, 0) | -4 (-6, -2) | -4 (-10, 1) | -4 (-8, 0) | placebo |

**Table S11.** League table for HOMA-IR

|  | A | B | C | D | E | I | J | M | O | P | placebo | Q |
| --- | --- | --- | --- | --- | --- | --- | --- | --- | --- | --- | --- | --- |
| A | A | 0 (0, 0) | 0 (-1, 0) | 0 (0, 0) | 0 (0, 0) | 0 (-2, 2) | 0 (0, 0) | -2 (-3, -1) | 0 (-2, 3) | 1 (-3, 5) | 0 (0, 0) | -2 (-4, 0) |
| B | 0 (0, 0) | B | 0 (-1, 0) | 0 (0, 0) | 0 (0, 0) | -1 (-3, 1) | 0 (-1, 0) | -2 (-3, -1) | 0 (-2, 2) | 1 (-3, 5) | 0 (0, 0) | -2 (-4, 0) |
| C | 0 (0, 1) | 0 (0, 1) | C | 0 (0, 1) | 0 (0, 1) | 0 (-2, 2) | 0 (0, 1) | -2 (-3, -1) | 0 (-2, 3) | 1 (-2, 5) | 0 (0, 1) | -2 (-4, 0) |
| D | 0 (0, 0) | 0 (0, 0) | 0 (-1, 0) | D | 0 (0, 0) | 0 (-3, 2) | 0 (0, 0) | -2 (-3, -1) | 0 (-2, 3) | 1 (-3, 5) | 0 (0, 0) | -2 (-4, 0) |
| E | 0 (0, 0) | 0 (0, 0) | 0 (-1, 0) | 0 (0, 0) | E | 0 (-2, 2) | 0 (0, 0) | -2 (-2, -1) | 0 (-2, 3) | 1 (-2, 5) | 0 (0, 1) | -2 (-4, 0) |
| I | 0 (-2, 2) | 1 (-1, 3) | 0 (-2, 2) | 0 (-2, 3) | 0 (-2, 2) | I | 0 (-2, 2) | -1 (-4, 1) | 1 (-2, 4) | 2 (-2, 5) | 1 (-1, 3) | -1 (-2, -1) |
| J | 0 (0, 0) | 0 (0, 1) | 0 (-1, 0) | 0 (0, 0) | 0 (0, 0) | 0 (-2, 2) | J | -2 (-3, -1) | 0 (-2, 3) | 1 (-3, 5) | 0 (0, 1) | -2 (-4, 0) |
| M | 2 (1, 3) | 2 (1, 3) | 2 (1, 3) | 2 (1, 3) | 2 (1, 2) | 1 (-1, 4) | 2 (1, 3) | M | 2 (0, 5) | 3 (-1, 7) | 2 (1, 3) | 0 (-2, 2) |
| O | 0 (-3, 2) | 0 (-2, 2) | 0 (-3, 2) | 0 (-3, 2) | 0 (-3, 2) | -1 (-4, 2) | 0 (-3, 2) | -2 (-5, 0) | O | 1 (-4, 5) | 0 (-2, 2) | -2 (-5, 1) |
| P | -1 (-5, 3) | -1 (-5, 3) | -1 (-5, 2) | -1 (-5, 3) | -1 (-5, 2) | -2 (-5, 2) | -1 (-5, 3) | -3 (-7, 1) | -1 (-5, 4) | P | -1 (-5, 3) | -3 (-7, 1) |
| placebo | 0 (0, 0) | 0 (0, 0) | 0 (-1, 0) | 0 (0, 0) | 0 (-1, 0) | -1 (-3, 1) | 0 (-1, 0) | -2 (-3, -1) | 0 (-2, 2) | 1 (-3, 5) | placebo | -2 (-4, 0) |
| Q | 2 (0, 4) | 2 (0, 4) | 2 (0, 4) | 2 (0, 4) | 2 (0, 4) | 1 (1, 2) | 2 (0, 4) | 0 (-2, 2) | 2 (-1, 5) | 3 (-1, 7) | 2 (0, 4) | Q |

**Table S12.** League table for ALT

|  | A | B | C | D | E | F | G | H | I | J | K | M | N | O | P | placebo | Q | R |
| --- | --- | --- | --- | --- | --- | --- | --- | --- | --- | --- | --- | --- | --- | --- | --- | --- | --- | --- |
| A | A | 1 (-5, 8) | -3 (-12, 6) | 1 (-6, 7) | -3 (-9, 3) | -13 (-25, -2) | -2 (-11, 7) | 9 (-2, 19) | -15 (-34, 4) | 3 (-6, 12) | 32 (12, 52) | 13 (-10, 36) | 7 (-15, 31) | 3 (-12, 18) | -6 (-20, 8) | 5 (0, 10) | -29 (-53, -5) | -16 (-30, -3) |
| B | -1 (-8, 5) | B | -5 (-13, 3) | -1 (-7, 5) | -4 (-11, 2) | -15 (-27, -3) | -4 (-13, 5) | 7 (-4, 18) | -17 (-35, 2) | 2 (-8, 11) | 31 (10, 52) | 12 (-12, 35) | 6 (-17, 29) | 2 (-13, 17) | -7 (-20, 6) | 4 (-1, 9) | -31 (-54, -7) | -18 (-32, -4) |
| C | 3 (-6, 12) | 5 (-3, 13) | C | 4 (-3, 11) | 0 (-8, 8) | -10 (-24, 3) | 1 (-10, 12) | 12 (0, 24) | -12 (-31, 7) | 7 (-5, 18) | 35 (14, 57) | 16 (-7, 39) | 11 (-12, 34) | 6 (-8, 21) | -3 (-17, 11) | 8 (1, 16) | -26 (-50, -2) | -13 (-28, 2) |
| D | -1 (-7, 6) | 1 (-5, 7) | -4 (-11, 3) | D | -4 (-9, 2) | -14 (-26, -2) | -3 (-12, 6) | 8 (-2, 19) | -16 (-35, 3) | 3 (-7, 12) | 32 (11, 52) | 12 (-11, 35) | 7 (-16, 30) | 3 (-12, 17) | -6 (-20, 7) | 5 (1, 8) | -30 (-53, -6) | -17 (-31, -3) |
| E | 3 (-3, 9) | 4 (-2, 11) | 0 (-8, 8) | 4 (-2, 9) | E | -10 (-22, 1) | 1 (-8, 10) | 12 (2, 22) | -12 (-31, 7) | 6 (-3, 16) | 35 (15, 56) | 16 (-7, 39) | 10 (-12, 33) | 6 (-8, 21) | -3 (-17, 11) | 8 (4, 12) | -26 (-50, -2) | -13 (-26, 0) |
| F | 13 (2, 25) | 15 (3, 27) | 10 (-3, 24) | 14 (2, 26) | 10 (-1, 22) | F | 11 (-3, 25) | 22 (10, 35) | -2 (-23, 20) | 17 (5, 28) | 45 (23, 68) | 26 (1, 52) | 21 (-4, 46) | 16 (-1, 35) | 7 (-9, 24) | 18 (7, 30) | -16 (-42, 10) | -3 (-17, 11) |
| G | 2 (-7, 11) | 4 (-5, 13) | -1 (-12, 10) | 3 (-6, 12) | -1 (-10, 8) | -11 (-25, 3) | G | 11 (-1, 23) | -13 (-33, 7) | 6 (-6, 17) | 35 (13, 56) | 15 (-9, 40) | 10 (-14, 34) | 6 (-11, 22) | -3 (-19, 12) | 8 (-1, 16) | -27 (-52, -2) | -14 (-29, 1) |
| H | -9 (-19, 2) | -7 (-18, 4) | -12 (-24, 0) | -8 (-19, 2) | -12 (-22, -2) | -22 (-35, -10) | -11 (-23, 1) | H | -24 (-45, -3) | -6 (-18, 7) | 23 (1, 46) | 4 (-21, 29) | -1 (-26, 23) | -6 (-23, 12) | -15 (-31, 2) | -4 (-13, 6) | -38 (-63, -12) | -25 (-39, -12) |
| I | 15 (-4, 34) | 17 (-2, 35) | 12 (-7, 31) | 16 (-3, 35) | 12 (-7, 31) | 2 (-20, 23) | 13 (-7, 33) | 24 (3, 45) | I | 18 (-1, 38) | 47 (20, 75) | 28 (-1, 57) | 23 (-6, 52) | 18 (-4, 41) | 9 (-10, 29) | 20 (2, 39) | -14 (-29, 0) | -1 (-24, 21) |
| J | -3 (-12, 6) | -2 (-11, 8) | -7 (-18, 5) | -3 (-12, 7) | -6 (-16, 3) | -17 (-28, -5) | -6 (-17, 6) | 6 (-7, 18) | -18 (-38, 1) | J | 29 (8, 49) | 10 (-15, 34) | 4 (-20, 28) | 0 (-17, 17) | -9 (-23, 5) | 2 (-7, 11) | -33 (-57, -8) | -20 (-35, -5) |
| K | -32 (-52, -12) | -31 (-52, -10) | -35 (-57, -14) | -32 (-52, -11) | -35 (-56, -15) | -45 (-68, -23) | -35 (-56, -13) | -23 (-46, -1) | -47 (-75, -20) | -29 (-49, -8) | K | -19 (-50, 11) | -25 (-55, 6) | -29 (-54, -4) | -38 (-62, -14) | -27 (-48, -7) | -61 (-92, -31) | -48 (-73, -25) |
| M | -13 (-36, 10) | -12 (-35, 12) | -16 (-39, 7) | -12 (-35, 11) | -16 (-39, 7) | -26 (-52, -1) | -15 (-40, 9) | -4 (-29, 21) | -28 (-57, 1) | -10 (-34, 15) | 19 (-11, 50) | M | -5 (-23, 12) | -10 (-28, 8) | -19 (-45, 7) | -8 (-30, 15) | -42 (-75, -10) | -29 (-56, -3) |
| N | -7 (-31, 15) | -6 (-29, 17) | -11 (-34, 12) | -7 (-30, 16) | -10 (-33, 12) | -21 (-46, 4) | -10 (-34, 14) | 1 (-23, 26) | -23 (-52, 6) | -4 (-28, 20) | 25 (-6, 55) | 5 (-12, 23) | N | -4 (-22, 13) | -13 (-39, 13) | -2 (-25, 20) | -37 (-69, -4) | -24 (-50, 2) |
| O | -3 (-18, 12) | -2 (-17, 13) | -6 (-21, 8) | -3 (-17, 12) | -6 (-21, 8) | -16 (-35, 1) | -6 (-22, 11) | 6 (-12, 23) | -18 (-41, 4) | 0 (-17, 17) | 29 (4, 54) | 10 (-8, 28) | 4 (-13, 22) | O | -9 (-28, 10) | 2 (-12, 16) | -33 (-60, -5) | -19 (-39, 0) |
| P | 6 (-8, 20) | 7 (-6, 20) | 3 (-11, 17) | 6 (-7, 20) | 3 (-11, 17) | -7 (-24, 9) | 3 (-12, 19) | 15 (-2, 31) | -9 (-29, 10) | 9 (-5, 23) | 38 (14, 62) | 19 (-7, 45) | 13 (-13, 39) | 9 (-10, 28) | P | 11 (-2, 24) | -24 (-47, 1) | -10 (-29, 8) |
| placebo | -5 (-10, 0) | -4 (-9, 1) | -8 (-16, -1) | -5 (-8, -1) | -8 (-12, -4) | -18 (-30, -7) | -8 (-16, 1) | 4 (-6, 13) | -20 (-39, -2) | -2 (-11, 7) | 27 (7, 48) | 8 (-15, 30) | 2 (-20, 25) | -2 (-16, 12) | -11 (-24, 2) | placebo | -35 (-58, -11) | -21 (-35, -8) |
| Q | 29 (5, 53) | 31 (7, 54) | 26 (2, 50) | 30 (6, 53) | 26 (2, 50) | 16 (-10, 42) | 27 (2, 52) | 38 (12, 63) | 14 (0, 29) | 33 (8, 57) | 61 (31, 92) | 42 (10, 75) | 37 (4, 69) | 33 (5, 60) | 24 (-1, 47) | 35 (11, 58) | Q | 13 (-14, 40) |
| R | 16 (3, 30) | 18 (4, 32) | 13 (-2, 28) | 17 (3, 31) | 13 (0, 26) | 3 (-11, 17) | 14 (-1, 29) | 25 (12, 39) | 1 (-21, 24) | 20 (5, 35) | 48 (25, 73) | 29 (3, 56) | 24 (-2, 50) | 19 (0, 39) | 10 (-8, 29) | 21 (8, 35) | -13 (-40, 14) | R |

**Table S13.** League table for AST

|  | A | B | C | D | E | F | G | H | I | J | K | M | N | O | P | placebo | Q | R |
| --- | --- | --- | --- | --- | --- | --- | --- | --- | --- | --- | --- | --- | --- | --- | --- | --- | --- | --- |
| A | A | 1 (-1, 3) | -5 (-7, -2) | -2 (-3, 0) | 2 (0, 3) | -15 (-19, -12) | -2 (-5, 2) | 1 (-2, 5) | -6 (-16, 3) | 0 (-3, 3) | 23 (12, 33) | 9 (-5, 23) | 10 (1, 18) | 0 (-5, 5) | 0 (-5, 6) | 2 (1, 4) | -27 (-37, -17) | -5 (-8, -1) |
| B | -1 (-3, 1) | B | -5 (-8, -3) | -2 (-4, -1) | 1 (-1, 2) | -16 (-20, -13) | -3 (-6, 1) | 1 (-3, 4) | -7 (-16, 2) | -1 (-4, 3) | 22 (11, 33) | 8 (-6, 22) | 9 (0, 17) | -1 (-6, 4) | -1 (-6, 5) | 1 (0, 3) | -28 (-38, -18) | -5 (-9, -2) |
| C | 5 (2, 7) | 5 (3, 8) | C | 3 (1, 5) | 6 (4, 9) | -11 (-15, -7) | 3 (-1, 7) | 6 (2, 10) | -1 (-11, 8) | 5 (1, 9) | 28 (17, 38) | 14 (0, 28) | 14 (6, 23) | 4 (-1, 10) | 5 (-1, 11) | 7 (5, 9) | -22 (-32, -12) | 0 (-4, 4) |
| D | 2 (0, 3) | 2 (1, 4) | -3 (-5, -1) | D | 3 (2, 4) | -14 (-17, -10) | 0 (-4, 3) | 3 (0, 6) | -5 (-14, 5) | 2 (-2, 5) | 25 (14, 35) | 11 (-3, 25) | 11 (3, 20) | 1 (-3, 6) | 2 (-4, 8) | 4 (3, 5) | -25 (-35, -15) | -3 (-6, 0) |
| E | -2 (-3, 0) | -1 (-2, 1) | -6 (-9, -4) | -3 (-4, -2) | E | -17 (-20, -14) | -3 (-7, 0) | 0 (-4, 3) | -8 (-17, 2) | -2 (-5, 2) | 21 (11, 32) | 7 (-7, 22) | 8 (0, 17) | -2 (-7, 3) | -1 (-7, 4) | 1 (0, 1) | -28 (-39, -18) | -6 (-9, -3) |
| F | 15 (12, 19) | 16 (13, 20) | 11 (7, 15) | 14 (10, 17) | 17 (14, 20) | F | 14 (9, 18) | 17 (12, 21) | 9 (-1, 19) | 15 (13, 18) | 38 (27, 49) | 24 (10, 39) | 25 (16, 34) | 15 (9, 21) | 16 (9, 22) | 18 (14, 21) | -11 (-22, -1) | 11 (7, 14) |
| G | 2 (-2, 5) | 3 (-1, 6) | -3 (-7, 1) | 0 (-3, 4) | 3 (0, 7) | -14 (-18, -9) | G | 3 (-1, 8) | -4 (-14, 6) | 2 (-3, 6) | 25 (14, 36) | 11 (-4, 25) | 11 (2, 21) | 2 (-4, 8) | 2 (-5, 9) | 4 (1, 8) | -25 (-36, -14) | -3 (-8, 2) |
| H | -1 (-5, 2) | -1 (-4, 3) | -6 (-10, -2) | -3 (-6, 0) | 0 (-3, 4) | -17 (-21, -12) | -3 (-8, 1) | H | -7 (-17, 3) | -1 (-6, 3) | 22 (11, 33) | 8 (-7, 22) | 8 (-1, 17) | -2 (-7, 4) | -1 (-8, 6) | 1 (-2, 4) | -28 (-39, -18) | -6 (-10, -2) |
| I | 6 (-3, 16) | 7 (-2, 16) | 1 (-8, 11) | 5 (-5, 14) | 8 (-2, 17) | -9 (-19, 1) | 4 (-6, 14) | 7 (-3, 17) | I | 6 (-4, 16) | 29 (15, 43) | 15 (-2, 32) | 16 (3, 28) | 6 (-5, 16) | 6 (-3, 16) | 8 (-1, 18) | -21 (-24, -17) | 2 (-8, 11) |
| J | 0 (-3, 3) | 1 (-3, 4) | -5 (-9, -1) | -2 (-5, 2) | 2 (-2, 5) | -15 (-18, -13) | -2 (-6, 3) | 1 (-3, 6) | -6 (-16, 4) | J | 23 (12, 34) | 9 (-5, 24) | 10 (1, 19) | 0 (-6, 6) | 0 (-6, 7) | 2 (-1, 6) | -27 (-37, -16) | -4 (-9, -1) |
| K | -23 (-33, -12) | -22 (-33, -11) | -28 (-38, -17) | -25 (-35, -14) | -21 (-32, -11) | -38 (-49, -27) | -25 (-36, -14) | -22 (-33, -11) | -29 (-43, -15) | -23 (-34, -12) | K | -14 (-31, 4) | -13 (-27, 0) | -23 (-35, -12) | -23 (-34, -11) | -21 (-31, -10) | -50 (-64, -35) | -27 (-38, -17) |
| M | -9 (-23, 5) | -8 (-22, 6) | -14 (-28, 0) | -11 (-25, 3) | -7 (-22, 7) | -24 (-39, -10) | -11 (-25, 4) | -8 (-22, 7) | -15 (-32, 2) | -9 (-24, 5) | 14 (-4, 31) | M | 1 (-12, 13) | -9 (-23, 4) | -9 (-24, 6) | -7 (-21, 7) | -36 (-53, -19) | -14 (-28, 1) |
| N | -10 (-18, -1) | -9 (-17, 0) | -14 (-23, -6) | -11 (-20, -3) | -8 (-17, 0) | -25 (-34, -16) | -11 (-21, -2) | -8 (-17, 1) | -16 (-28, -3) | -10 (-19, -1) | 13 (0, 27) | -1 (-13, 12) | N | -10 (-17, -3) | -9 (-20, 1) | -7 (-16, 1) | -36 (-50, -23) | -14 (-23, -5) |
| O | 0 (-5, 5) | 1 (-4, 6) | -4 (-10, 1) | -1 (-6, 3) | 2 (-3, 7) | -15 (-21, -9) | -2 (-8, 4) | 2 (-4, 7) | -6 (-16, 5) | 0 (-6, 6) | 23 (12, 35) | 9 (-4, 23) | 10 (3, 17) | O | 1 (-7, 8) | 3 (-2, 7) | -27 (-38, -15) | -4 (-10, 1) |
| P | 0 (-6, 5) | 1 (-5, 6) | -5 (-11, 1) | -2 (-8, 4) | 1 (-4, 7) | -16 (-22, -9) | -2 (-9, 5) | 1 (-6, 8) | -6 (-16, 3) | 0 (-7, 6) | 23 (11, 34) | 9 (-6, 24) | 9 (-1, 20) | -1 (-8, 7) | P | 2 (-4, 8) | -27 (-37, -17) | -5 (-11, 2) |
| placebo | -2 (-4, -1) | -1 (-3, 0) | -7 (-9, -5) | -4 (-5, -3) | -1 (-1, 0) | -18 (-21, -14) | -4 (-8, -1) | -1 (-4, 2) | -8 (-18, 1) | -2 (-6, 1) | 21 (10, 31) | 7 (-7, 21) | 7 (-1, 16) | -3 (-7, 2) | -2 (-8, 4) | placebo | -29 (-39, -19) | -7 (-10, -4) |
| Q | 27 (17, 37) | 28 (18, 38) | 22 (12, 32) | 25 (15, 35) | 28 (18, 39) | 11 (1, 22) | 25 (14, 36) | 28 (18, 39) | 21 (17, 24) | 27 (16, 37) | 50 (35, 64) | 36 (19, 53) | 36 (23, 50) | 27 (15, 38) | 27 (17, 37) | 29 (19, 39) | Q | 22 (12, 33) |
| R | 5 (1, 8) | 5 (2, 9) | 0 (-4, 4) | 3 (0, 6) | 6 (3, 9) | -11 (-14, -7) | 3 (-2, 8) | 6 (2, 10) | -2 (-11, 8) | 4 (1, 9) | 27 (17, 38) | 14 (-1, 28) | 14 (5, 23) | 4 (-1, 10) | 5 (-2, 11) | 7 (4, 10) | -22 (-33, -12) | R |

**Table S14.** League table for ALP

|  | A | B | D | E | G | J | P | placebo |
| --- | --- | --- | --- | --- | --- | --- | --- | --- |
| A | A | -4 (-13, 4) | 5 (-1, 11) | 2 (-6, 9) | -5 (-14, 3) | 4 (-6, 13) | -2 (-24, 19) | 4 (1, 6) |
| B | 4 (-4, 13) | B | 9 (0, 19) | 6 (-4, 16) | -1 (-7, 6) | 8 (0, 16) | 2 (-18, 23) | 8 (1, 16) |
| D | -5 (-11, 1) | -9 (-19, 0) | D | -3 (-12, 6) | -10 (-20, 0) | -1 (-12, 10) | -7 (-29, 15) | -1 (-7, 5) |
| E | -2 (-9, 6) | -6 (-16, 4) | 3 (-6, 12) | E | -7 (-17, 3) | 2 (-10, 14) | -4 (-26, 19) | 2 (-4, 9) |
| G | 5 (-3, 14) | 1 (-6, 7) | 10 (0, 20) | 7 (-3, 17) | G | 9 (0, 17) | 3 (-18, 24) | 9 (1, 17) |
| J | -4 (-13, 6) | -8 (-16, 0) | 1 (-10, 12) | -2 (-14, 10) | -9 (-17, 0) | J | -6 (-25, 13) | 0 (-9, 10) |
| P | 2 (-19, 24) | -2 (-23, 18) | 7 (-15, 29) | 4 (-19, 26) | -3 (-24, 18) | 6 (-13, 25) | P | 6 (-15, 27) |
| placebo | -4 (-6, -1) | -8 (-16, -1) | 1 (-5, 7) | -2 (-9, 4) | -9 (-17, -1) | 0 (-10, 9) | -6 (-27, 15) | placebo |

**Table S15.** League table for GGT

|  | A | B | C | D | E | F | H | I | J | O | P | placebo | Q | R |
| --- | --- | --- | --- | --- | --- | --- | --- | --- | --- | --- | --- | --- | --- | --- |
| A | A | 2 (-7, 11) | 7 (-9, 22) | -1 (-10, 8) | -1 (-8, 5) | -29 (-39, -19) | -17 (-30, -5) | -85 (-170, -5) | 3 (-7, 13) | -7 (-23, 10) | -33 (-59, -6) | 2 (-3, 8) | -98 (-182, -19) | -10 (-22, 1) |
| B | -2 (-11, 7) | B | 5 (-11, 21) | -3 (-13, 7) | -3 (-11, 5) | -31 (-43, -19) | -19 (-33, -6) | -87 (-171, -7) | 1 (-11, 13) | -9 (-26, 9) | -35 (-62, -7) | 1 (-7, 8) | -100 (-184, -21) | -12 (-25, 1) |
| C | -7 (-22, 9) | -5 (-21, 11) | C | -7 (-21, 6) | -8 (-23, 7) | -35 (-53, -18) | -24 (-43, -6) | -92 (-177, -10) | -3 (-21, 14) | -13 (-32, 6) | -39 (-70, -9) | -4 (-19, 10) | -104 (-190, -24) | -17 (-34, 1) |
| D | 1 (-8, 10) | 3 (-7, 13) | 7 (-6, 21) | D | 0 (-8, 8) | -28 (-40, -16) | -17 (-30, -3) | -84 (-169, -4) | 4 (-9, 16) | -6 (-22, 11) | -32 (-60, -4) | 3 (-4, 11) | -97 (-181, -18) | -9 (-22, 4) |
| E | 1 (-5, 8) | 3 (-5, 11) | 8 (-7, 23) | 0 (-8, 8) | E | -28 (-37, -19) | -16 (-27, -5) | -84 (-168, -4) | 4 (-6, 14) | -5 (-21, 11) | -31 (-58, -5) | 4 (0, 7) | -97 (-181, -17) | -9 (-19, 1) |
| F | 29 (19, 39) | 31 (19, 43) | 35 (18, 53) | 28 (16, 40) | 28 (19, 37) | F | 11 (0, 22) | -56 (-140, 23) | 32 (23, 41) | 22 (4, 41) | -4 (-30, 22) | 31 (22, 41) | -69 (-153, 10) | 19 (9, 29) |
| H | 17 (5, 30) | 19 (6, 33) | 24 (6, 43) | 17 (3, 30) | 16 (5, 27) | -11 (-22, 0) | H | -68 (-152, 12) | 21 (7, 34) | 11 (-8, 30) | -15 (-43, 13) | 20 (9, 32) | -80 (-165, -1) | 7 (-4, 19) |
| I | 85 (5, 170) | 87 (7, 171) | 92 (10, 177) | 84 (4, 169) | 84 (4, 168) | 56 (-23, 140) | 68 (-12, 152) | I | 88 (9, 172) | 78 (-2, 165) | 52 (-23, 133) | 88 (8, 172) | -13 (-23, -2) | 75 (-5, 160) |
| J | -3 (-13, 7) | -1 (-13, 11) | 3 (-14, 21) | -4 (-16, 9) | -4 (-14, 6) | -32 (-41, -23) | -21 (-34, -7) | -88 (-172, -9) | J | -10 (-28, 9) | -36 (-61, -11) | -1 (-11, 10) | -101 (-185, -23) | -13 (-25, -1) |
| O | 7 (-10, 23) | 9 (-9, 26) | 13 (-6, 32) | 6 (-11, 22) | 5 (-11, 21) | -22 (-41, -4) | -11 (-30, 8) | -78 (-165, 2) | 10 (-9, 28) | O | -26 (-57, 5) | 9 (-7, 25) | -91 (-177, -11) | -3 (-22, 15) |
| P | 33 (6, 59) | 35 (7, 62) | 39 (9, 70) | 32 (4, 60) | 31 (5, 58) | 4 (-22, 30) | 15 (-13, 43) | -52 (-133, 23) | 36 (11, 61) | 26 (-5, 57) | P | 35 (9, 62) | -65 (-145, 10) | 23 (-5, 50) |
| placebo | -2 (-8, 3) | -1 (-8, 7) | 4 (-10, 19) | -3 (-11, 4) | -4 (-7, 0) | -31 (-41, -22) | -20 (-32, -9) | -88 (-172, -8) | 1 (-10, 11) | -9 (-25, 7) | -35 (-62, -9) | placebo | -100 (-184, -21) | -13 (-23, -2) |
| Q | 98 (19, 182) | 100 (21, 184) | 104 (24, 190) | 97 (18, 181) | 97 (17, 181) | 69 (-10, 153) | 80 (1, 165) | 13 (2, 23) | 101 (23, 185) | 91 (11, 177) | 65 (-10, 145) | 100 (21, 184) | Q | 88 (8, 172) |
| R | 10 (-1, 22) | 12 (-1, 25) | 17 (-1, 34) | 9 (-4, 22) | 9 (-1, 19) | -19 (-29, -9) | -7 (-19, 4) | -75 (-160, 5) | 13 (1, 25) | 3 (-15, 22) | -23 (-50, 5) | 13 (2, 23) | -88 (-172, -8) | R |

**Table S16.** League table for IL-6

|  | A | B | C | D | E | J | K | L | M | P | placebo |
| --- | --- | --- | --- | --- | --- | --- | --- | --- | --- | --- | --- |
| A | A | -1 (-6, 4) | -7 (-18, 4) | 0 (-4, 4) | 1 (-3, 5) | 16 (7, 24) | 21 (15, 27) | 2 (-4, 8) | 0 (-4, 3) | 2 (-7, 12) | 2 (-2, 5) |
| B | 1 (-4, 6) | B | -6 (-17, 5) | 1 (-3, 6) | 2 (-2, 7) | 17 (7, 26) | 22 (14, 30) | 3 (-4, 9) | 1 (-6, 7) | 3 (-7, 14) | 3 (-1, 7) |
| C | 7 (-4, 18) | 6 (-5, 17) | C | 7 (-3, 17) | 8 (-3, 19) | 22 (9, 36) | 28 (15, 40) | 9 (-3, 20) | 7 (-5, 18) | 9 (-5, 24) | 9 (-2, 19) |
| D | 0 (-4, 4) | -1 (-6, 3) | -7 (-17, 3) | D | 1 (-2, 3) | 15 (6, 25) | 21 (14, 28) | 2 (-4, 7) | 0 (-6, 5) | 2 (-8, 13) | 2 (0, 3) |
| E | -1 (-5, 3) | -2 (-7, 2) | -8 (-19, 3) | -1 (-3, 2) | E | 14 (5, 24) | 20 (13, 27) | 0 (-5, 6) | -2 (-7, 4) | 1 (-9, 12) | 1 (-1, 3) |
| J | -16 (-24, -7) | -17 (-26, -7) | -22 (-36, -9) | -15 (-25, -6) | -14 (-24, -5) | J | 6 (-2, 13) | -14 (-24, -4) | -16 (-25, -7) | -13 (-18, -8) | -14 (-23, -5) |
| K | -21 (-27, -15) | -22 (-30, -14) | -28 (-40, -15) | -21 (-28, -14) | -20 (-27, -13) | -6 (-13, 2) | K | -19 (-28, -11) | -21 (-29, -14) | -19 (-28, -10) | -19 (-26, -12) |
| L | -2 (-8, 4) | -3 (-9, 4) | -9 (-20, 3) | -2 (-7, 4) | 0 (-6, 5) | 14 (4, 24) | 19 (11, 28) | L | -2 (-9, 5) | 1 (-10, 12) | 0 (-5, 5) |
| M | 0 (-3, 4) | -1 (-7, 6) | -7 (-18, 5) | 0 (-5, 6) | 2 (-4, 7) | 16 (7, 25) | 21 (14, 29) | 2 (-5, 9) | M | 3 (-7, 13) | 2 (-3, 7) |
| P | -2 (-12, 7) | -3 (-14, 7) | -9 (-24, 5) | -2 (-13, 8) | -1 (-12, 9) | 13 (8, 18) | 19 (10, 28) | -1 (-12, 10) | -3 (-13, 7) | P | 0 (-11, 10) |
| placebo | -2 (-5, 2) | -3 (-7, 1) | -9 (-19, 2) | -2 (-3, 0) | -1 (-3, 1) | 14 (5, 23) | 19 (12, 26) | 0 (-5, 5) | -2 (-7, 3) | 0 (-10, 11) | placebo |

**Table S17.** League table for TNF-α

|  | A | B | C | D | E | M | placebo |
| --- | --- | --- | --- | --- | --- | --- | --- |
| A | A | -5 (-18, 6) | 3 (-11, 17) | -2 (-13, 8) | 1 (-10, 11) | -3 (-10, 4) | 2 (-7, 12) |
| B | 5 (-6, 18) | B | 8 (-4, 21) | 3 (-5, 12) | 6 (-2, 14) | 2 (-11, 17) | 7 (0, 15) |
| C | -3 (-17, 11) | -8 (-21, 4) | C | -5 (-15, 4) | -2 (-13, 8) | -6 (-21, 10) | -1 (-11, 9) |
| D | 2 (-8, 13) | -3 (-12, 5) | 5 (-4, 15) | D | 3 (-2, 9) | -1 (-13, 12) | 4 (0, 9) |
| E | -1 (-11, 10) | -6 (-14, 2) | 2 (-8, 13) | -3 (-9, 2) | E | -4 (-16, 9) | 1 (-2, 5) |
| M | 3 (-4, 10) | -2 (-17, 11) | 6 (-10, 21) | 1 (-12, 13) | 4 (-9, 16) | M | 5 (-7, 17) |
| placebo | -2 (-12, 7) | -7 (-15, 0) | 1 (-9, 11) | -4 (-9, 0) | -1 (-5, 2) | -5 (-17, 7) | placebo |

**Table S18.** League table for TAC

|  | A | B | C | D | E | M | placebo |
| --- | --- | --- | --- | --- | --- | --- | --- |
| A | A | -5 (-18, 6) | 3 (-11, 17) | -2 (-13, 8) | 1 (-10, 11) | -3 (-10, 4) | 2 (-7, 12) |
| B | 5 (-6, 18) | B | 8 (-4, 21) | 3 (-5, 12) | 6 (-2, 14) | 2 (-11, 17) | 7 (0, 15) |
| C | -3 (-17, 11) | -8 (-21, 4) | C | -5 (-15, 4) | -2 (-13, 8) | -6 (-21, 10) | -1 (-11, 9) |
| D | 2 (-8, 13) | -3 (-12, 5) | 5 (-4, 15) | D | 3 (-2, 9) | -1 (-13, 12) | 4 (0, 9) |
| E | -1 (-11, 10) | -6 (-14, 2) | 2 (-8, 13) | -3 (-9, 2) | E | -4 (-16, 9) | 1 (-2, 5) |
| M | 3 (-4, 10) | -2 (-17, 11) | 6 (-10, 21) | 1 (-12, 13) | 4 (-9, 16) | M | 5 (-7, 17) |
| placebo | -2 (-12, 7) | -7 (-15, 0) | 1 (-9, 11) | -4 (-9, 0) | -1 (-5, 2) | -5 (-17, 7) | placebo |

**Table S19.** League table for SOD

|  | A | B | C | D | E | M | placebo |
| --- | --- | --- | --- | --- | --- | --- | --- |
| A | A | -5 (-18, 6) | 3 (-11, 17) | -2 (-13, 8) | 1 (-10, 11) | -3 (-10, 4) | 2 (-7, 12) |
| B | 5 (-6, 18) | B | 8 (-4, 21) | 3 (-5, 12) | 6 (-2, 14) | 2 (-11, 17) | 7 (0, 15) |
| C | -3 (-17, 11) | -8 (-21, 4) | C | -5 (-15, 4) | -2 (-13, 8) | -6 (-21, 10) | -1 (-11, 9) |
| D | 2 (-8, 13) | -3 (-12, 5) | 5 (-4, 15) | D | 3 (-2, 9) | -1 (-13, 12) | 4 (0, 9) |
| E | -1 (-11, 10) | -6 (-14, 2) | 2 (-8, 13) | -3 (-9, 2) | E | -4 (-16, 9) | 1 (-2, 5) |
| M | 3 (-4, 10) | -2 (-17, 11) | 6 (-10, 21) | 1 (-12, 13) | 4 (-9, 16) | M | 5 (-7, 17) |
| placebo | -2 (-12, 7) | -7 (-15, 0) | 1 (-9, 11) | -4 (-9, 0) | -1 (-5, 2) | -5 (-17, 7) | placebo |

**Table S20.** League table for MDA

|  | A | B | D | E | L | placebo |
| --- | --- | --- | --- | --- | --- | --- |
| A | A | -2 (-7, 3) | 1 (-5, 6) | 1 (-5, 6) | 0 (-8, 8) | 1 (-2, 4) |
| B | 2 (-3, 7) | B | 3 (-3, 9) | 3 (-3, 8) | 2 (-6, 10) | 3 (-1, 7) |
| D | -1 (-6, 5) | -3 (-9, 3) | D | 0 (-6, 6) | -1 (-9, 7) | 0 (-4, 5) |
| E | -1 (-6, 5) | -3 (-8, 3) | 0 (-6, 6) | E | -1 (-9, 8) | 0 (-4, 5) |
| L | 0 (-8, 8) | -2 (-10, 6) | 1 (-7, 9) | 1 (-8, 9) | L | 1 (-6, 8) |
| placebo | -1 (-4, 2) | -3 (-7, 1) | 0 (-5, 4) | 0 (-5, 4) | -1 (-8, 6) | placebo |
