# Supplementary material for: Efficacy of selected dietary supplements and pharmacological agents on metabolic and oxidative stress outcomes in metabolic dysfunction–associated fatty liver disease (MAFLD): a Bayesian network meta-analysis
Source: Front Pharmacol. 2026 Jan 29;16:1682688. doi: 10.3389/fphar.2025.1682688 (PMC12894030; doi:10.3389/fphar.2025.1682688)
Supplement: Supplementary file 3 [file Supplementaryfile2.docx]

**
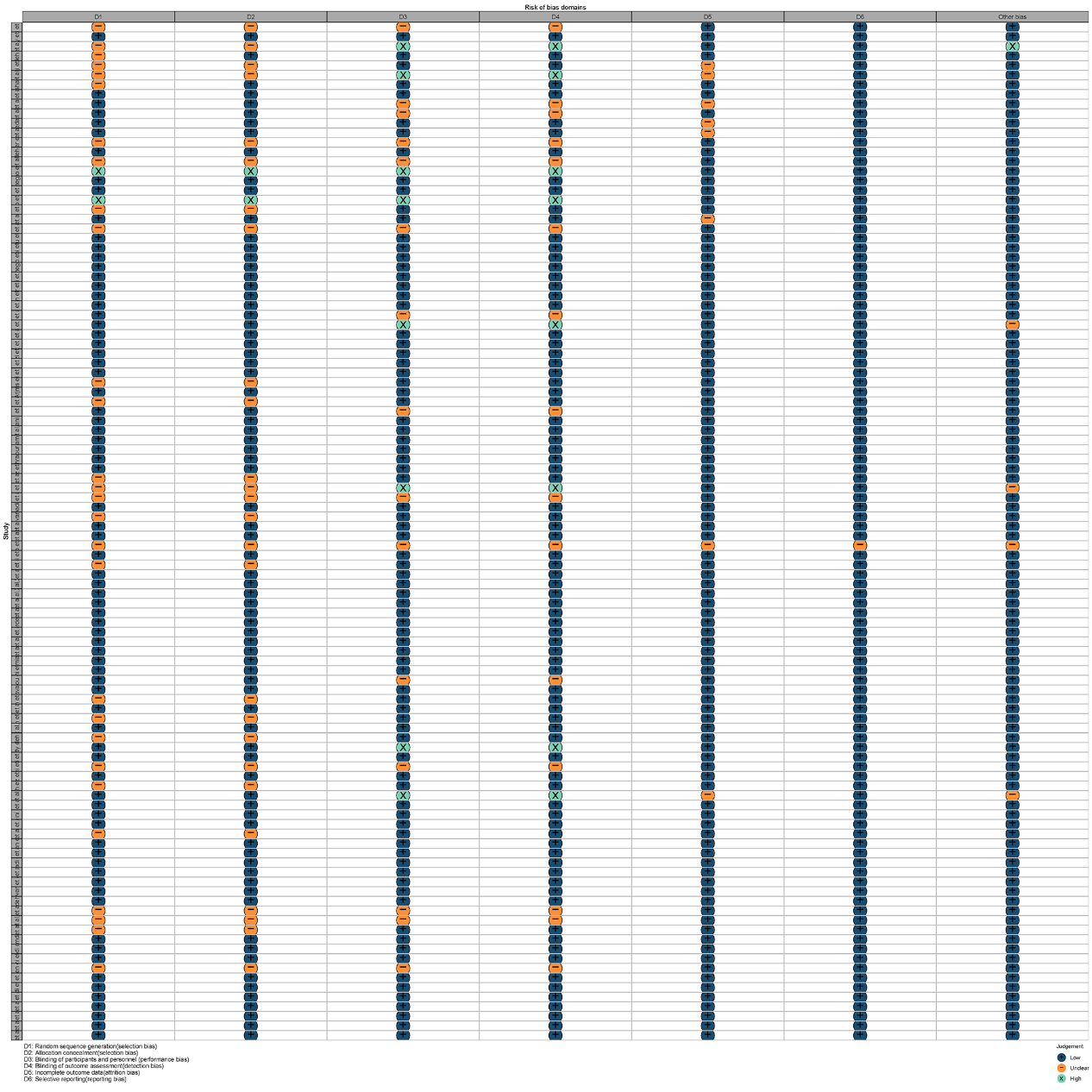
**

**Figure S1**. Risk of bias summary


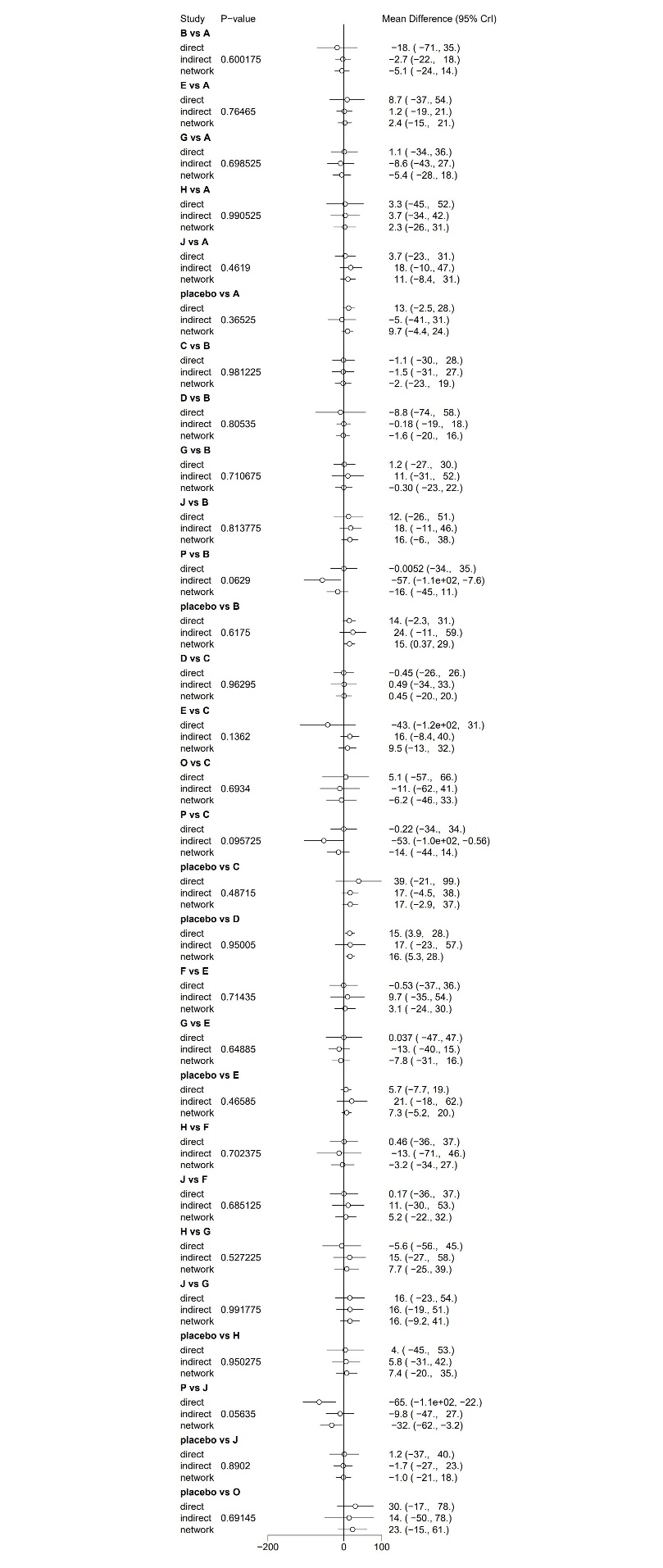


**Figure S2**. Node-splitting analysis diagram of TG


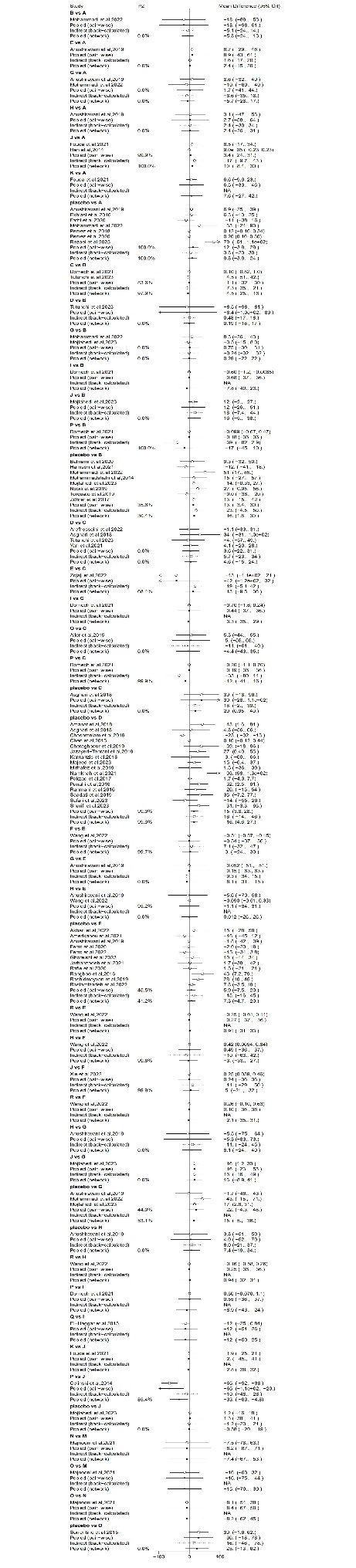


**Figure S3**. Heterogeneity forest plot for TG


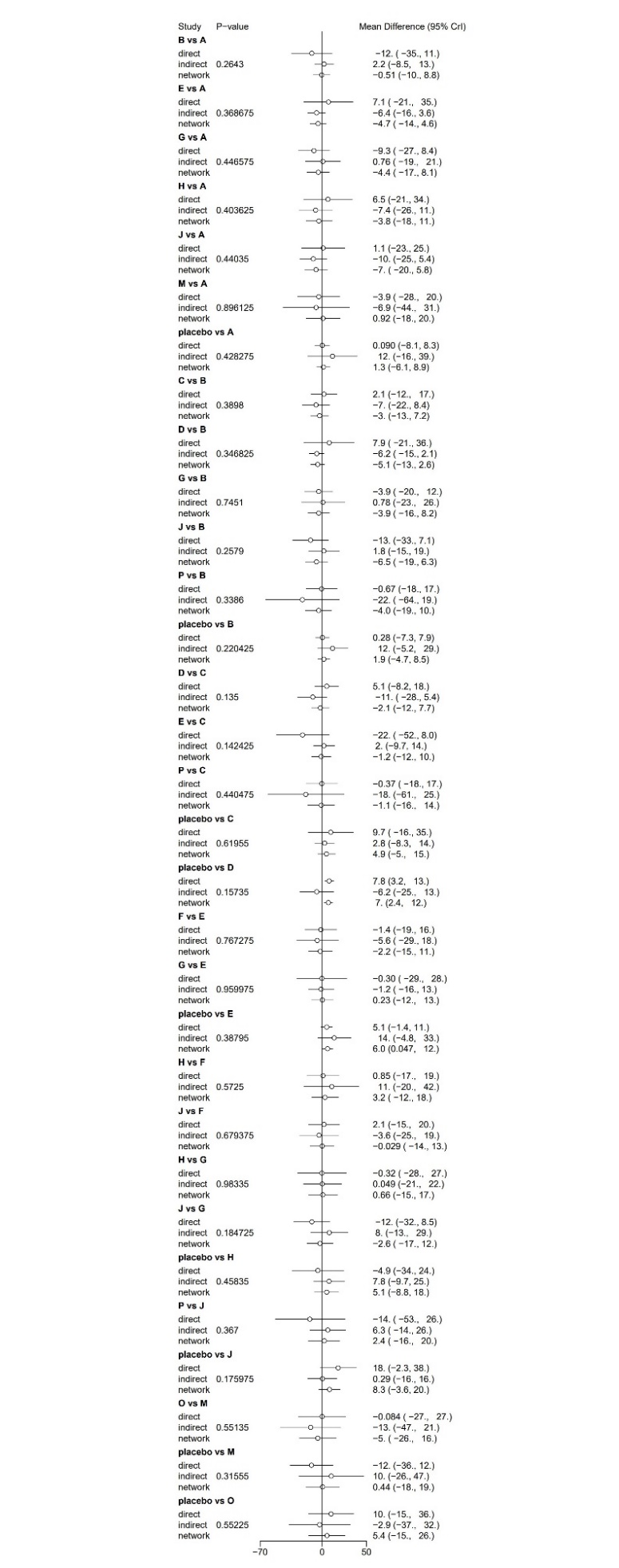


**Figure S4**. Node-splitting analysis diagram of TC


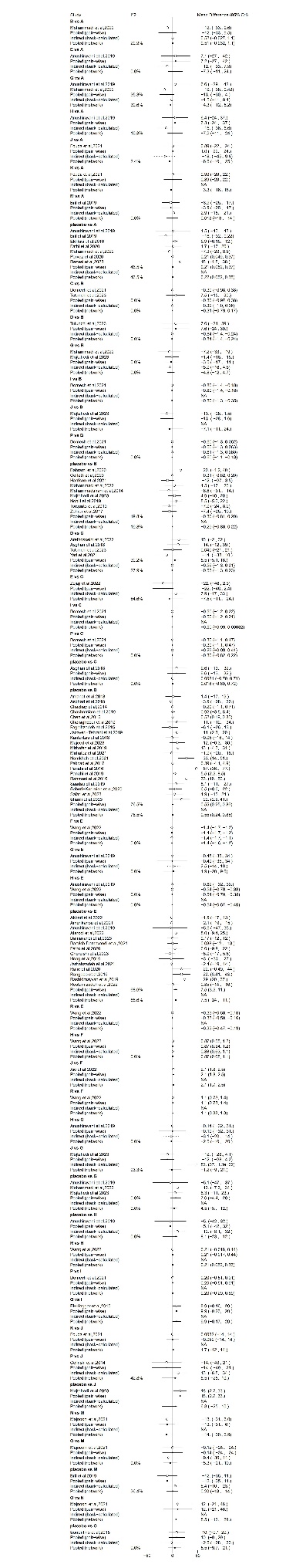


**Figure S5**. Heterogeneity forest plot for TC


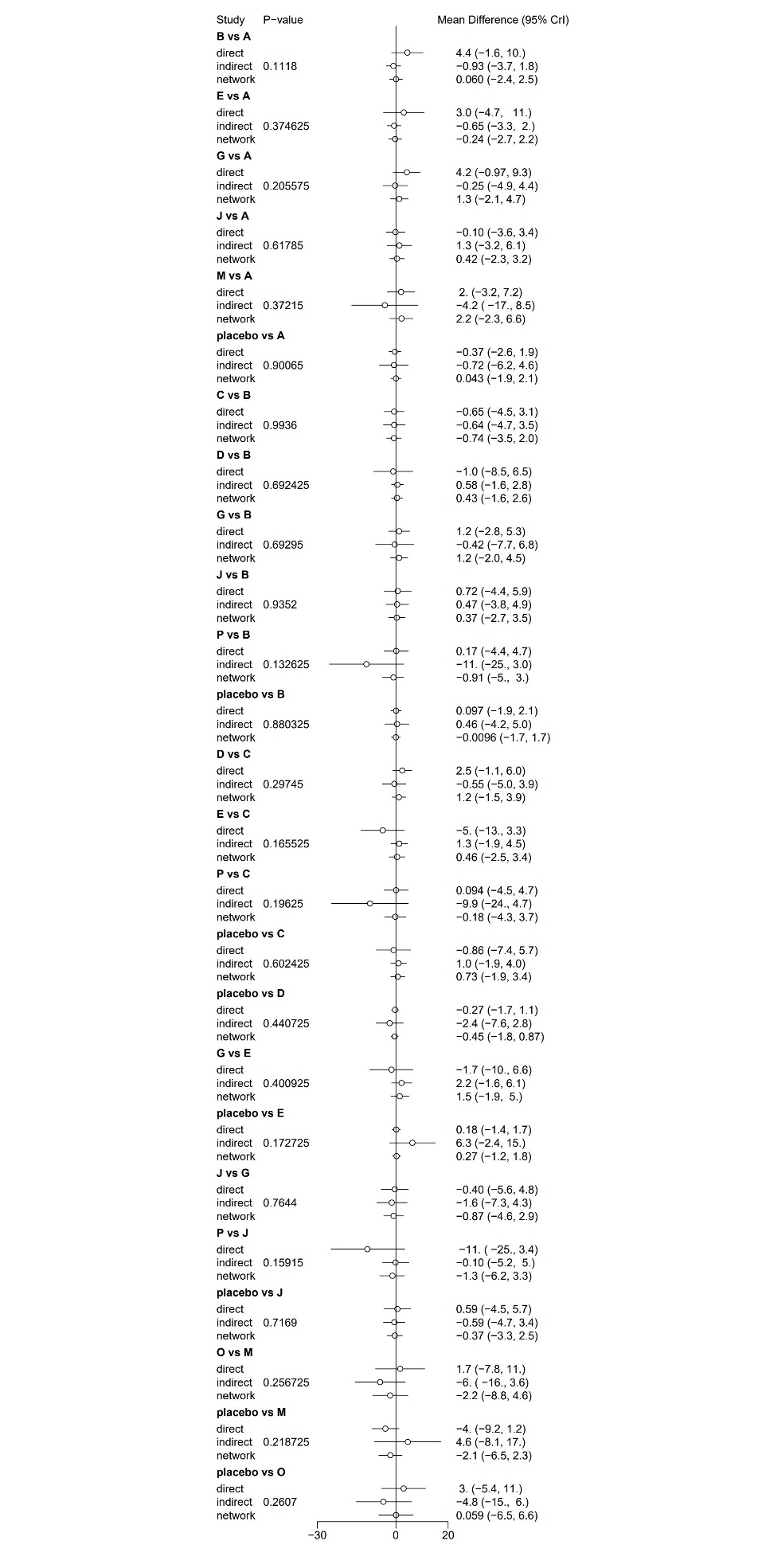


**Figure S6**. Node-splitting analysis diagram of HDL-C


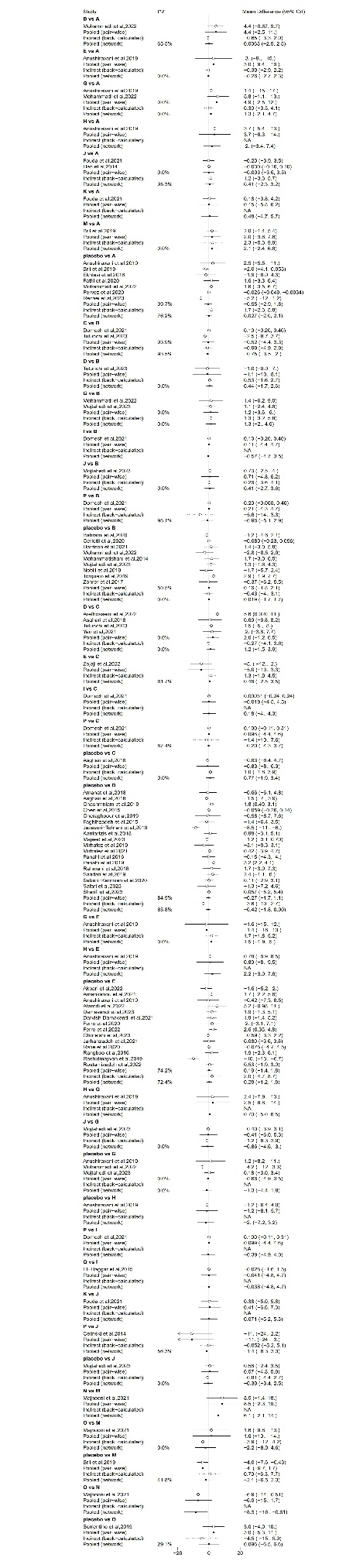


**Figure S7**. Heterogeneity forest plot for HDL-C


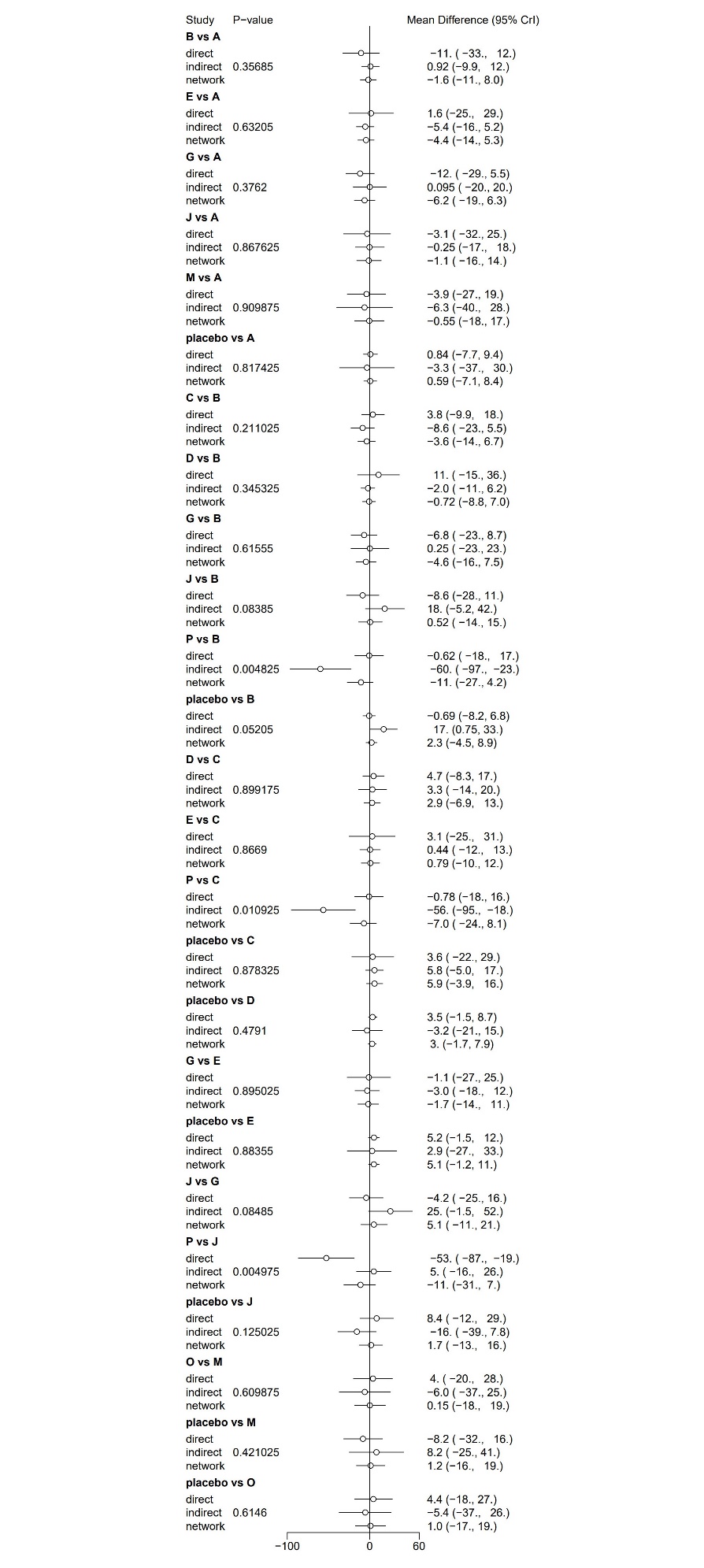


**Figure S8**. Node-splitting analysis diagram of LDL-C


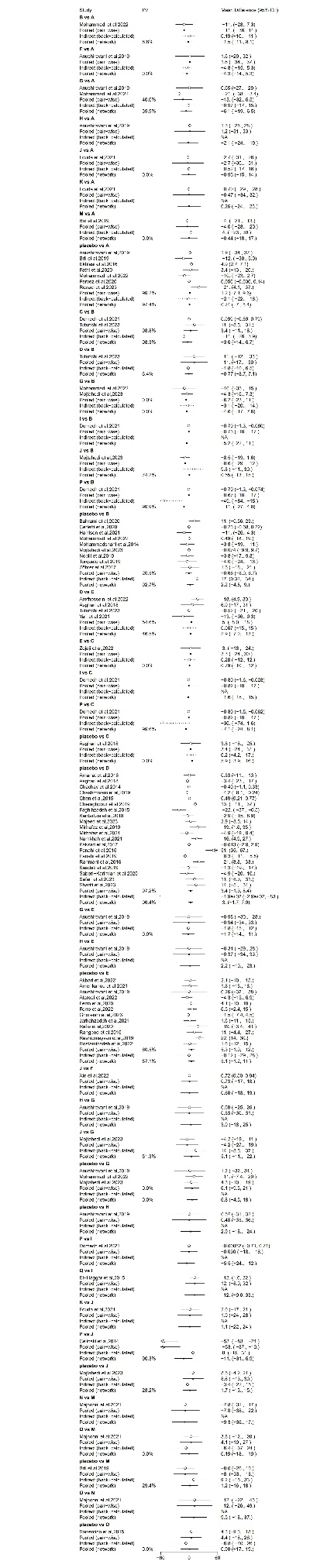


**Figure S9**. Heterogeneity forest plot for LDL-C


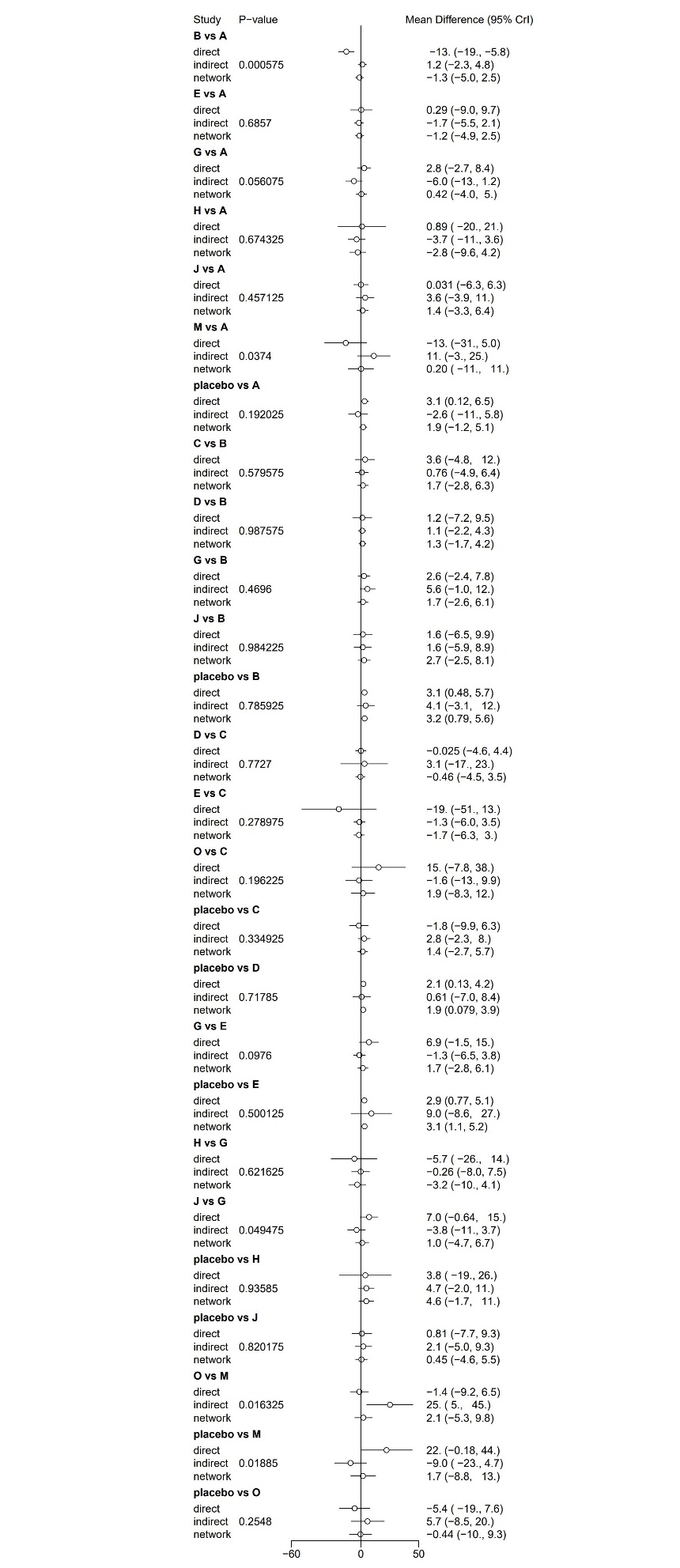


**Figure S10**. Node-splitting analysis diagram of FBG


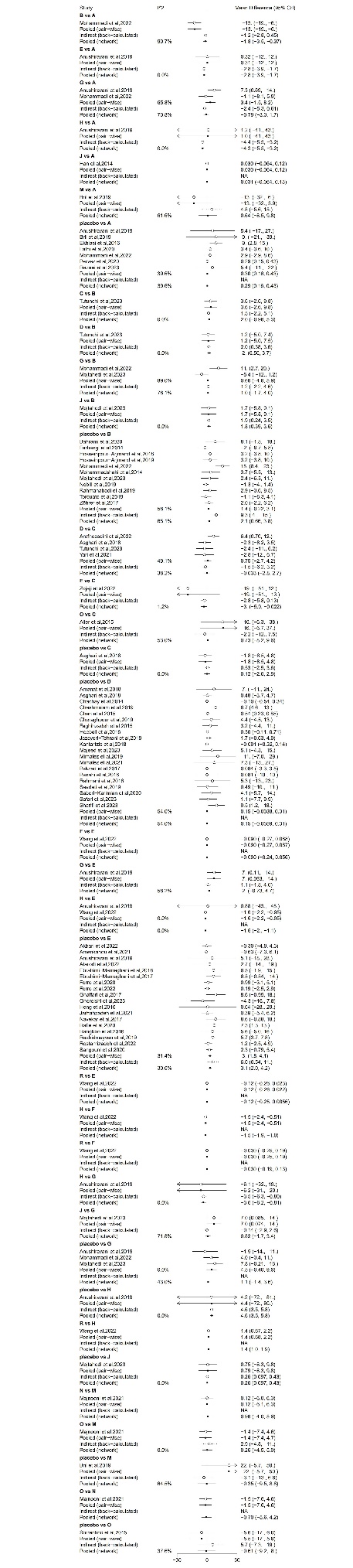


**Figure S11**. Heterogeneity forest plot for FBG


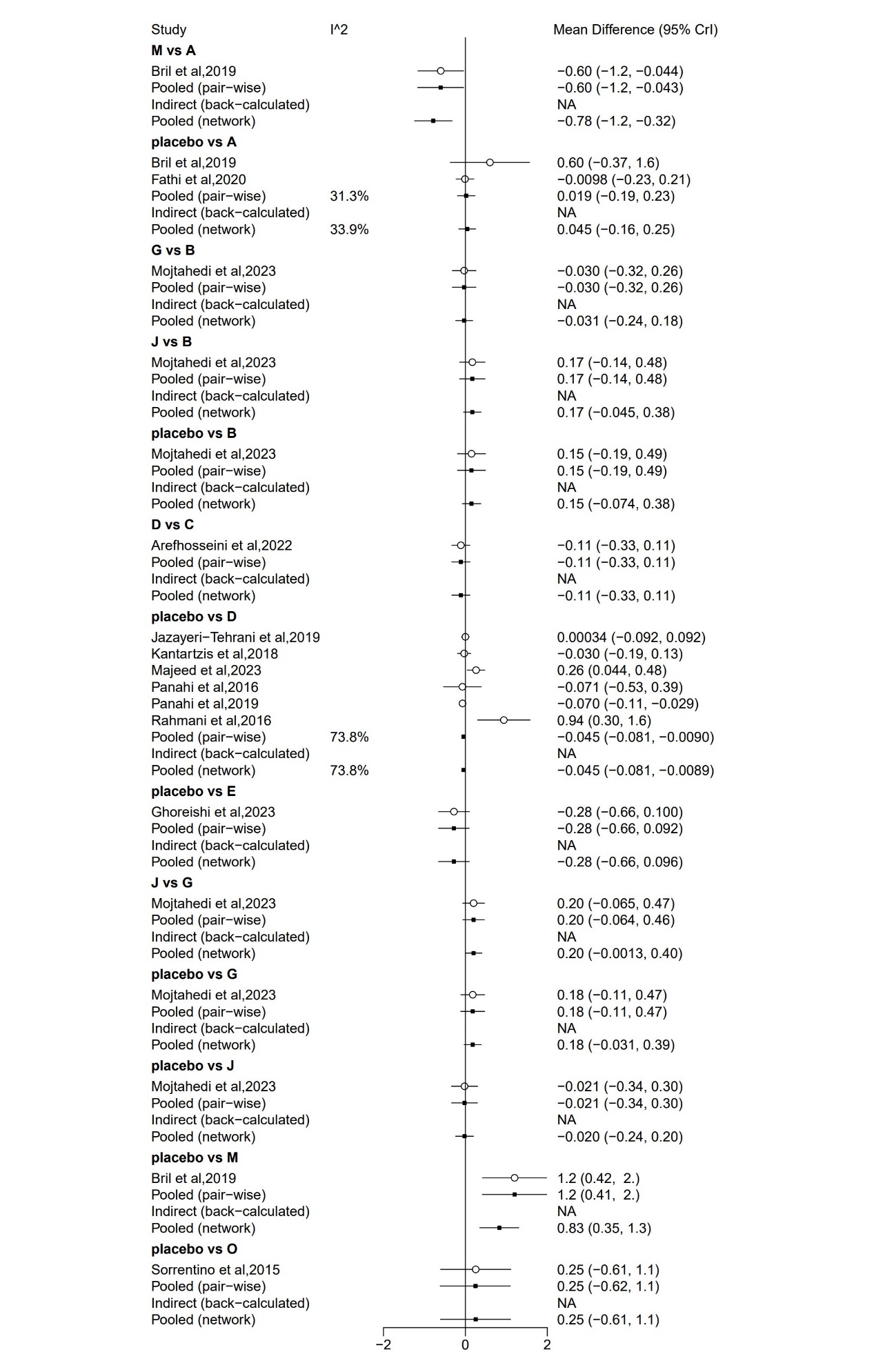


**Figure S12**. Heterogeneity forest plot for HbA1c


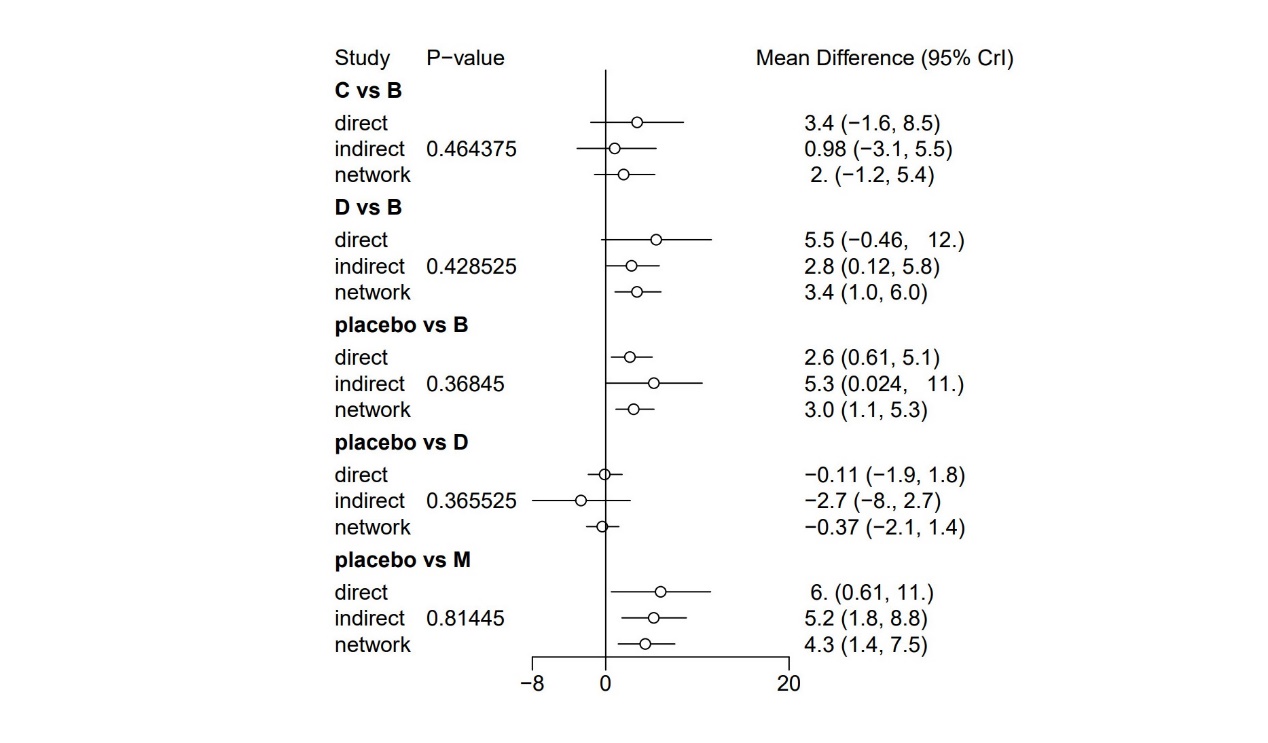


**Figure S13**. Node-splitting analysis diagram of Insulin


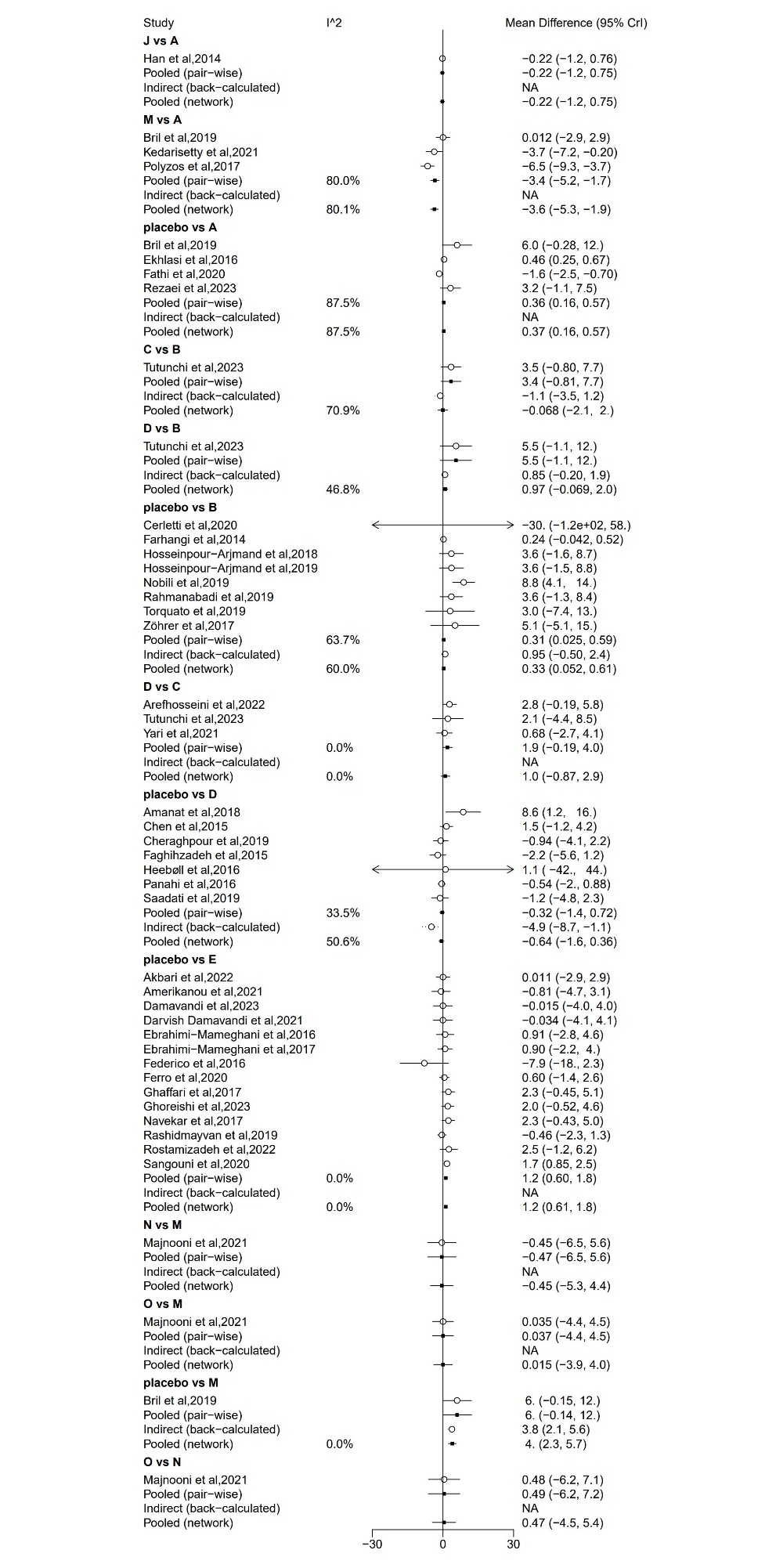


**Figure S14**. Heterogeneity forest plot for Insulin


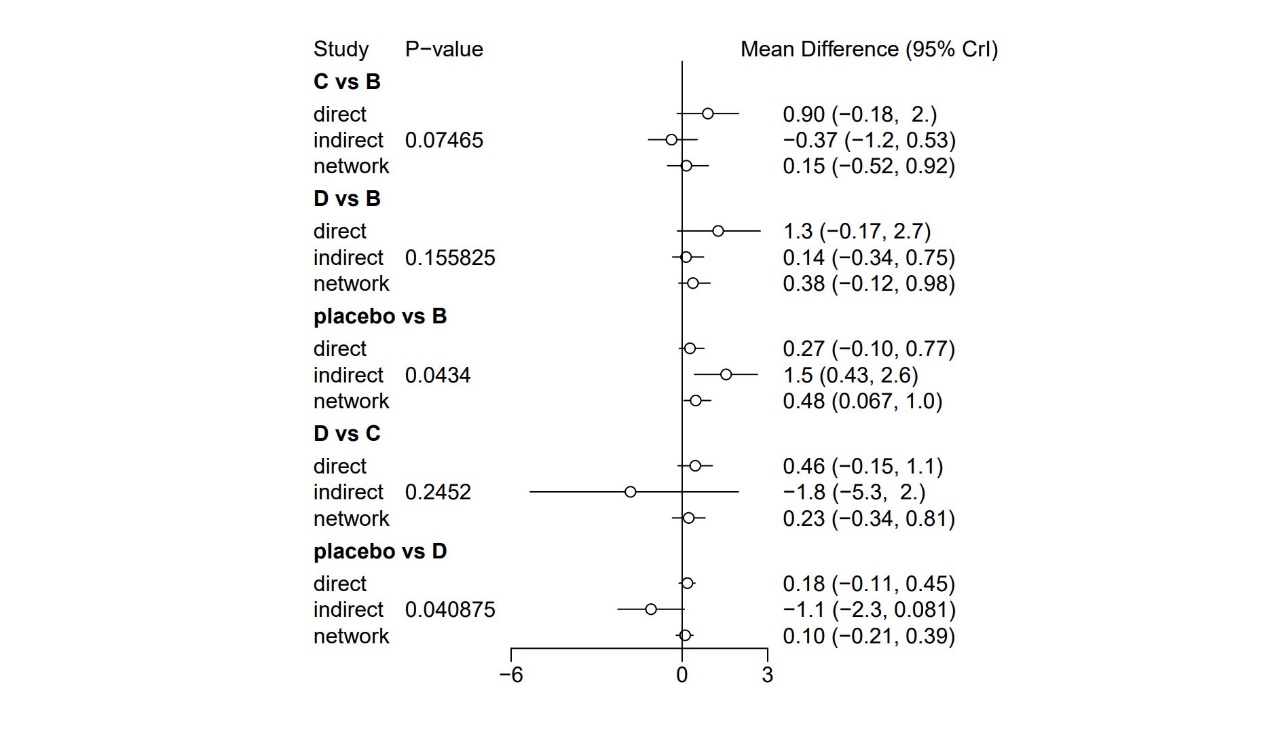


**Figure S15**. Node-splitting analysis diagram of HOMA-IR


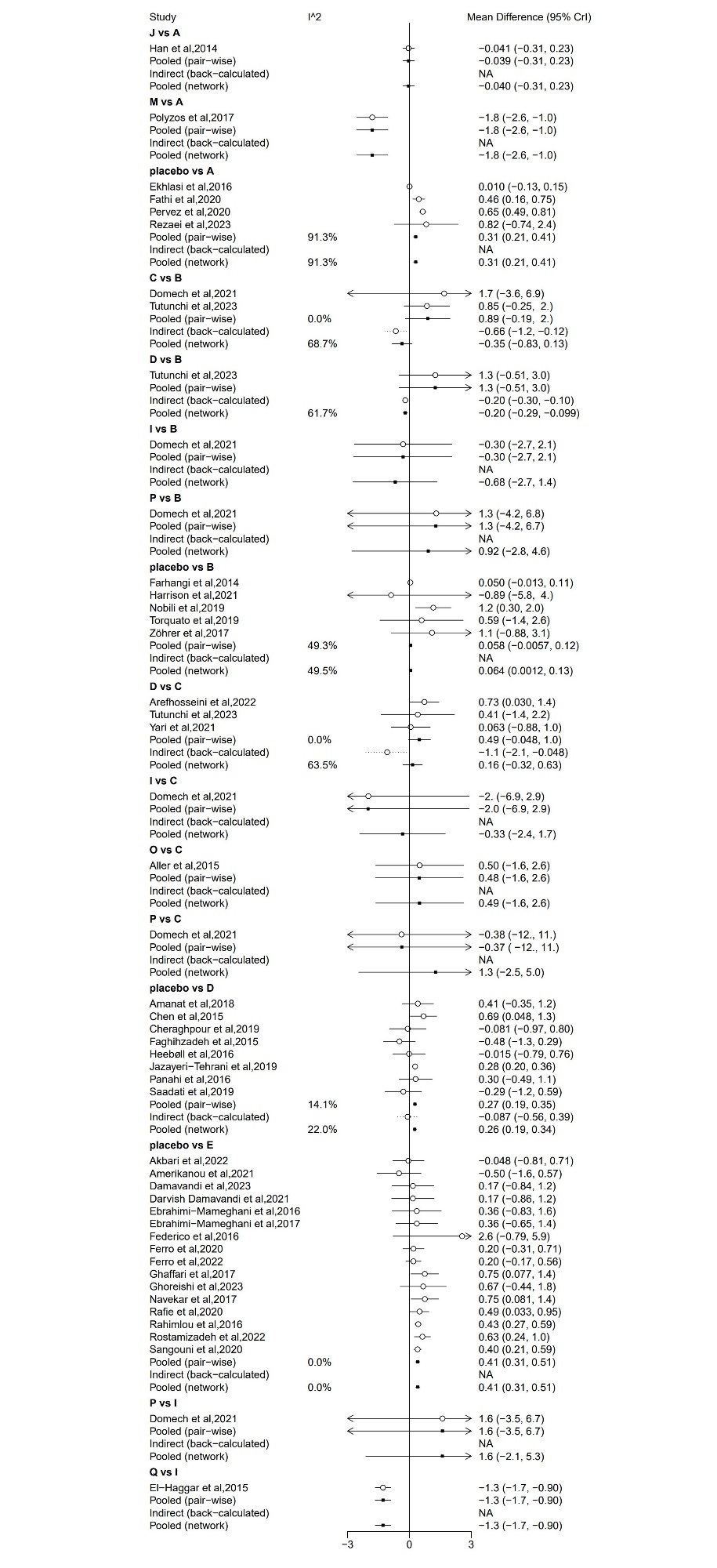


**Figure S16**. Heterogeneity forest plot for HOMA-IR


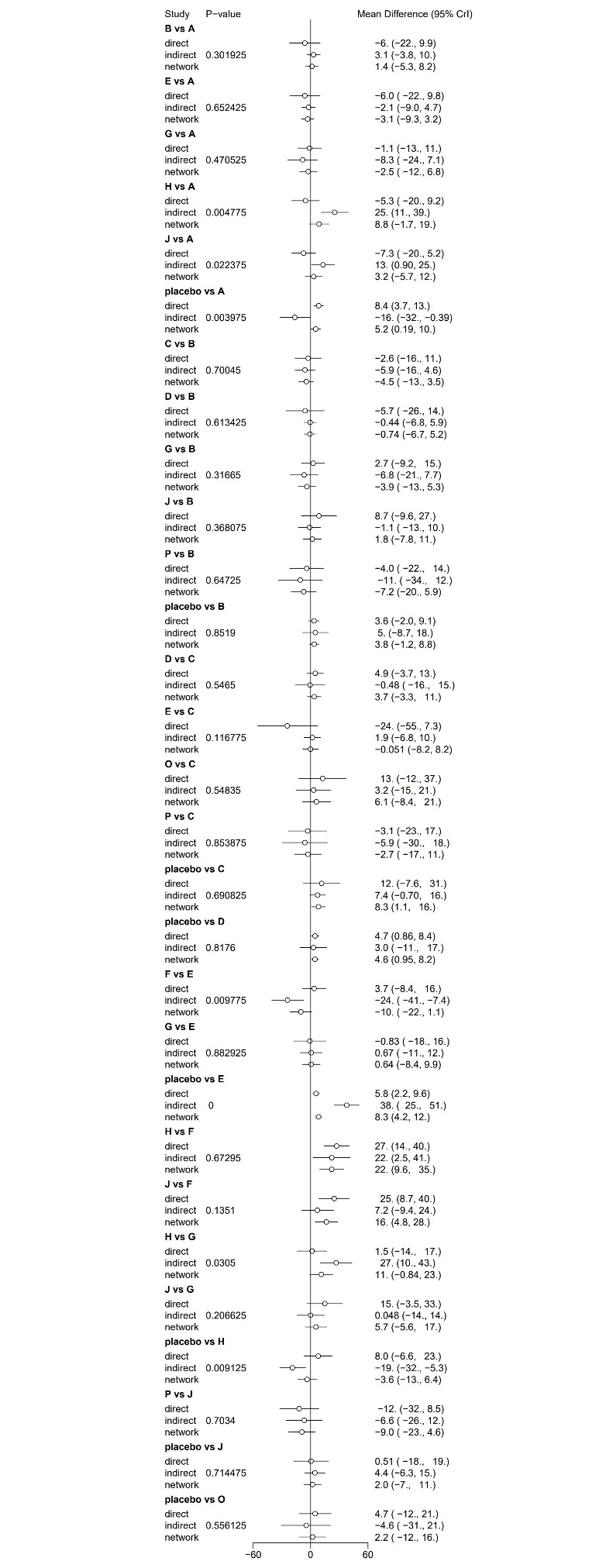


**Figure S17**. Node-splitting analysis diagram of ALT


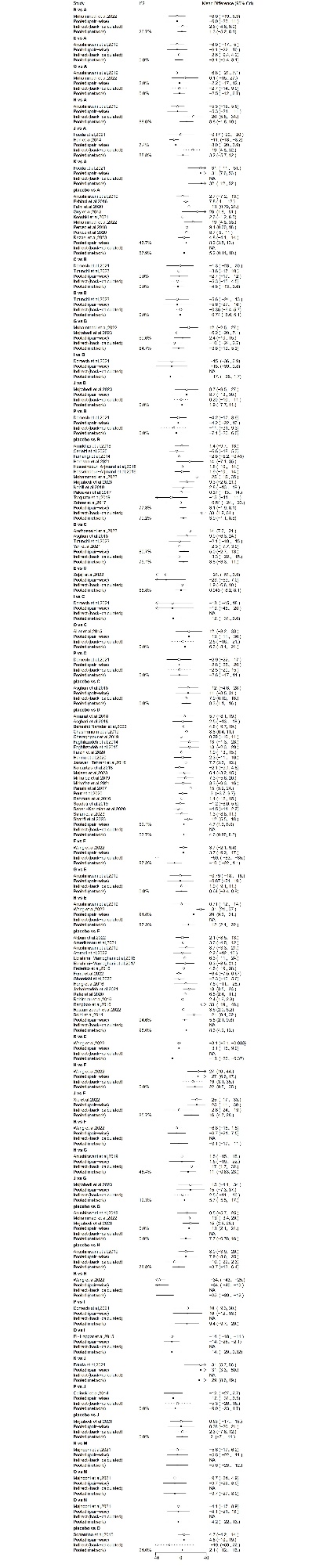


**Figure S18**. Heterogeneity forest plot for ALT


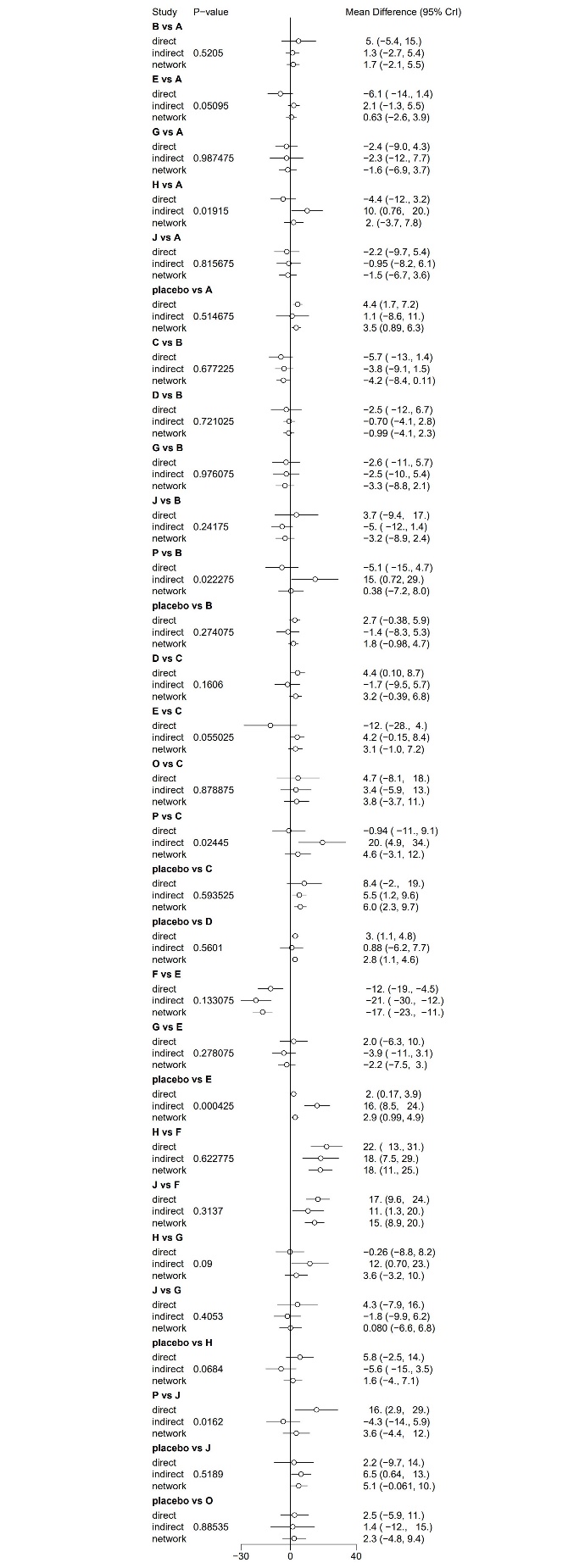


**Figure S19**. Node-splitting analysis diagram of AST


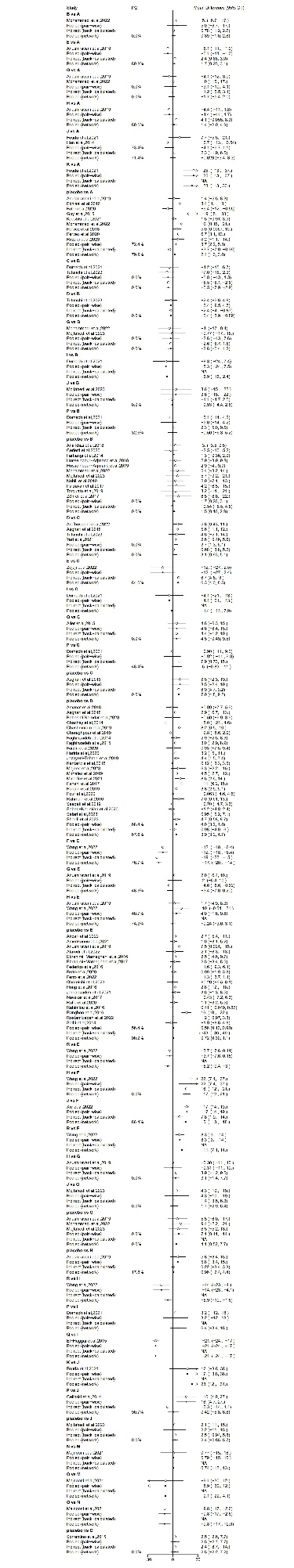


**Figure S20**. Heterogeneity forest plot for AST


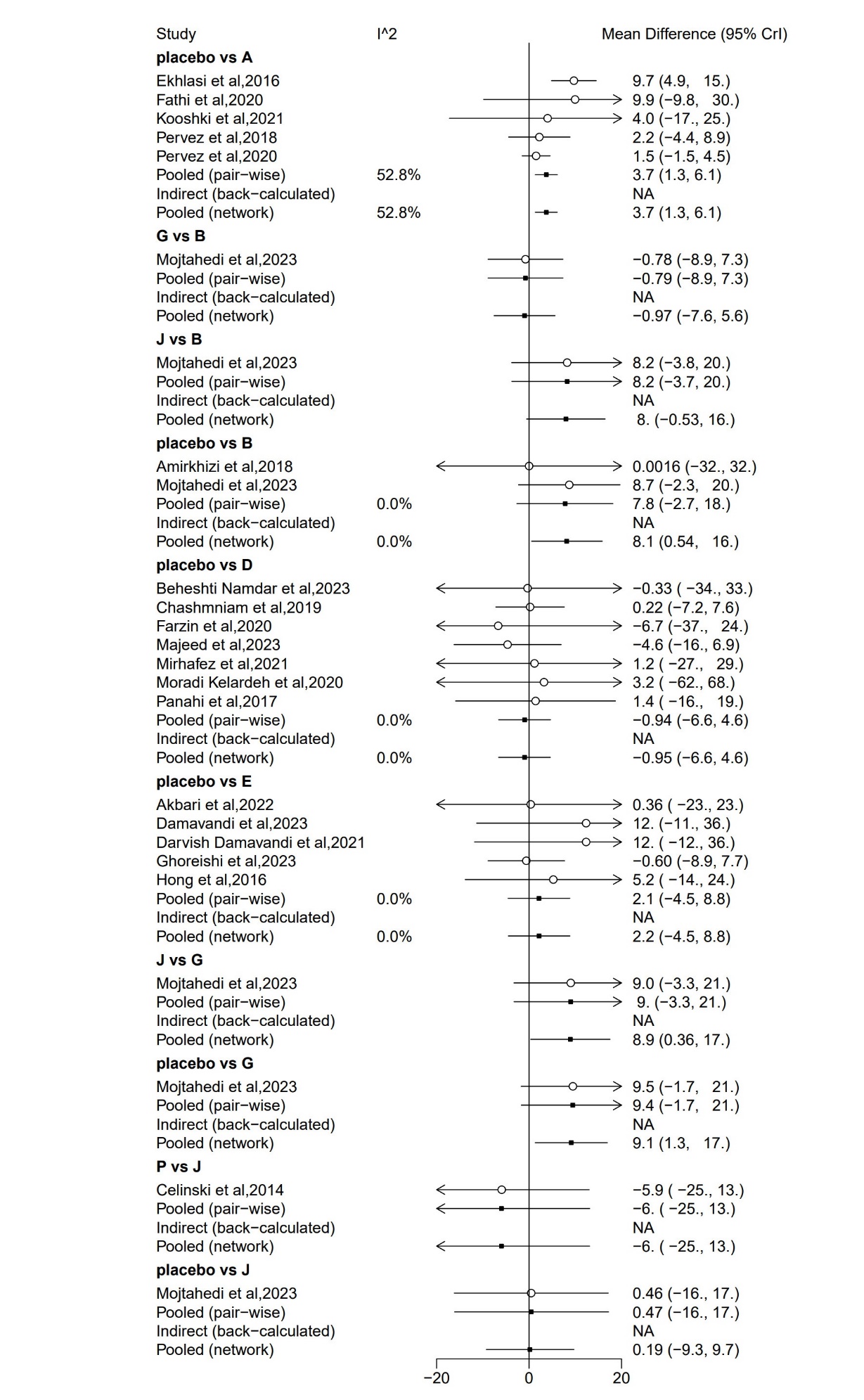


**Figure S21**. Heterogeneity forest plot for ALP


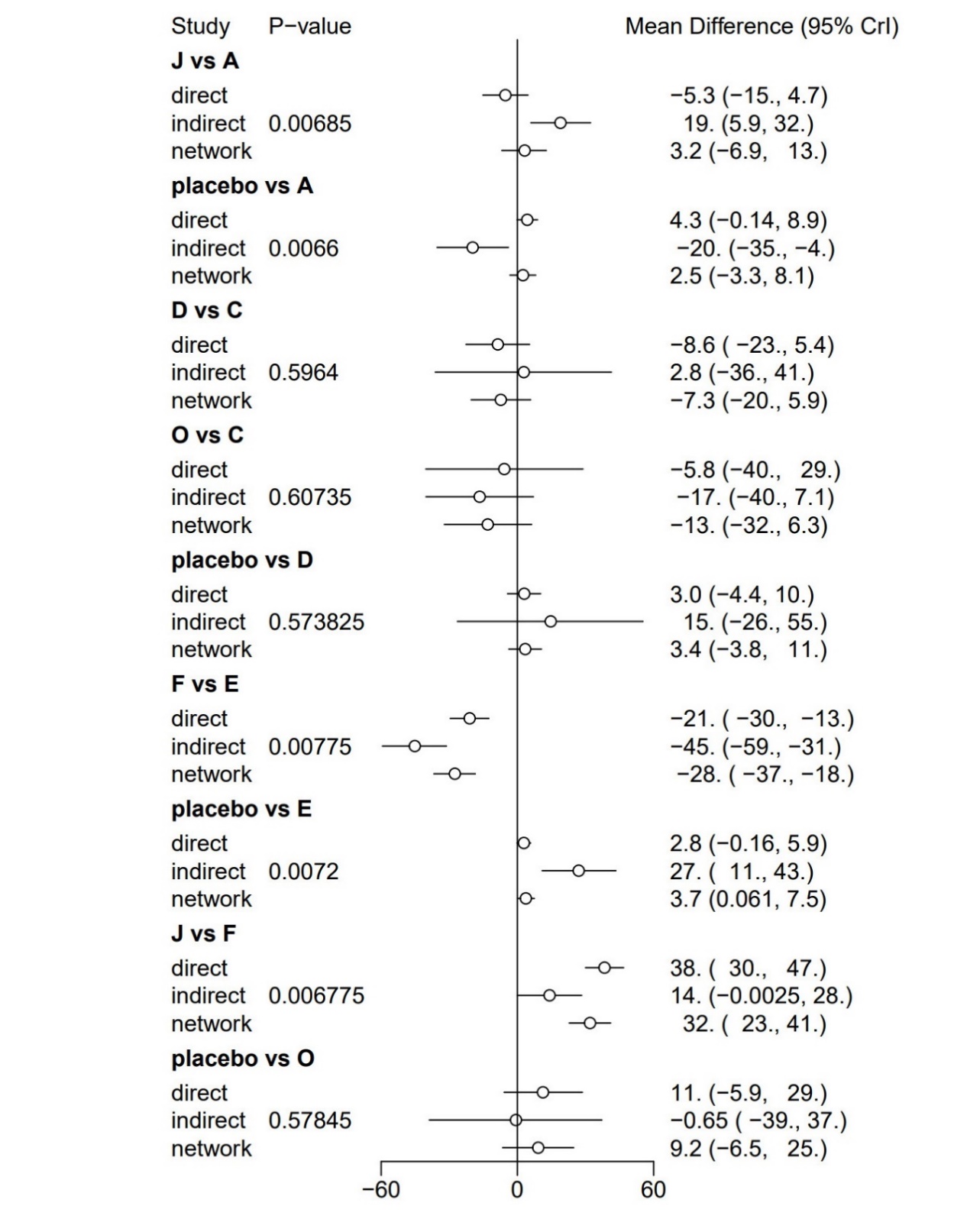


**Figure S22**. Node-splitting analysis diagram of GGT


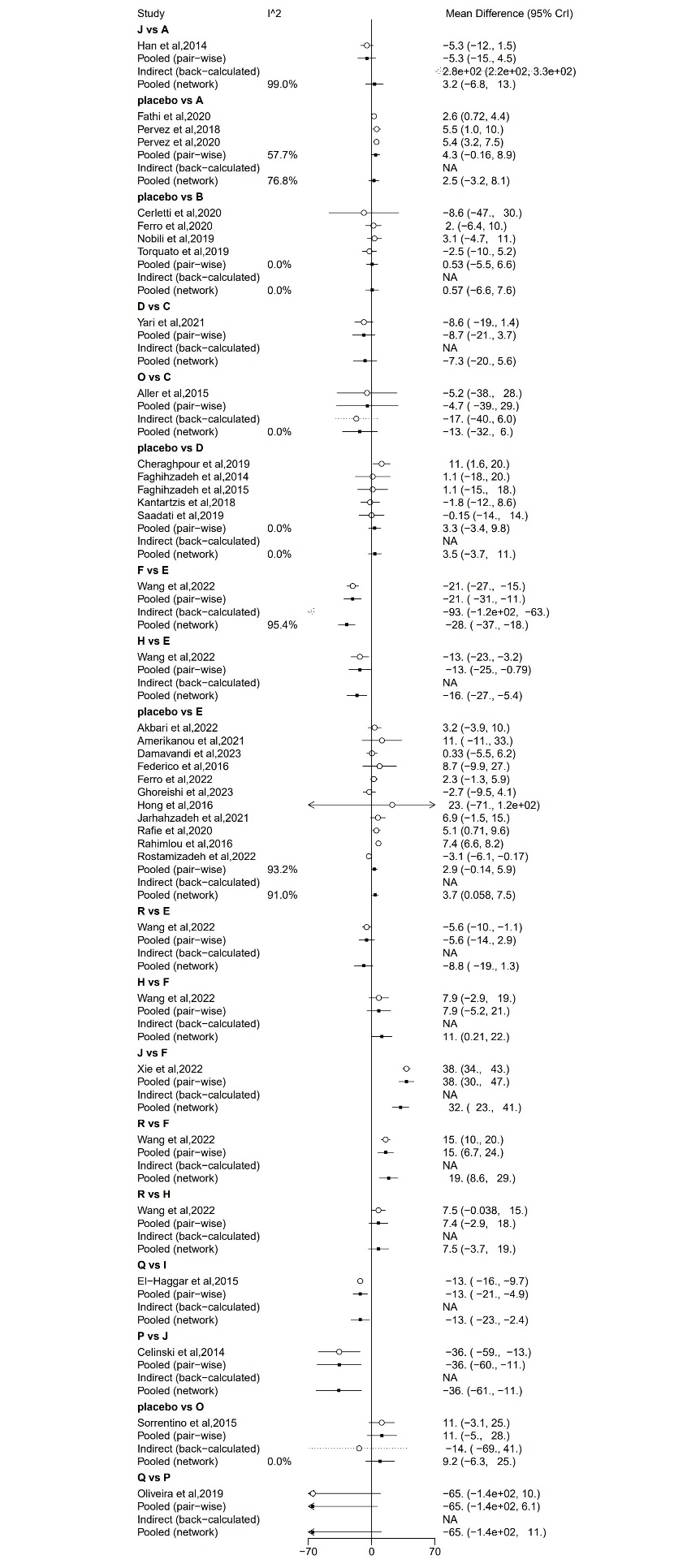


**Figure S23**. Heterogeneity forest plot for GGT


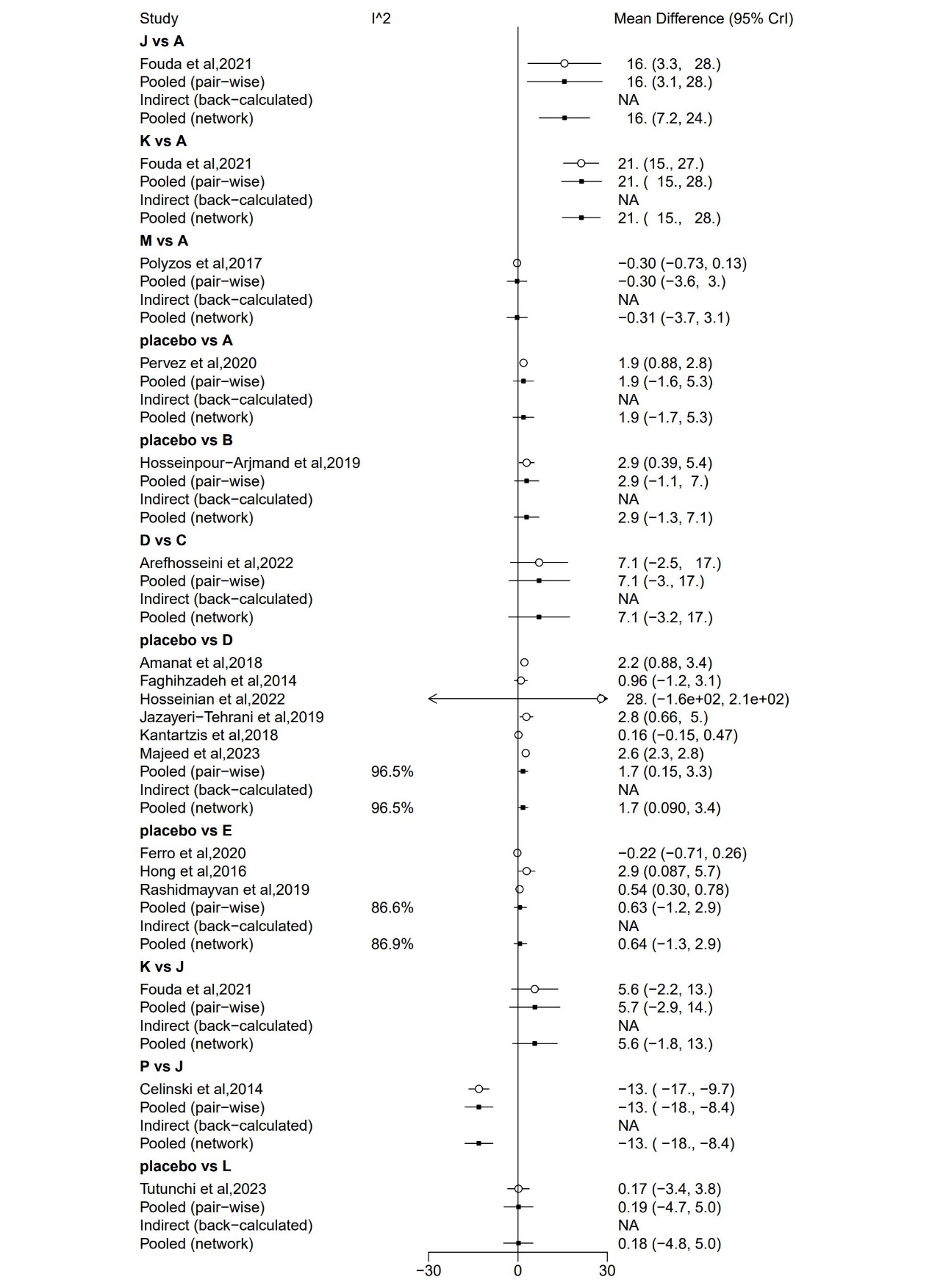


**Figure S24**. Heterogeneity forest plot for IL-6


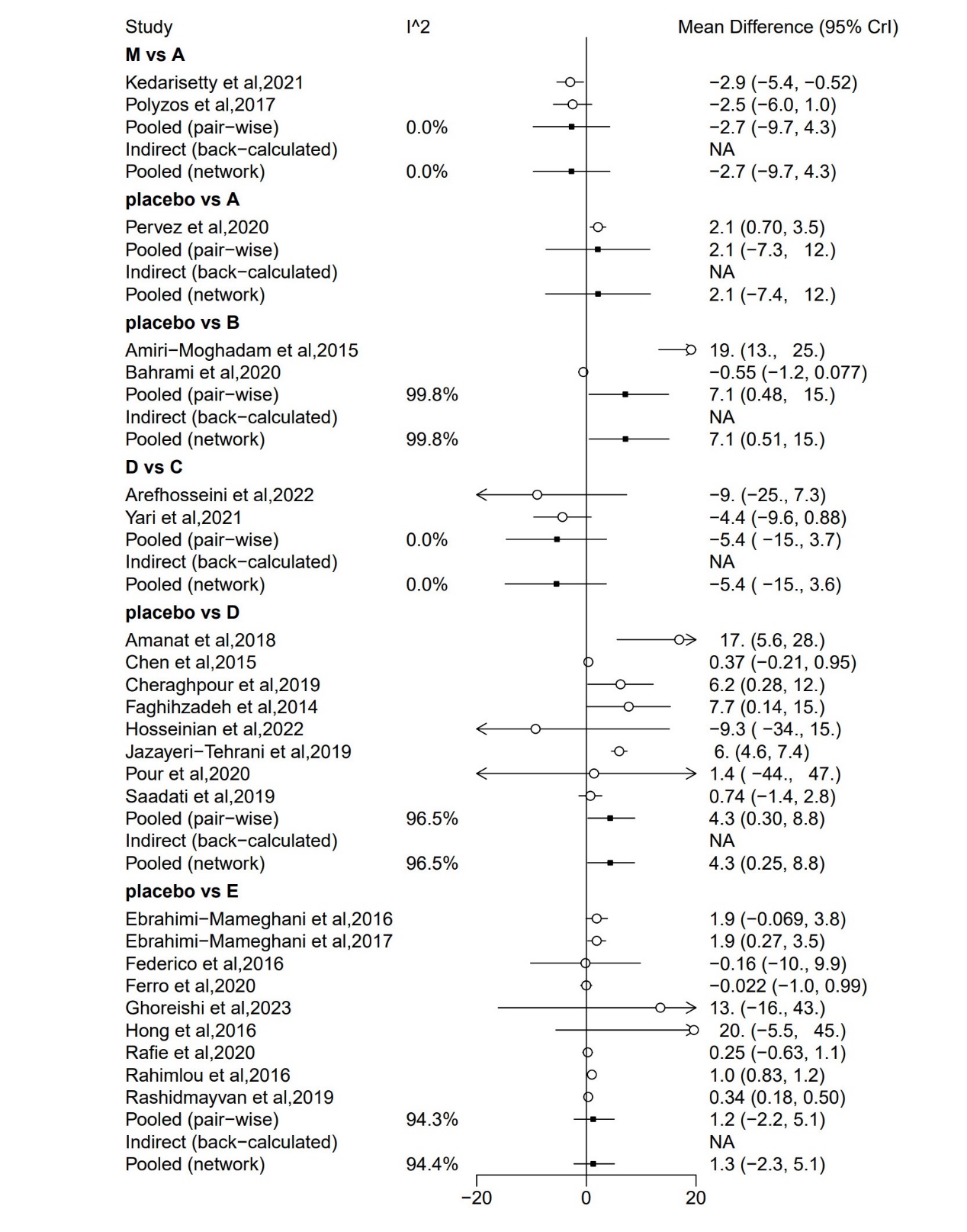


**Figure S25**. Heterogeneity forest plot for TNF-α


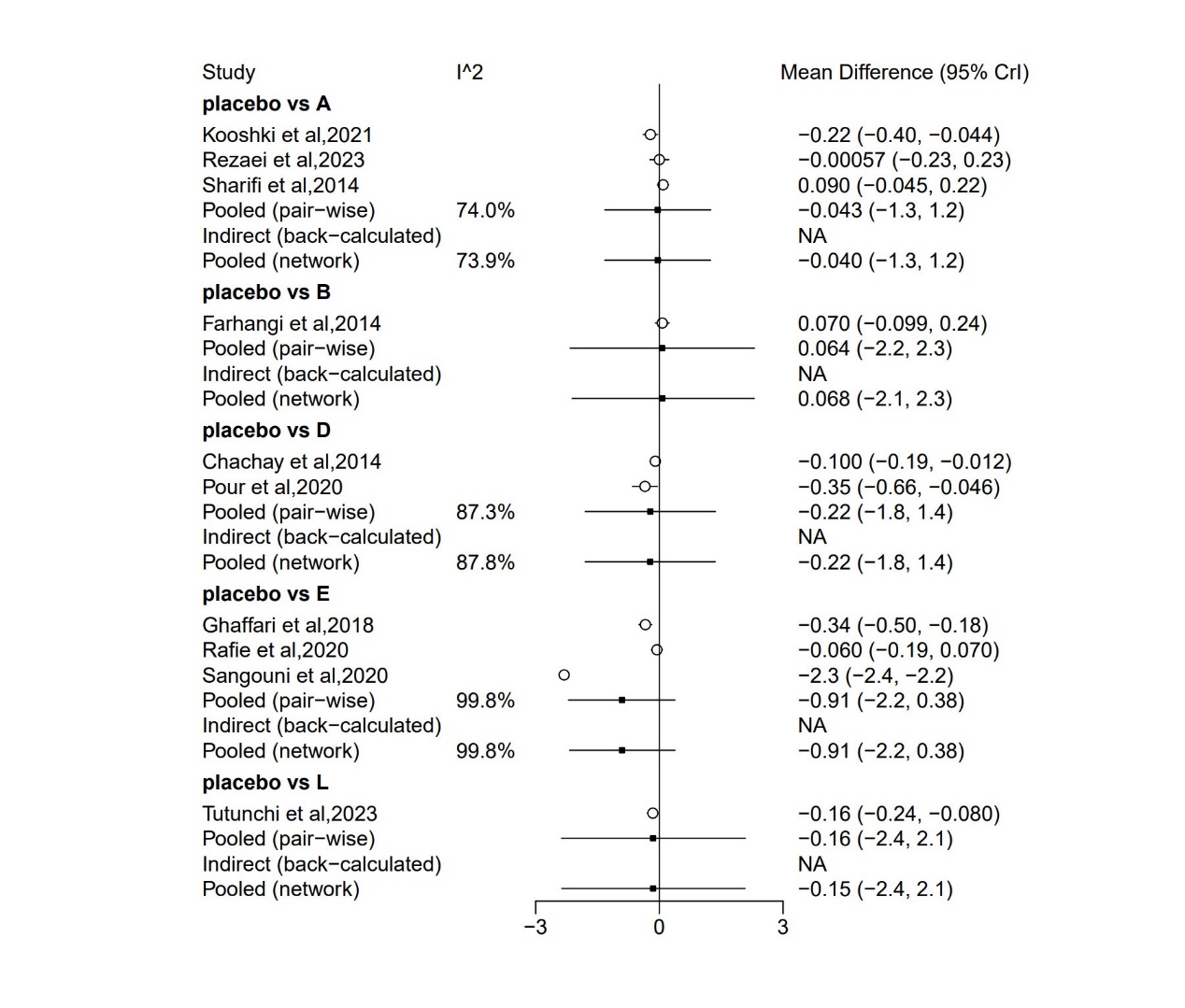


**Figure S26**. Heterogeneity forest plot for TAC


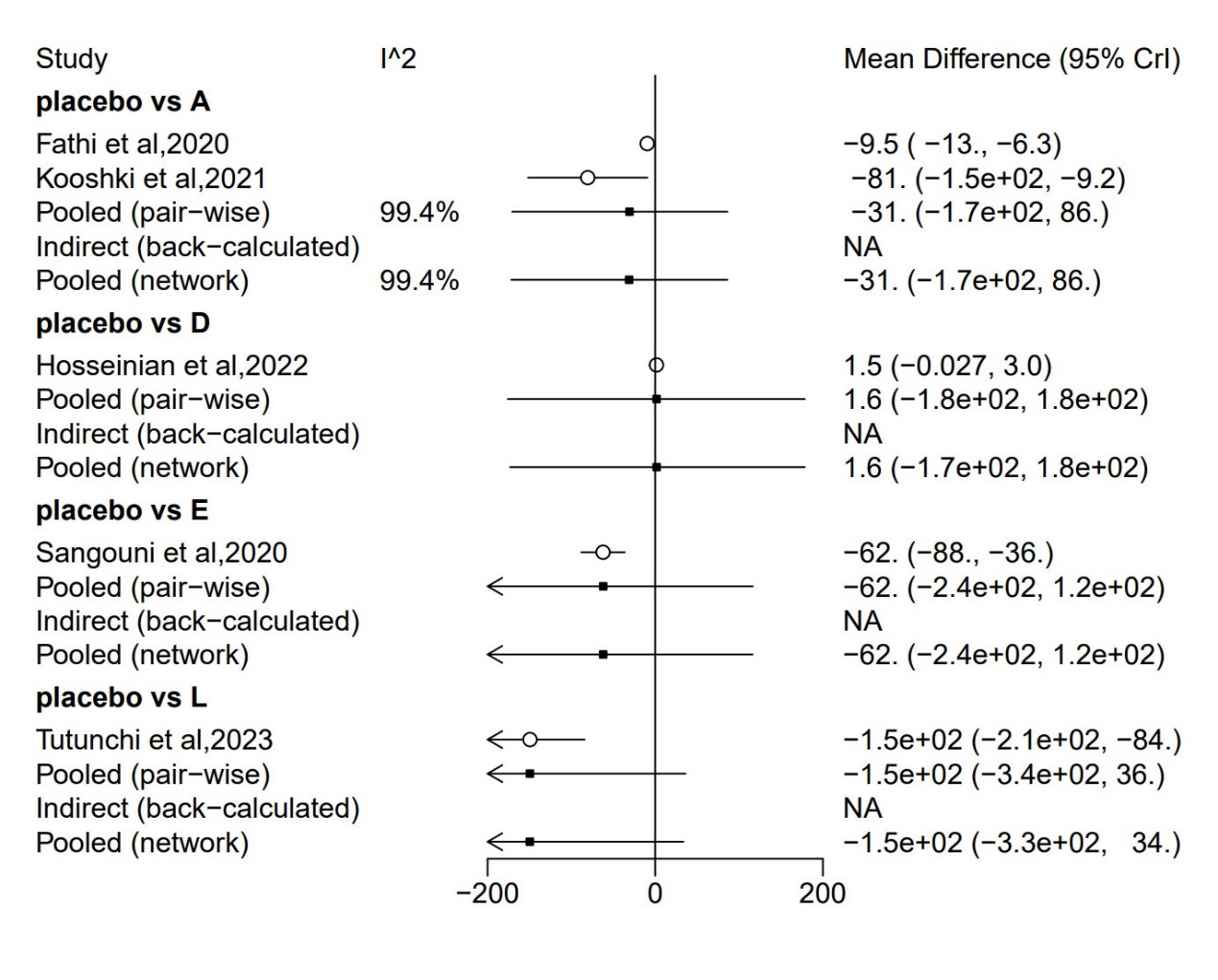


**Figure S27**. Heterogeneity forest plot for SOD


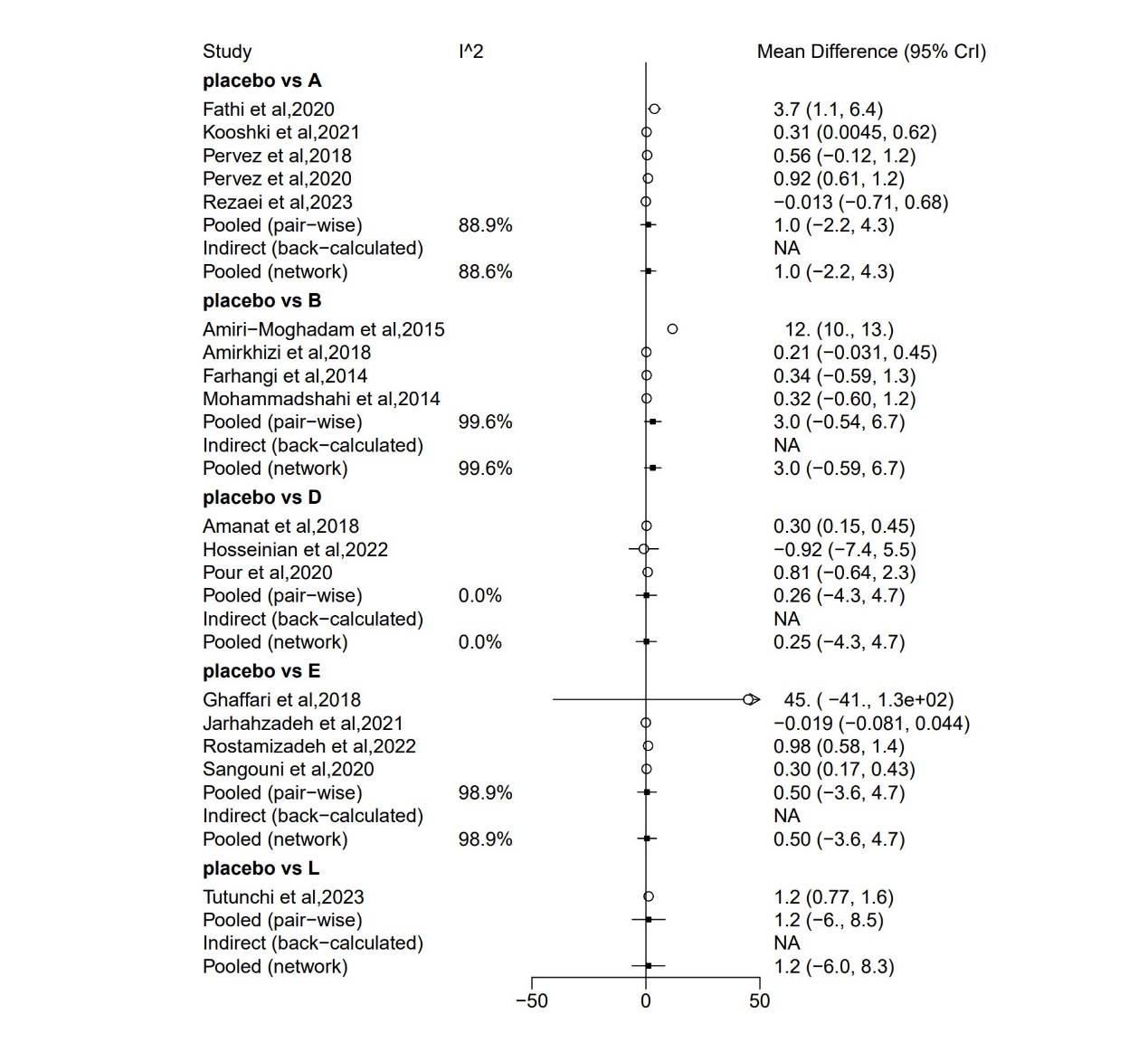


**Figure S28**. Heterogeneity forest plot for MDA

**
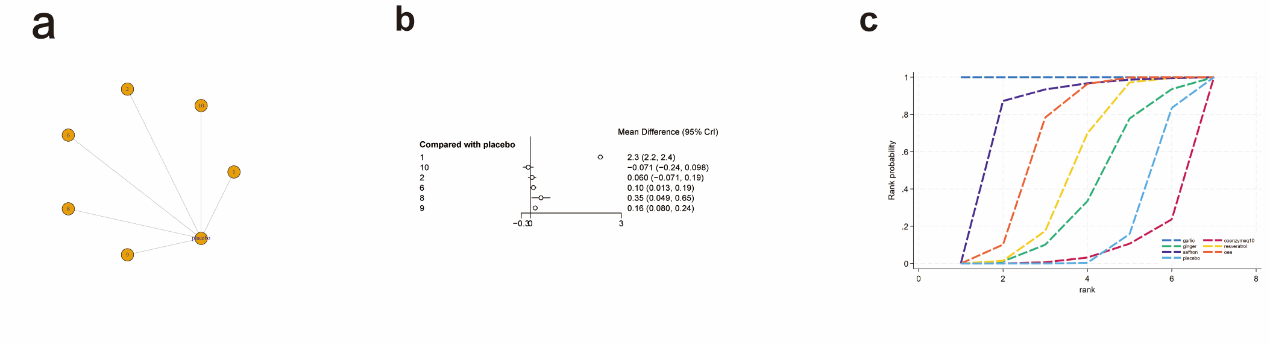
Figure S29.** Network analysis of different interventions on TAC. (a) Network evidence; (b) Forest plot; (c) SUCRA: cumulative ranking.

**
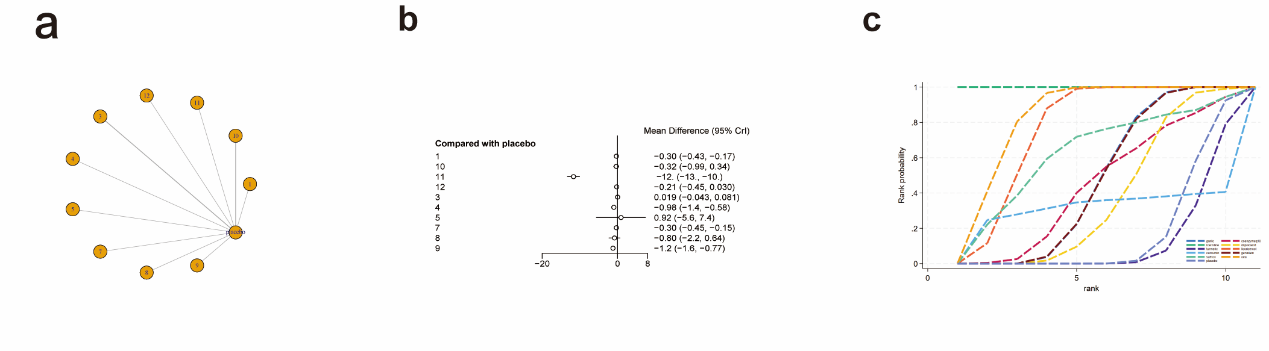
Figure S30.** Network analysis of different interventions on MDA. (a) Network evidence; (b) Forest plot; (c) SUCRA: cumulative ranking.


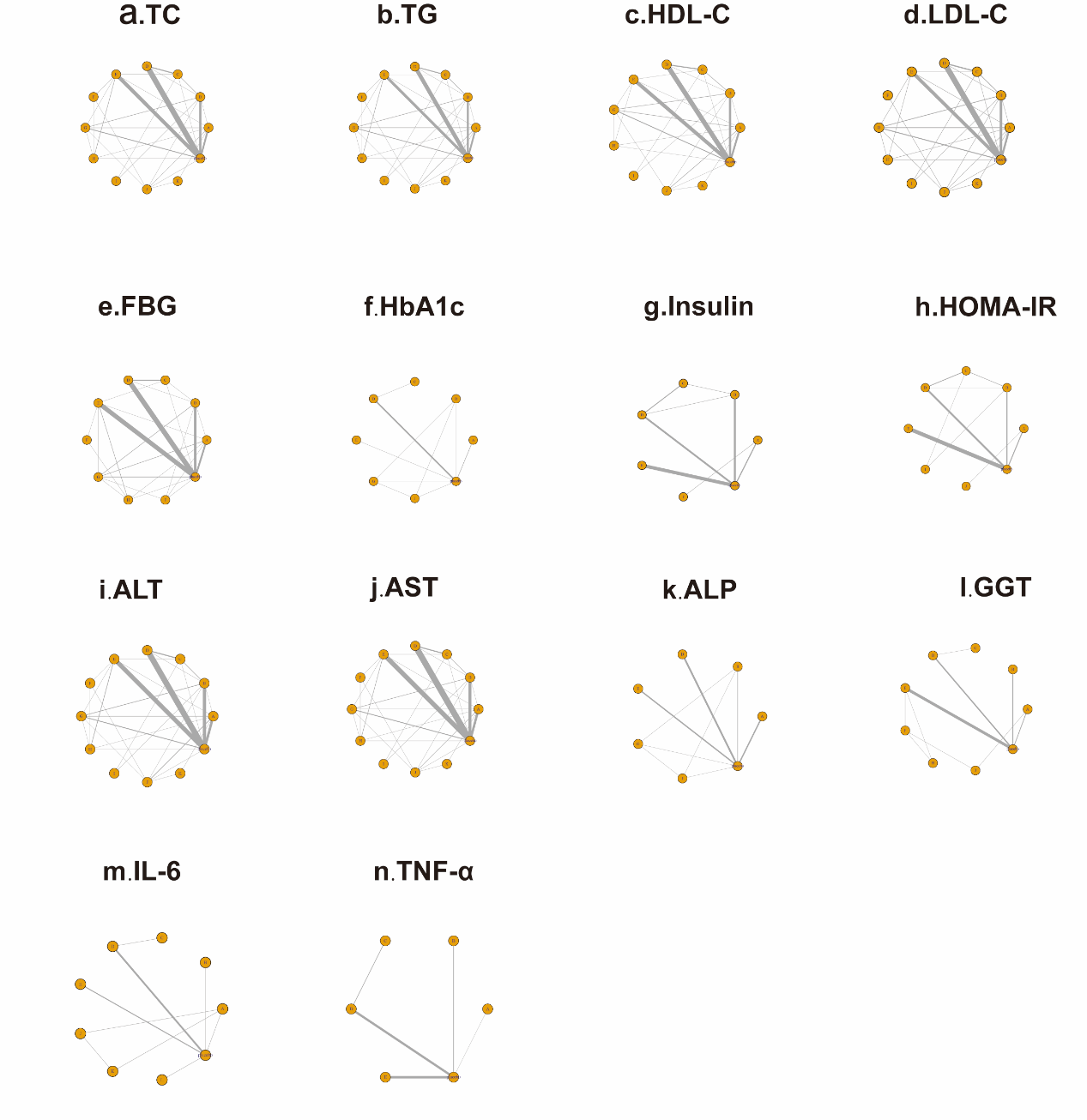


**Figure S31**. Network plot of the sensitivity analysis


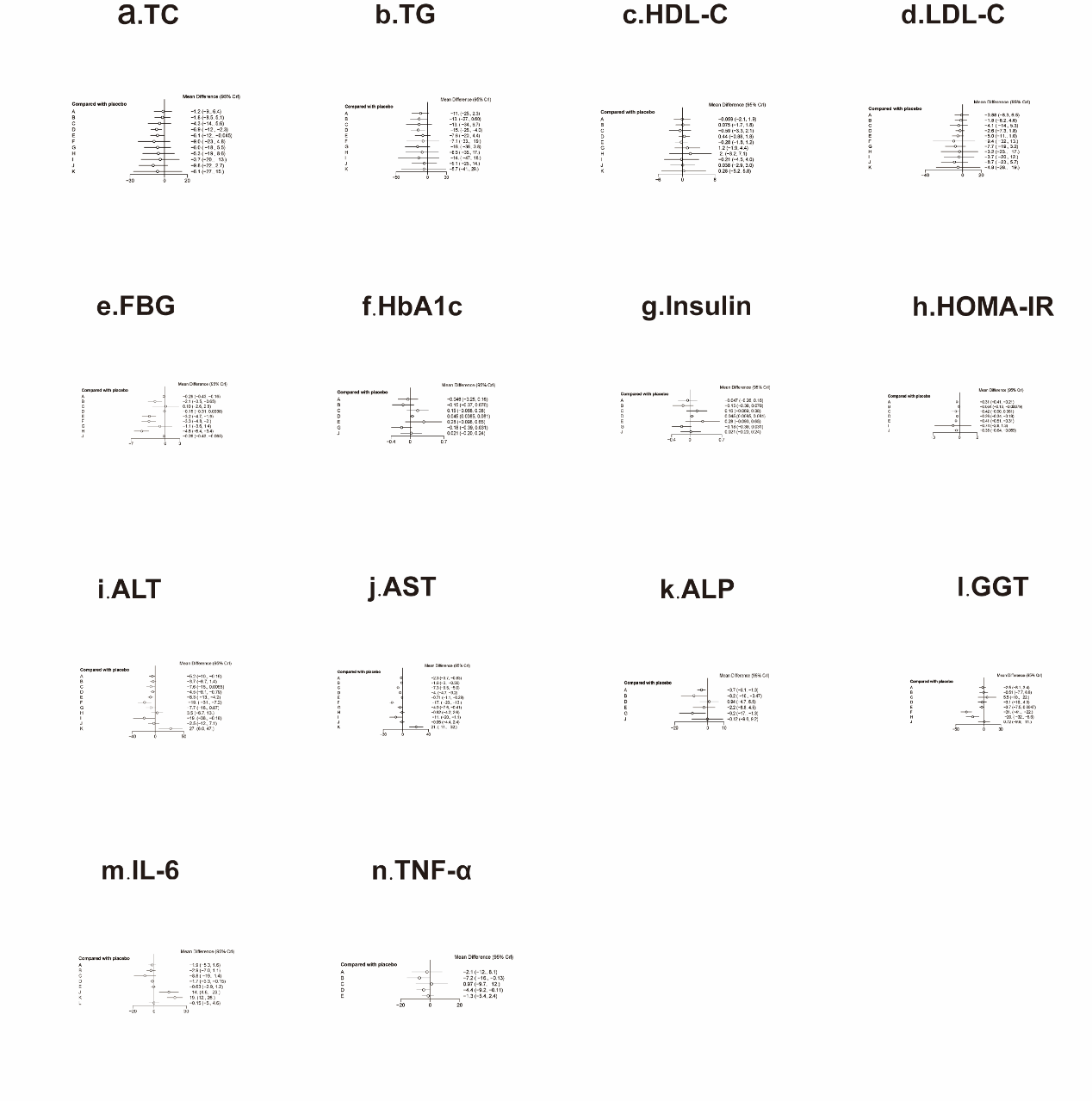


**Figure S32**. Forest plot of sensitivity analysis


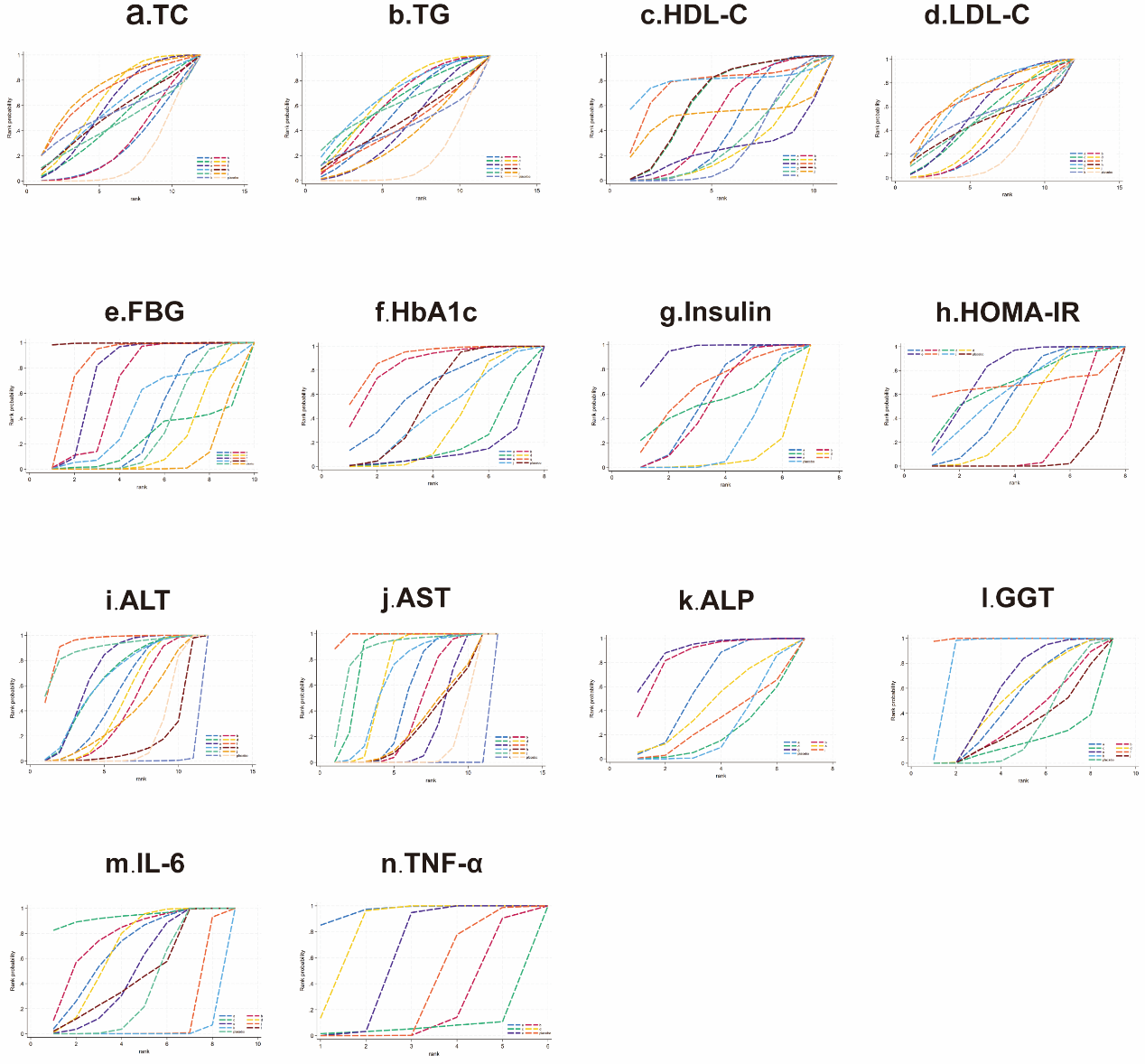


**Figure S33**. SUCRA plot of sensitivity analysis
